# Impact of precise modulation of reactive oxygen species levels on spermatozoa proteins in infertile men

**DOI:** 10.1186/1559-0275-12-4

**Published:** 2015-02-09

**Authors:** Ahmet Ayaz, Ashok Agarwal, Rakesh Sharma, Mohamed Arafa, Haitham Elbardisi, Zhihong Cui

**Affiliations:** Center for Reproductive Medicine, Glickman Urological & Kidney Institute, Cleveland Clinic, Cleveland, OH 44195 USA; Male Infertility Unit, Department of Urology, Hamad Hospital, Doha, Qatar

**Keywords:** Spermatozoa, Reactive oxygen species, Male infertility, Spermatozoa proteins, Proteomics, Bioinformatics

## Abstract

**Background:**

Elevated levels of reactive oxygen species (ROS) are detected in 25% to 80% of infertile men. They are involved in the pathology of male infertility. Understanding the effect of increasing levels of ROS on the differential expression of sperm proteins is important to understand the cellular processes and or/pathways that may be implicated in male infertility. The aim of this study was to examine differentially expressed proteins (DEPs) in spermatozoa from patients with low, medium and high ROS levels.

**Methods:**

A total of 42 infertile men presenting for infertility and 17 proven fertile men were enrolled in the study. ROS levels were measured by chemiluminescence assay. Infertile men were divided into Low (0- < 93 RLU/s/10^6^ sperm) (n = 11), Medium (>93-500 RLU/s/10^6^ sperm) (n = 17) and High ROS (>500 RLU/s/10^6^ sperm) group (n = 14). All fertile men had ROS levels between 4-50 RLU/s/10^6^ sperm. 4 subjects from fertile group and 4 each from the Low, Medium and High ROS were pooled. Protein extraction, protein estimation, gel separation of the proteins, in-gel digestion, LTQ-orbitrap elite hybrid mass spectrometry system was conducted. The DEPs, the cellular localization and pathways of DEPs involved were examined utilizing bioinformatics tools.

**Results:**

1035 proteins were identified in the 3 groups by global proteomic analysis. Of these, 305 were DEPs. 51 were unique to the Low ROS group, 47 Medium ROS group and 104 were unique to the High ROS group. 6 DEPs were identified by Uniprot and DAVID that had distinct reproductive functions and they were expressed only in 3 ROS groups but not in the control.

**Conclusions:**

We have for the first time demonstrated the presence of 6 DEPs with distinct reproductive functions only in men with low, medium or high ROS levels. These DEPs can serve as potential biomarkers of oxidative stress induced male infertility.

**Electronic supplementary material:**

The online version of this article (doi:10.1186/1559-0275-12-4) contains supplementary material, which is available to authorized users.

## Background

Infertility is defined as the inability to achieve a pregnancy after one year of unprotected intercourse [1]. Male factor infertility accounts for almost 40% of infertility problems, and another 40% is attributed to the female, while in about 20%, it is due to both male and female factors [2]. The etiology of male infertility is multifactorial, and in about 20% of the cases, the etiology is unknown or idiopathic [3]. Varicocele is present in about 15% of the male population, and in about 40% of men presenting with male infertility, which makes it the most common cause of male infertility [4]. The majority of the causes of male infertility are treatable or preventable, so a keen understanding of these conditions is paramount [5].

Oxidative stress results from the imbalance between reactive oxygen species (ROS) and the antioxidants that neutralize these excessive free radicals [6]. Oxidative stress results in abnormal semen parameters and [7] is strongly correlated with male infertility [8, 9]. When used in conjunction with conventional semen analysis, oxidative stress can help differentiate between fertile and infertile men, as well as help to identify a subgroup of infertile men who may benefit from antioxidant supplementation [7]. Factors such as lifestyle (smoking), environmental (pesticide, air pollution, electromagnetic radiation), and health (chemotherapy, urogenital infection, prostatitis) can significantly alter the balance between ROS levels and total antioxidant capacity, disrupt sperm plasma membrane fluidity, impair sperm motility and induce sperm DNA damage [3, 7].

Elevated levels of ROS are detected in 25% to 80% of infertile men [10]. Infertile men are also reported to have significantly lower reserves of antioxidants in their seminal plasma compared to their fertile counterparts [11–13]. Men with elevated ROS are also less likely to achieve a spontaneous pregnancy. Using chemiluminescence, the most common method used to measure ROS levels in the seminal ejaculates [14], we have identified that ROS levels <90 Relative Light Units per second per million spermatozoa (RLU/s/ X 10^6^ sperm) in the ejaculate are considered normal in fertile men (unpublished work). We also found that infertile men and those with unexplained or idiopathic infertility have ROS levels that are significantly higher than 90 RLU/s/10^6^ sperm (unpublished work).

Proteomics is developing rapidly as a promising field in the assessment of male infertility. Novel tools developed in the last decade have helped identify more than 6000 different sperm proteins. In addition to identification of unique sperm proteins that may contribute to male infertility, quantitative proteomics can help to determine the physiological and pathological function of these proteins with respect to their cellular localization and biological processes [15]. Differential expression of a specific protein may be used as a biomarker acting as a non-invasive diagnostic tool [16]. We have recently identified differentially expressed proteins (DEPs) in the spermatozoa and seminal plasma from subjects exhibiting high levels of ROS in the seminal ejaculates [17, 18]. Understanding the differential expression of sperm proteins is the key to understanding the cellular processes and or/pathways that may be implicated in male infertility [19].

The goal of this study is to determine the effect of increasing presence of ROS in the seminal ejaculate on the human spermatozoa proteome in male infertility patients. ROS levels in the seminal ejaculates of infertile men, categorized as low, medium and high were compared to healthy fertile men with physiological levels of ROS. To our knowledge, this is the first study that is aimed at understanding the impact of ROS levels on sperm protein alterations, biological functions and signaling pathways in infertile men.

## Methods

### Sample collection

Following approval from the Institutional Review Board of Cleveland Clinic, semen samples were collected from male infertility patients with different oxidative stress levels (n = 42) as well as healthy men of proven fertility (n =17). All subjects provided written consent to be enrolled in this prospective study.

### Inclusion/exclusion criteria

Male infertility patients 20-40 years of age who presented for infertility were enrolled in the study from March 2012 to March 2014. All female partners of the infertile men had undergone gynecologic evaluation and had normal results on fertility assessment. Patients were excluded if they had a recurring fever in the 90-day period prior to semen analysis. Patients with leukocytospermia, azoospermia and oligozoospermia were not included in the study.

### Semen analysis

All specimens were collected by masturbation at the Andrology Laboratory after 2-3 days of sexual abstinence. Samples were allowed to liquefy completely for 15-30 minutes at 37°C and manual semen analysis was performed using a MicroCell counting chamber (Vitrolife, San Diego, CA) to determine sperm concentration and motility. Smears of the raw semen were stained with a Diff-Quik kit (Baxter Healthcare Corporation, Inc., McGaw Park, IL) for assessment of sperm morphology according to WHO criteria [20]. Sample was tested for Leukoctyospermia, i.e. >1 X 10^6^ WBC/mL when the round cell concentration was >1 X 10^6^m/mL. This was confirmed by the peroxidase or the Endtz test [17].

### Reactive oxygen species (ROS) measurement

ROS formation was measured by chemiluminescence assay using luminol (5-amino-2, 3-dihydro-1, 4-phthalazinedione) as the probe. Chemiluminescence was measured for 15 min using a Berthold luminometer (Autolumat Plus 953, Oakridge, TN). Results were expressed as relative light units (RLU)/sec/X10^6^ sperm [14]. ROS concentration was divided into three categories:

- Low ROS group: ROS levels 0- < 93 RLU/sec/X 10^6^ sperm
- Medium ROS: ROS levels >93-500 RLU/sec/X 10^6^ sperm
- High ROS group: ROS concentration > 500 RLU/sec/X 10^6^

### Pooling of samples and protein extraction

Pooling of samples is common in proteomic studies. However, it is important to have equal contribution of sperm from each pool. The number is based on the amount of proteins necessary to conduct the proposed measurement of sperm concentration and running of the gel for proteomic analysis. For normalizing the sperm and protein concentration needed for proteomic analysis, a minimum total sperm concentration of about 100 X 10^6^ sperm are required to give about 1.5 mg/mL of protein. 20 μL of protein are loaded in triplicate for each run. Therefore about 75 -100 μL of protein aliquot with a protein concentration of about 1.5 mg/mL is adequate. To accomplish this, we pooled 4 samples from each group.

For proteomic analysis, we pooled 4 subjects from the fertile group and 4 subjects each from the low, medium and High ROS group in the infertile men. Equal number of spermatozoa were pooled from patients in each group and washed with PBS three times. Once the supernatant was removed, the spermatozoa were solubilized in radio-immunoprecipitation assay (RIPA) lysis buffer (Sigma-Aldrich, St. Louis, MO) containing the proteinase inhibitor cocktail (Roche, Indianapolis, IN). After complete lysis of the spermatozoa, protein concentration was determined using a bicinchoninic acid (BCA) kit (Thermo, Rockford, IL) and equal amounts of proteins was fractionated using SDS-Page 1D gel electrophoresis.

Twelve bands were cut from a single Coomassie blue stained 1D gel and analyzed in triplicate. The bands were washed, reduced, alkylated, and digested with trypsin. The completely digested extracts were eluted on LC-MS system, and the CID spectra searched against the human reference sequence database. These samples were analyzed using an LC gradient from 2 to 70% acetonitrile in 120 minutes. All of these chromatograms contained several peaks indicating efficient digestion of the samples.

### Global proteomics analysis

Samples were mixed with SDS Page buffer and separated on a 1D gel. For the protein digestion, the bands were cut to minimize excess polyacrylamide, divided into a number of smaller pieces. The gel pieces were washed with water and dehydrated in acetonitrile. The bands were then reduced with dithiothreitol (DTT) and alkylated with iodoacetamide prior to the in-gel digestion. All bands were digested in-gel using trypsin, by adding 5 μL 10 ng/μL trypsin in 50 mM ammonium bicarbonate and incubated overnight at room temperature to achieve complete digestion. The peptides that were formed were extracted from the polyacrylamide in two aliquots of 30 μL 50% acetonitrile with 5% formic acid. These extracts were combined and evaporated to <10 μL in Speedvac and then resuspended in 1% acetic acid to make up a final volume of ~30 μL for LC-MS analysis [21].

The LC-MS system was a Finnigan LTQ-Obitrap Elite hybrid mass spectrometer system. The HPLC column was a Dionex 15 cm × 75 μm internal diameter Acclaim Pepmap C18, 2 μm, 100 Å reverse phase capillary chromatography column. Five μL volumes of the extract were injected and the peptides eluted from the column by an acetonitrile/0.1% formic acid gradient at a flow rate of 0.3 μL/min were introduced into the source of the mass spectrometer on-line. The microelectrospray ion source is operated at 1.9 kV. The digest was analyzed using the data dependent multitask capability of the instrument acquiring full scan mass spectra to determine peptide molecular weights and product ion spectra to determine amino acid sequence in successive instrument scans.

### Data analysis

For semen parameters, comparison was made between fertile men and patients as well as fertile men and patients in each ROS group by Wilcoxon–rank sum test.

### Database searching

Tandem mass spectra were extracted by Proteome Discoverer version 1.4.1.288. Charge state deconvolution and de-isotoping were not performed. All MS/MS samples were analyzed using Mascot (Matrix Science, London, UK; version 2.3.02), SEQUEST (Thermo Fisher Scientific, San Jose, CA, USA; version 1.4.0.288) and X! Tandem (The GPM, thegpm.org; version CYCLONE (2010.12.01.1). Mascot, Sequest and X! Tandem were set up to search the human reference with database (33,292 entries) assuming trypsin as the digestion enzyme. These searches were performed with a fragment ion mass tolerance of 0.8 Da, and a parent ion tolerance of 10 parts per million (PPM). Carbamidomethylation of cysteine was specified as a fixed modification, and oxidation of methionine was specified as a variable modification.

### Criteria for protein identification

To validate MS/MS-based peptide and protein identifications Scaffold (version 4.0.6.1, Proteome Software Inc., Portland, OR) was used. Peptide identifications were accepted if they could be established at >95.0% probability by the Peptide Prophet algorithm [22] with Scaffold delta-mass correction. Protein identifications were accepted if they could be established at > 99.0% probability to achieve a false discovery rate (FDR) of <1.0% and contained at least 2 identified peptides. Protein probabilities were assigned by the Protein Prophet algorithm [23]. Proteins that contained similar peptides and could not be differentiated based on MS/MS analysis alone were grouped to satisfy the principles of parsimony. Proteins were annotated with gene ontology (GO) terms from National Center for Biotechnology Information (NCBI) (downloaded Oct 21, 2013) [24].

### Quantitative proteomics

For proteomic analysis, the relative quantity of the proteins was determined by comparing the number of spectra, termed spectral counts (SpCs), used to identify each protein. The total number of mass spectra (SpC) that matched peptides to a particular protein was used to measure the abundance of proteins in the complex mixture. Normalization of spectral counts using the NSAF (normalized spectral abundance factor) approach was applied prior to relative protein quantification. DEPs were obtained by applying different constraints for significance tests and/or fold-change cutoffs based on the average SpC of the protein from multiple runs.

Appropriate filters were used to identify DEPs that were dependent on the overall abundance of the proteins. It has been reported [25] that accurate quantification and determination of real biological change is dependent on the number of SpCs and hence different constraints have to be applied to SpC levels in order to circumvent the biases and maintain a constant false positive ratio (FPR) for all proteins. The abundance of the proteins was classified as High (H), Medium (M), Low (L), or Very Low (VL) based on their average spectral counts amongst the 3 replicate runs. Different constraints for significance tests (p-value) and/or fold change cutoffs (or NSAF ratio) were applied for these 4 abundance categories, as shown below:

- Very Low abundance: spectral count range 1.7-7; p ≤ 0.001 and NSAF ratio ≥ 2.5 for Upregulated, ≤ 0.4 for Downregulated proteins
- Low abundance: spectral count range 8-19; p ≤ 0.01 and NSAF ratio ≥2.5 for Upregulated, ≤ 0.4 for Downregulated proteins
- Medium abundance: spectral count range between 20-79; p ≤ 0.05 and NSAF ratio ≥ 2.0 for Upregulated, ≤ 0.5 for Downregulated proteins
- High abundance: spectral counts >80; p ≤ 0.05 and NSAF ratio ≥ 1.5 for Upregulated, ≤ 0.67 for Downregulated proteins

### Bioinformatics analysis

Functional annotation and enrichment analysis were performed using publicly available bioinformatics annotation tools and databases such as GO Term Finder [26], GO Term Mapper, UniProt, Software Tools for Researching Annotations of Proteins (STRAP) [27], Database for Annotation, Visualization and Integrated Discovery (DAVID) (http://david.niaid.nih.gov), and proprietary software package such as IPA (Ingenuity Pathway Analysis) from Ingenuity® Systems, used to obtain consensus-based, comprehensive functional context for the large list of proteins derived from proteomic study.

## Results

### Semen analysis

Semen analysis results for fertile men, infertile patient group, and the ROS (low, medium, high) groups are shown in Tables 1 and 2. Sperm concentration, morphology and ROS levels were significantly different among the fertile men and infertile men. Among the 17 fertile men, ROS levels were between 4-50 RLU (physiological levels). Of the 42 infertile men, 11 men had Low ROS levels between 0 - <93 RLU/sec/10^6^ sperm, 17 had Medium ROS group levels >93 - 500 RLU/sec/10^6^ sperm and 14 had high ROS levels >500 RLU/sec/10^6^ sperm.

**Table 1:** **Semen parameters in fertile men and infertile patients**

Parameter	Fertile men (n = 17)	Patients (n = 42)	P value ( ***t*** -test)
Age (y)	34.2 ± 7.5	34.6 ± 6.9	0.76
Volume (mL)	3.6 ± 1.4	3.2 ± 1.5	0.68
Concentration (10^6^/mL)	53.60 ± 46.98	29.77 ± 33.09	0.005
Motility (%)	47.7 ± 13.7	39.6 ± 19.2	0.13
Normal morphology (Strict criteria %)	7.7 ± 2.6	2.1 ± 1.5	<0.001
ROS (RLU/s/10^6^ sperm)*	27.7 (4.6, 45.9)	174.4 (0, 982.9)	0.024

*Values are median (25th, 75th percentile). P<0.05 was considered significant by Wilcoxon-rank sum test.

**Table 2:** **Semen parameters in fertile men and low medium and high ROS in infertile patients**

Parameter	Fertile men (n = 17)	Low ROS (n = 17)	Medium ROS (n = 11)	High ROS (n = 14)	P value ^a^	P value ^b^	P value ^c^
Age (y)	34.2 ± 7.5	34.3 ± 4.8	33.8 ± 6.8	35.7 ± 9.0	0.73	0.96	0.71
Volume (mL)	3.6 ± 1.4	2.7 ± 1.8	3.3 ± 1.4	3.8 ± 1.1	0.21	0.79	0.51
Concentration (10^6^/mL)	53.60 ± 46.98	33.49 ± 33.25	36.43 ± 32.56	20.02 ± 33.45	0.07	0.17	0.001
Motility (%)	47.7 ± 13.7	40.1 ± 23.9	45.9 ± 11.3	34.0 ± 17.1	0.41	0.94	0.006
Normal morphology (Strict criteria %)	7.7 ± 2.6	2.0 ± 1.4	1.6 ± 1.1	2.5 ± 1.8	<0.001	<0.001	<0.001
ROS (RLU/s/10^6^ sperm)*	27.7 (4.6, 45.9)	0 (0, 12.7)	189.2 (131.5, 320.1)	2003.2 (924.2, 9395)	0.02	<0.001	<0.001

^a^P value comparison between donors and low ROS patients.

^b^P value comparison between donors and medium ROS patients.

^c^P value comparison between donors and high ROS patients by Wilcoxon-rank sum test.

Among the different ROS groups, sperm concentration, motility, and morphology were significantly reduced in the high ROS group compared to the fertile group. Semen parameters in the other two ROS groups were comparable with the fertile group.

### Analysis of spermatozoa proteins

The number of proteins identified from the LC-MS/MS analysis of spermatozoa from fertile men was 1337, from patients with Low ROS was 1297, Medium ROS was 1280 and High ROS was 1331 respectively (Additional file 1: Table S2a, Additional file 2: Table S2b, Additional file 3: Table S2c, and Additional file 4: Table S2d). From the total proteins identified in each group, the number of proteins identified in:

- all three replicates were 1205 (90%) in fertile men and 1132 (87%), 1094 (85%), 1133 (85%) in the Low, Medium and High ROS groups respectively.
- two of the three replicates were 72 (5%) in fertile men and 107 (8%), 114 (9%), 125 (9%) in the Low, Medium and High ROS groups respectively.
- one of three replicates was 60 (4%) in fertile men and 61 (5%), 72 (6%), 73 (5%) in the Low, Medium and High ROS groups respectively.

Some of the more abundant proteins found in the fertile group (Additional file 1: Table S2a) as well as in the 3 ROS groups (Additional file 2: Table S2b, Additional file 3: Table S2c and Additional file 4: Table S2d) were lactotransferrin isoform 1; dynein heavy chain 8, axonemal isoform X1 and fibronectin isoform 3.

### Common proteins in low, medium and high ROS groups

**Figure 1:**
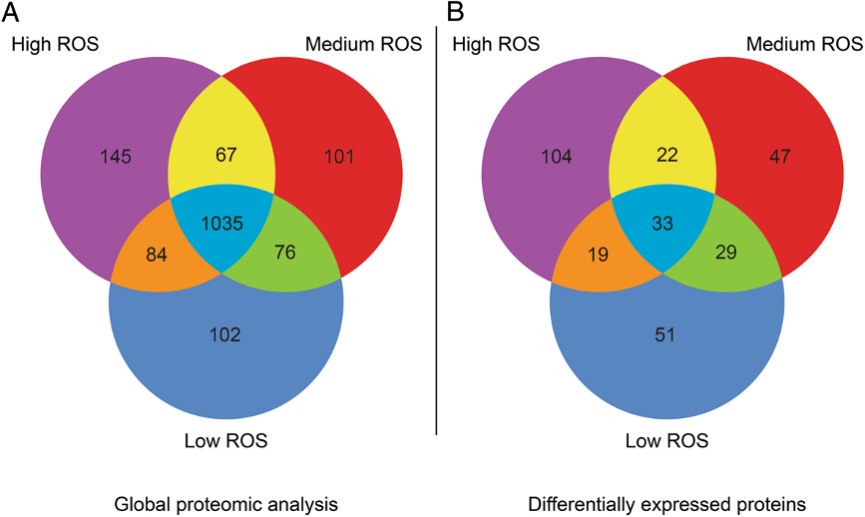
**Venn diagram showing A: global proteomic analysis and B: distribution of differentially expressed proteins in Low, Medium and High ROS group.**

A total of 1035 proteins were commonly expressed in the 3 groups. 102 proteins were only identified in the Low ROS group; 101 proteins in the medium ROS group and 145 in the High ROS group (Figure 1A).

### Differentially expressed proteins

Based on the filtering criteria described in the Methods section, the total number of DEPs obtained were 305.

**Figure 2:**
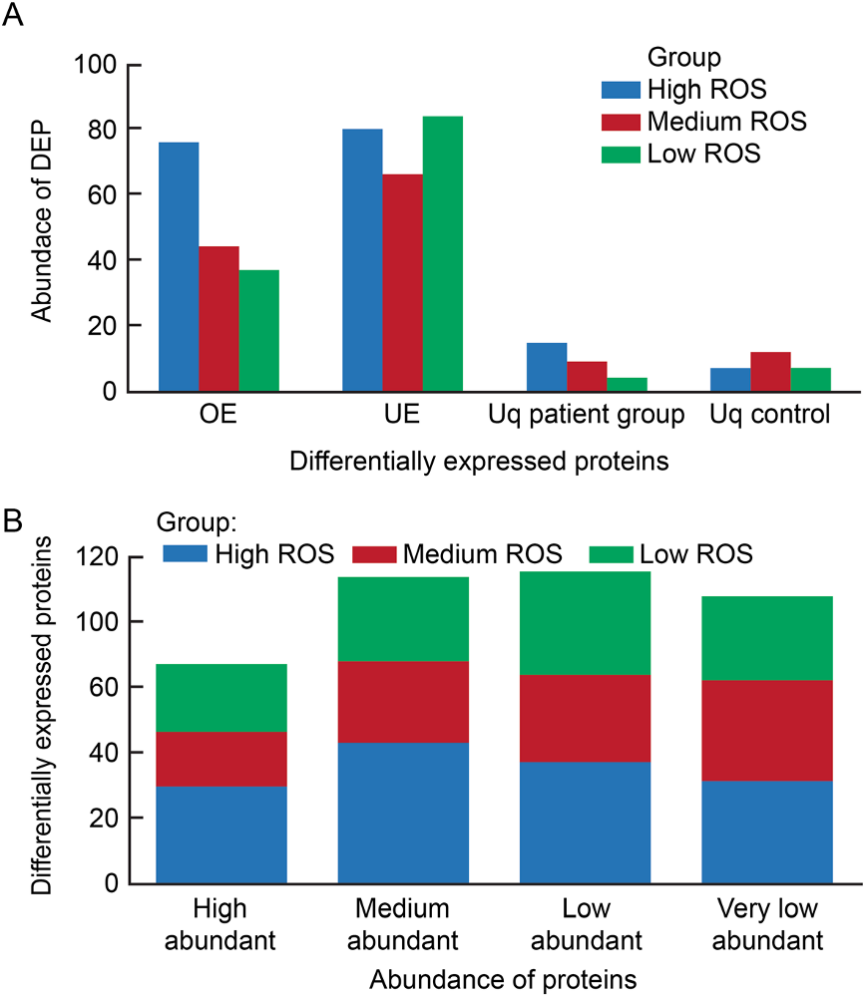
**Distribution and protein abundance in low, medium and high ROS groups A: overexpressed (OE), underexpressed (UE) and unique (Uq) B: high, medium, low and very low abundance proteins.**

Proteins that were unique, similar, or common to the 3 (Low, Medium, and High) ROS groups are shown in Figure 1B:

- 33 DEPs were identified as common to all 3 (Low, Medium and High) ROS groups when compared to the control group.
- In the Low ROS group: 132 DEPs were observed. Of these, 51 were unique, 29 were similar to Medium ROS and 19 were similar to High ROS.
- In the Medium ROS group: 131 DEPs were obtained. Of these, 47 were unique, 29 were similar to Low ROS and 22 were similar to High ROS.
- In the High ROS group: a higher number of DEPs (178) were expressed compared to the other two groups. Of the 178 DEPs, 104 were unique, 19 were similar to Low ROS, and 22 were similar to Medium ROS.
  The distribution of overexpressed (OE), underexpressed (UE), and unique proteins in each of the three categories (Low, Medium and High ROS) and the control group is shown in Figure 2A:

- Of the 132 DEPs in the Low ROS group: 37 were OE and 84 were UE respectively compared to the control group. 4 DEP were expressed only in the Low ROS group and 7 DEP were expressed only in the control group.
- Of the 131 DEPs in the Medium ROS group: 44 were OE and 66 were UE respectively compared to the control group. 9 DEP were expressed only in the Medium ROS group and 12 DEP were expressed only in the control group.
- Of the 178 DEPs in the High ROS group: 76 were OE and 80 were UE respectively compared to the control group. 15 DEP were expressed only in the High ROS group and 7 DEP were expressed only in the control group.
  The abundance of DEPs in the 3 (Low, Medium, and High) ROS groups is shown in Figure 2B.
- High Abundance proteins: 25 proteins were differentially expressed in the Low ROS group, 20 in the Medium ROS group and 36 in the High ROS group.
- Medium Abundance proteins: 31 proteins were differentially expressed in the Low ROS group, 30 in the Medium ROS group and 52 in the High ROS group.
- Low Abundance proteins: 38 differentially expressed proteins were identified in the Low ROS group, 32 in the medium ROS group and 45 in the High ROS group.
- Very Low Abundance proteins: 31 differentially expressed proteins were identified in the Low ROS group, 37 in the Medium ROS group and 38 in the High ROS group.

### Reactome pathway analysis

**Figure 3:**
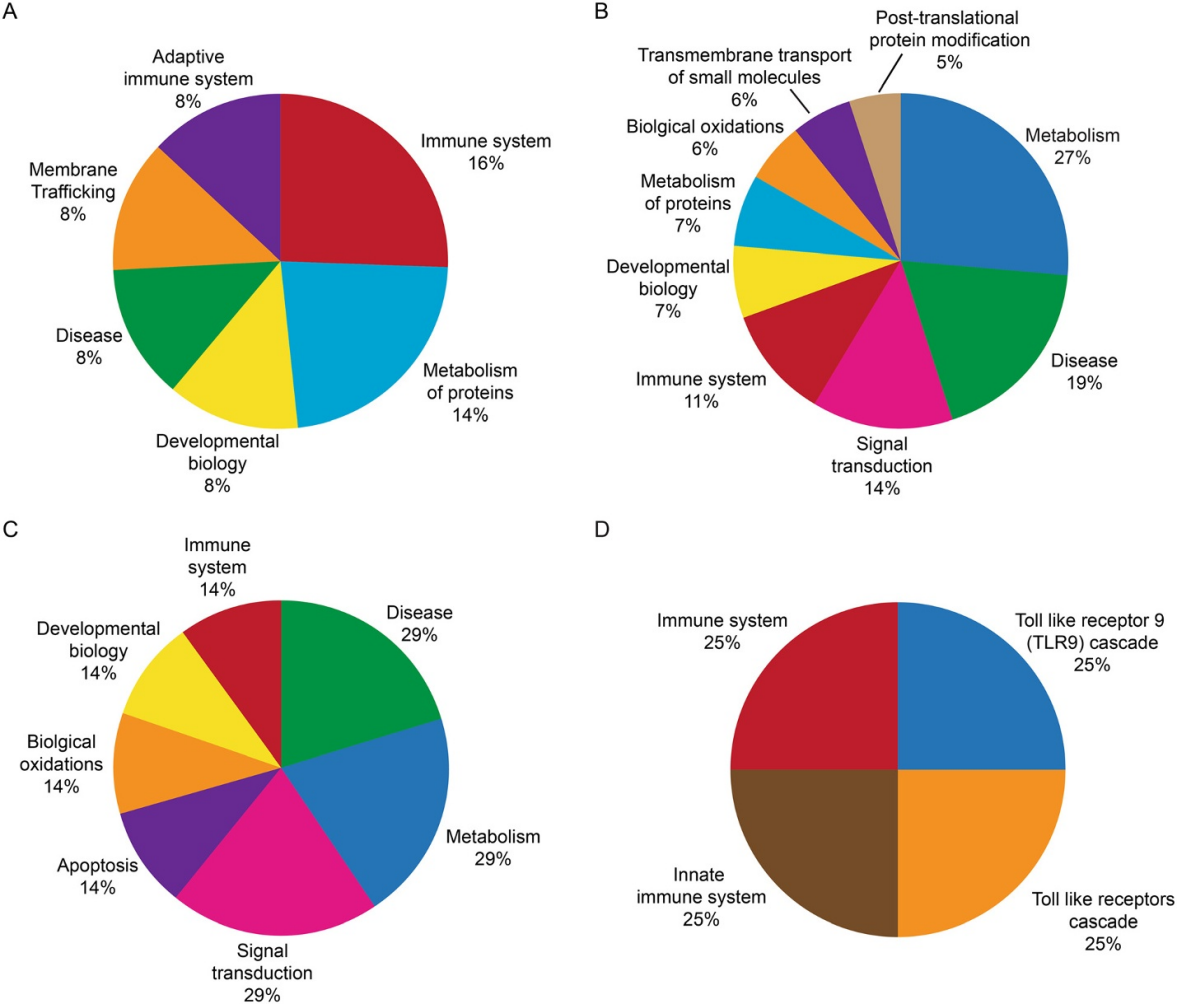
**Reactome analysis of DEPs involved in major reproductive functions in Low ROS group when compared to the fertile control group that were A: Overexpressed B: Underexpressed; C: Uniquely expressed proteins in fertile control group only and D: Uniquely expressed proteins in Low ROS group only.**

**Figure 4:**
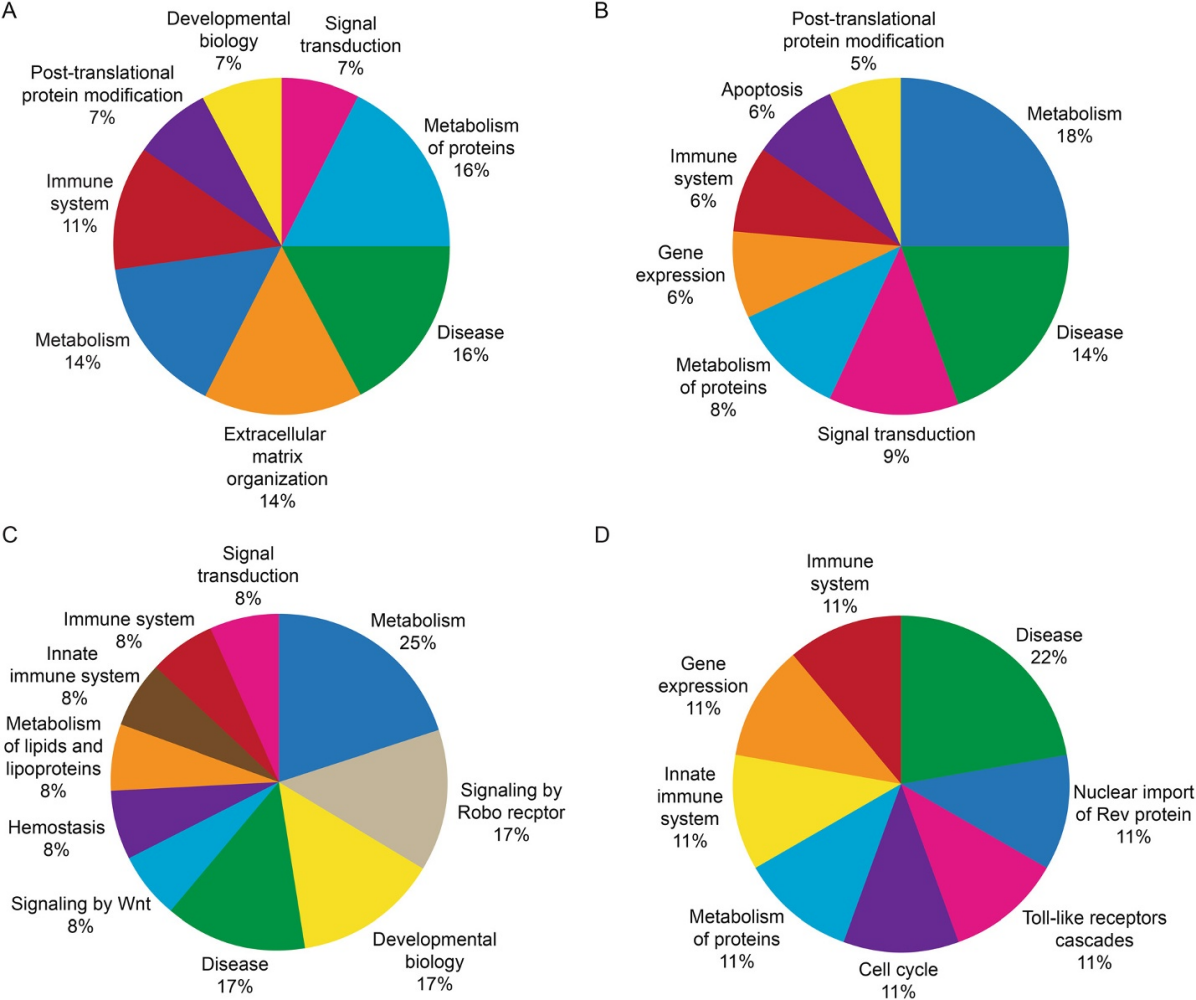
**Reactome analysis of DEPs involved in major reproductive functions in Medium ROS group when compared to the fertile control group that were A: Overexpressed B: Underexpressed; C: Uniquely expressed proteins in fertile control group only and D: Uniquely expressed proteins in Low ROS group only.**

**Figure 5:**
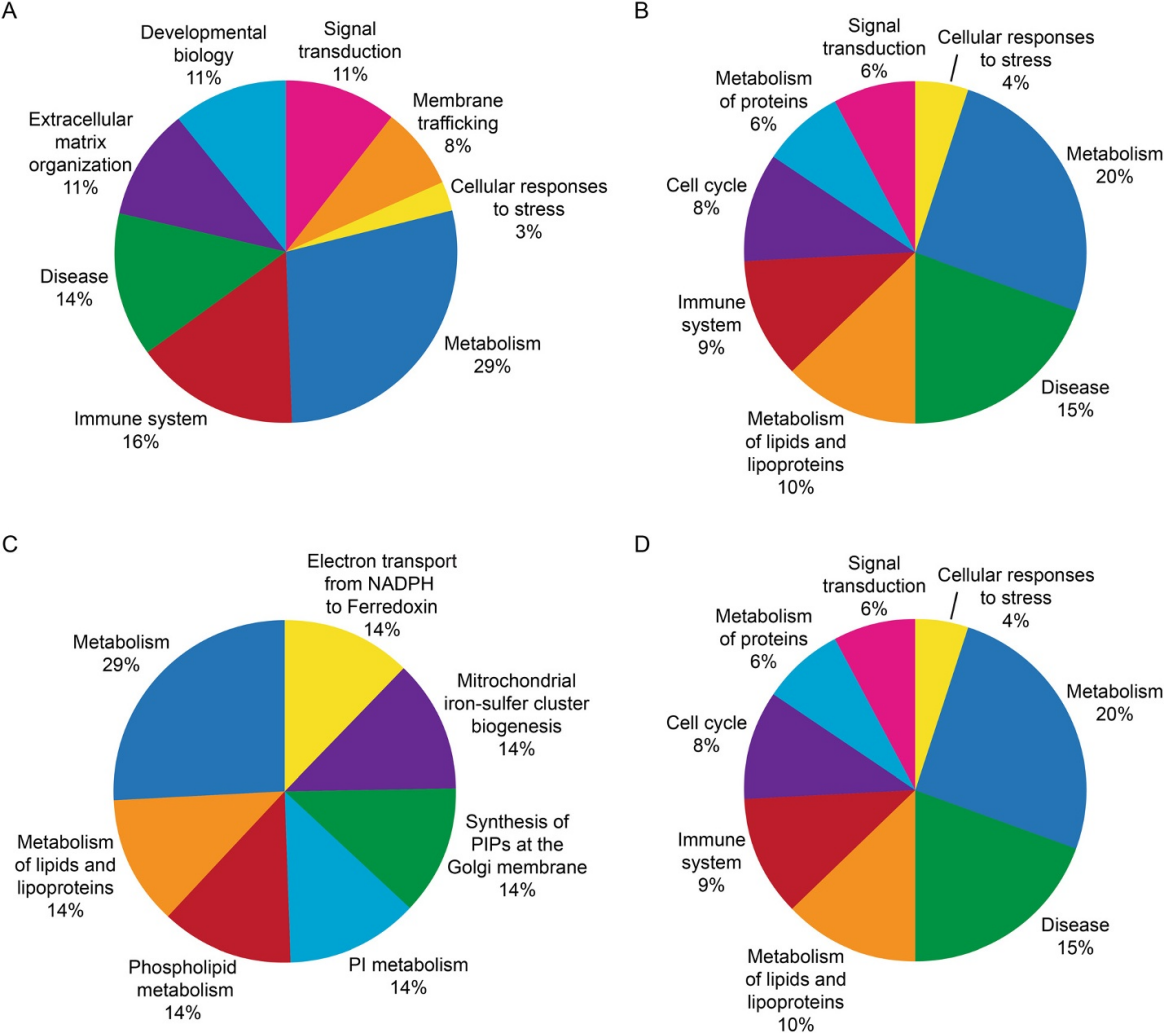
**Reactome analysis of DEPs involved in major reproductive functions in High ROS group when compared to the fertile control group that were A: Overexpressed B: Underexpressed; C: Uniquely expressed proteins in fertile control group only and D: Uniquely expressed proteins in Low ROS group only.**

From the Reactome analysis of DEPs, distribution of a select set of pathway categories (either associated with or known to affect reproductive functions) in each of the three groups is shown in Figures 3, 4 and 5.

### Low ROS versus fertile control group

- A total of 37 proteins were overexpressed in this group (Figure 3A).
- Some of the DEP were involved in the innate and adaptive immune system, metabolism of proteins, membrane trafficking, disease and developmental biology.
- The underexpressed DEP were involved in metabolism, diseases, signal transduction, and in the immune system (Figure 3B).
- Proteins that were uniquely expressed in the fertile control group only (Figure 3C) were involved in metabolism, signal transduction and disease, whereas 14% of the DEPs were involved in each category such as immune system, developmental biology, biological oxidations and apoptosis.
- Proteins that were uniquely expressed in the low ROS group shown in Figure 3D were Toll-like receptor 9, Toll-like receptor cascade, innate immune system and adaptive immune system.

### Medium ROS versus fertile control group

- 44 DEP were overexpressed in this group (Figure 4A).
- They were involved in metabolism of proteins, disease, extracellular matrix organization, metabolism, and immune system (Figure 4A).
- 7% of the proteins were involved in each category such as post-translational protein modifications, developmental biology and signal transduction (Figure 4A).
- 66 of the DEP were underexpressed in medium ROS group (Figure 4B) and were involved in metabolism, disease related pathways, signal transduction, and metabolism of proteins, and a small percentage in immune system, apoptosis and post-translational protein modifications.
- Uniquely expressed proteins in the fertile control group only that were differentially expressed were involved in metabolism, signaling by Robo receptor, developmental biology and disease as shown in Figure 4C.
- 9 DEP were uniquely expressed in the medium ROS group were involved in metabolism, while 11% of the proteins were uniquely involved in each of the categories such as toll-like receptor cascades, cell cycle, and immune system and gene expression as shown in Figure 4D.

### High ROS versus fertile control group

- 76 of the DEP were overexpressed in the High ROS group compared to the fertile control group (Figure 5A).
- The overexpressed proteins were involved in metabolism, immune system, disease, and extracellular matrix organization, developmental biology, and signal transduction, membrane trafficking and cellular responses to stress (Figure 5A).
- 80 DEPs were underexpressed and were involved in metabolism, disease, metabolism of lipids and lipoproteins, immune system, cell cycle, signal transduction, and cellular responses to stress (Figure 5B).
- 7 DEP were uniquely expressed only in the fertile control group and were involved in electron transport, mitochondrial iron-sulphur cluster biogenesis, and synthesis of PIPs at the Golgi membrane, phospholipid metabolism, metabolism of lipids and lipoproteins and general metabolism (Figure 5C).
- 15 DEP were uniquely expressed in the High ROS group (Figure 5D) and involved in metabolism, disease, metabolism of lipids and lipoproteins, immune system, cell cycle, signal transduction and cellular response to stress.

### DAVID software’s functional annotations in low, medium and high ROS group

The results of the functional annotation analysis of DEPs using DAVID software is shown in Table 3. In the Low ROS compared to the control group, 12 DEPs involved in reproduction and fertilization were under-expressed. In the Medium ROS group, 109 DEP were under-expressed. Some of the functions were related to sexual reproduction, gamete generation and reproductive process. 44 DEPs participating in reproduction, spermatogenesis, spermatid development and differentiation were under-expressed in the High ROS group.

**Table 3:** **Functional Annotations analysis of DEPs by DAVIDS analysis in low, medium and high ROS group**

Functional annotations	Low ROS	Medium ROS	High ROS
Functional categories related to reproduction/spermatogenesis	Single fertilization (4), sexual reproduction (8)	Sexual reproduction (7), gamete generation (96), reproductive process in multicellular organism (6)	Sexual reproduction (11), multicellular organism reproduction (10), gamete generation (9), spermatogenesis (8), spermatid development/differentiation(3), germ cell development (3)
Enriched functional categories	**Cell cortex (5), actin binding (5), myofibril (3), protease (4), peptidase activity (4), metal ion binding (12), protein targeting (3), IC protein transport and localization (3), cell cycle phase (3), nucleotide binding (5), regulation of transcription** (3); lysosome (7), influence lipid concentrations and risk of CAD (4), plasma lipoprotein particle remodeling (3), heparin binding (5), protein-lipid complex (3), glucose metabolic process (5), cytoskeleton organization (6), sterol transport and homeostasis (3), EGF-like region (5), cellular response to stress (3), negative regulation of apoptosis (6), cell migration (5), cell cycle (3)	**Basement membrane (6), EC matrix (6), ECM-receptor interaction (5), EGF domain (5), cell motility and localization (6), cytoplasmic vesicle (6), regulation of neurological system process (3), motor protein (3), calmodulin binding (3), metal ion binding (16) cellular ion homeostasis (3), cell morphogenesis (3), protein targeting and localization (3), synaptic transmission (3), ATP binding** (5); Dynein (6), microtubule associated complex (6), motor activity (6), negative regulation of cellular metabolic process (5), Ca binding region (4), cytoskeleton organization (5), gamete generation (6), reproductive process in multicellular organism (6), nucleotide biosynthetic process (4), nucleotide binding (12), metalloendopeptidase activity (3), glycosaminoglycan binding (3), phospholipid metabolic process (3), regulation of apoptosis (6), cytoskeleton (11), metal ion binding (12)	**Oxidoreductase (12), Protease (11), cofactor binding (8), metallopeptidase activity (7), metalloprotease (7), zinc ion binding (13), cytoskeleton (14), cell junction (6), regulation of apoptosis (7), intracellular protein transport and localization (5), proteasome complex (3), vasculature development (4), focal adhesion (4);** nucleotide binding (24), sexual reproduction (11), multicellular organism reproduction (10), gamete generation (9), spermatogenesis (8), spermatid development/differentiation(3), germ cell development (3), glutathione S-transferase (3), peroxisome (4), fatty acid oxidation (3), lipid modification (3), glycerolipid metabolic process (4), protein targeting (3), metal ion binding (13), apoptosis (3)
Majority of proteins associated with functions	**Polymorphism (29), phosphoprotein (22), acetylation (15), glycoprotein (13), Golgi (9);** polymorphism (62), signal peptide (38), glycoprotein (36), disulfide bond (34), extracellular (30), acetylation (26), secreted (23), oxidoreductase (11), lipid binding (11)	**Phosphoprotein (26), glycoprotein (20), signal peptide (18), disulfide bond (15), acetylation (13), cytoplasm (13), Golgi (10), EC region (11), secreted (10), Ca ion binding (8), cell adhesion (7);** Polymorphism (50), Signal peptide (18), EC (17), secreted (15), mitochondrion (13), nucleotide binding (15), cell fraction (10), cytoskeleton (9), sexual reproduction (7)	**Polymorphism (57), phosphoprotein (41), ion binding (34), glycoprotein (27), cytoplasm (26), signal peptide (22), cell membrane (16), proteolysis (14), hydrolase (15), oxidoreductase (12), defense response (9), metalloprotease (7), protein transport and localization (8), exopeptidase activity (6), adherens junction (6), cell migration (6);** purine nucleotide binding (24), ribonucleotide binding (22), sexual reproduction (11), gamete generation (9), spermatogenesis (8), mitochondrion (15), reproductive cellular process (5), protein folding (5), spermatid development (3)
Cellular distribution	**Cell cortex (5), Golgi (9), cytoskeleton (9), cytosol (8), ER (6), organelle envelope (5), vesicle (5), sarcomere (3);** extracellular region (30), cytosol (13), lysosome (7), vesicle (11), secretory granule (4), protein-lipid complex (3), cell surface (6), golgi (10)	**Golgi (10), EC region (9), organelle membrane (9), ER (8), proteinaceous ECM (6), cytoplasmic vesicle (6), intrinsic to plasma membrane (8);** dynein complex (6), mitochondrion (13), EC region (17), microtubule cytoskeleton (8), cell projection (8), cytoskeleton (11), cilium (4)	**Actin cytoskeleton (10), cell cortex (7), cortical cytoskeleton (5), cell-cell adherens junction (4), vesicle (11), Golgi (12), EC region (19), mitochondrial lumen (4);** mitochondrion (15), cytosol (15), dynein complex (3), microbody (4), peroxisome (4), cell fraction (11), secretory granule (4), organelle envelope (7), pore complex (3)
Activated processes/functions	**Intracellular transport (7), protein localization (7), cytoskeletal organization (5), regulation of protein complex assembly (3), microtubule based process (4), calmodulin binding (4), motor activity (4), actin binding (5), serine hydrolase activity (3), phospholipid binding (3)**	**Cell motility and localization (6), cellular iron ion hemostasis (3), cell migration (5), cell motion (6), cell adhesion (7), integrin-mediated signaling pathway (3), macromolecular complex assembly (6), response to wounding (5), regulation of neurological system process (3), Ca ion binding (8), peptidase activity (5), carbohydrate binding (4), microfilament motor activity (2), structural molecule activity (5)**	**Actin filament based process (9), oxidation reduction (12), integrin mediated signaling pathway (5), cytoskeleton organization (9), proteolysis (14), cell motion (9), defense response (9), intracellular transport (8), cell-cell adhesion (5), oxygen and ROS metabolic process (3), actin binding (10), exo- or endo-peptidase activity (6), cytoskeletal protein binding (11), calcium ion binding (11), cofactor binding (8), peptidase activity (11), antioxidant activity (3)**
Downregulated processes/functions	Glycerolipid metabolic process (6), lipid hemostasis (4), oxidation reduction (11), single fertilization (4), protein-lipid complex remodeling (3), sexual reproduction (8), protein folding (5), sterol transport (3), response to oxidative stress (5), lipid binding (11), carbohydrate binding (8), glycosaminoglycan binding (5), oxidoreductase activity (3), antioxidase activity (3)	Actin filament organization (4), protein folding (5), negative regulation of cellular metabolic process (5), sexual reproduction (7), protein tetramerization (3), oxidation reduction (8), gamete generation (6), cell redox homeostasis (3), protein oligomerization (4), generation of precursor metabolites and energy (5); motor activity (6), enzyme inhibitor activity (6), proteasome regulator activity 92), protein binding (7), nucleoside binding (12), nucleotide binding (15), protein homodimerization activity (5)	Sexual reproduction (11), reproductive process in a multicellular organism (10), gamete generation (9), spermatogenesis (8), protein folding 95), glycerophospholipid metabolic process (4), spermatid development/differentiation (3); purine nucleotide binding (24), ATP binding (18), microtubule motor activity (3), sterol transporter activity (2), cAMP dependent protein kinase regulator activity (2), glutathione transferase activity (2)
Activated pathways	**Ether lipid metabolism (2), Synaptic proteins at the synaptic junction (2);**	**ECM-receptor interaction (5), Integrin cell surface interactions (4)**	**Leukocyte transendothelial migration (5), focal adhesion (8), ECM-receptor interaction (4), Adherens junction (4), Hemostasis (7), Apoptosis (5), Metabolism of lipids and lipoproteins (5), metabolism of amino acids (5), Wntsignaling (3), Integrin signaling pathway (3), Tight junction (4), Proteasome (3)**
Downregulated pathways	Gluconeogenesis (3), metabolism of carbohydrates (4), hemostasis (5)	Fructose and mannose metabolism (3), apoptosis (4), metabolism of amino acids (4)	Glycolysis/gluconeogenesis (3), adipocytokine signaling pathway (3), PPAR signaling pathway (3), metabolism of lipids and lipoproteins (5), Integration of energy metabolism (5), mechanism of protein import into the nucleus (2)
**Processes/Functions unique to each ROS group**	*Regulation of cellular protein metabolic process (2), endocytosis (1), PTM (1)*	*Organelle fusion (2), protein homodimerization activity (2), anti-apoptosis (2), ribosomal subunit (2), cytosol (3), membrane (4), polymorphism (5)*	*Disulfide bond (7), signal peptide (6), alternative splicing (8), metal ion binding (7), glycoprotein (8), secreted (4), endocytosis (2), aging (2), plasma membrane (5), calcium ion binding (5)*
**Processes/Functions unique to Control group**	*MAPK signaling pathway (2), Regulation of actin cytoskeleton (2), metabolism of vitamins and cofactors (2)*	*Tissue morphogenesis (3), tube development (3), embryonic morphogenesis (3), leucine rich repeat (3), extracellular region (5), glycoprotein (5), membrane (3), urogenital system development (2)*	*Transmembrane (3), alternative splicing (3), transport (4), metabolism of vitamins and cofactors (1)*

Bold text = Overexpressed proteins; regular text = Underexpressed proteins; italics text = unique processes or functions; number in parenthesis = number of proteins.

The other enriched functional categories, cellular function and distribution, as well as processes and functions that were either activated or suppressed in each of the three groups are all summarized in Table 3. The number of proteins associated with each category is shown in parentheses. The fertile control group as well as in each ROS group is also summarized in Table 3.

### STRAP analysis of DEP in low, medium and high ROS group

STRAP analysis identified 6 DEPs (CLGN, TPP2, DNAI2, HSPA4L, EEA1 and SERPINA5) associated with key reproductive related functions. The differential expression of these proteins in the three ROS groups is shown in Table 4.

**Table 4:** **Differentially expressed proteins as a potential biomarker for patients exhibiting high, medium and low ROS levels**

Uniprot number	Protein name	Gene name	Function	Expression/ROS level	Possible reasons for protein expression and relation to infertility	Reference
O14967	Calmegin	CLGN	Essential for formation of normal spermatozoa. Important role in spermatogenesis, acts as a chaperone for a range of client proteins that are important for sperm adhesion onto the egg zona pellucida and for subsequent penetration of the zona pellucida.	Overexpressed/ High ROS group, Medium ROS group, Low ROS group	Its expression is triggered in case of elevated oxidative stress. Thus, in men with ROS generation above physiological levels (oxidative stress), calmegin overexpression may impair the ability of spermatozoa to bind to the zona pellucida. Hence, it may be a cause of infertility in these men.	[28–30]
P29144	Tripeptidyl-peptidase 2	TPPII	Tripeptidyl peptidase II is a ‘multi-purpose peptidase’ with house-keeping function in intracellular protein degradation and plays a role in several vital cellular processes such as cell division, apoptosis or antigen processing. TPPII regulates sperm function by modifying the levels of tyrosine phosphorylation. It is involved in the fertilization process, and regulates sperm maturation.	Overexpressed/ High ROS group, Medium ROS group, Low ROS group	Overexpression of TPPII in men with ROS generation above physiological levels may modify sperm protein tyrosine phosphorylation levels, such that spermatozoa is unable to undergo protein tyrosine phosphorylation-associated processes such as capacitation, hyperactivation, and acrosome reaction.	[31–33]
Q9GZS0	Dynein intermediate chain 2, axonemal	DNAI2	Part of the dynein complex of respiratory cilia. DNAI2 can result in reduced fertility due to sperm tail abnormalities.	Underexpressed/High ROS group, Medium ROS group, Low ROS group	The underexpression of DHAI2 in men with ROS above physiological levels may contribute to the negative effect of oxidative stress on sperm motility.	[34]
Q15075	Early endosome antigen 1	EEA1, ZFYVE2	Binds phospholipid vesicles containing phosphatidylinositol 3-phosphate and participates in endosomal trafficking.	Uniquely expressed/High ROS group, Medium ROS group, Low ROS group	Its unique expression in the 3 ROS groups suggests that EEA1 may be involved in the failure of acrosome biogenesis, that results in male infertility.	[35–37]
O95757	Heat shock 70 kDa protein 4L	HSPA4L, APG1, OSP94	Apg-1 encodes a heat shock protein belonging to the Hsp110 family and is inducible by a 32 degrees C to 39 degrees C heat shock in somatic cells. In mouse testicular germ cells Apg-1 mRNA is constitutively expressed depending on the developmental stage. It belongs to the HSP110 heat shock gene family and is produced ubiquitously and predominantly in the testis. It is highly expressed in the spermatogenic cells, from late pachytene spermatocytes to post meiotic spermatids. It is required for normal spermatogenesis.	Underexpressed/High ROS group, Medium ROS group, Low ROS group	The underexpression of HSPA4L in men with ROS above physiological levels may disrupt the normal spermatogenesis process which may contribute to infertility seen in these men.	[38]
P05154	Plasma serine protease inhibitor	SERPINA5, PCI, PLANH3, PROCI	SERPINA5 is a heparin-dependent serine protease inhibitor that acts on body fluids and secretions. Serine protease with lys and arg ester bond specificity is involved in the control of sperm motility. SERPINA5 inhibits the serpin acrosin and indirectly protects the component of the male genital tract from being degraded by excessive released acrosin. It also inhibits tissue-and urinary-type plasminogen activator, prostate-specific antigen and kallikrein activities and has a control on sperm motility and fertilization.	Underexpressed/High ROS group, Medium ROS group, Low ROS group	Underexpression of SERPINA5 in men with ROS generation above physiological levels suggests that the reduced motility seen in these infertile patients may be due to the serine protease inhibiting action of SERPINA 5.	[39–41]

## Discussion

Male infertility is a multifactorial condition emerging from a wide variety of etiologies including gene mutations, malformation of reproductive organs, infectious disease, or environmental exposure to toxicants [42]. In the present study, the age of men with oxidative stress was comparable to that of fertile donors. The majority of the patients (73.2%) presented with either Low ROS, Medium ROS, or High ROS. Semen analysis was performed according to the new WHO 2010 criteria [20]. No significant differences were observed in sperm concentration, motility or presence of round cells between men presenting with Low ROS, Medium ROS and High ROS. Poor sperm morphology was seen in men in the high ROS group when compared to Medium ROS and Low ROS group.

Some abnormal sperm morphologies are genetically- determined while others are caused as a result of repeated physiological and environmental stresses. These abnormal morphologies are reversible; however, after repeated stress attacks, the testis may fail to fully recuperate and could result in a permanent decrease in number of sperm with normal morphology. These abnormalities can be explained by the effect of elevated level of oxidative stress in high ROS group. We have recently demonstrated the association of teratozoospermia with increased levels of ROS [43]. Basic semen analysis is not adequate to reflect all the parameters of semen quality and function that are required for an optimum fertility status especially in cases of idiopathic male factor infertility. Mature spermatozoa are particularly susceptible to ROS damage because of the polyunsaturated nature of the sperm plasma membrane and the absence of any transcriptional activity [3, 6].

ROS has been shown to be an independent marker of oxidative stress. ROS levels can predict male factor infertility (MFI) with an accuracy of ≥80% [30]. ROS-induced oxidative stress has been shown to be a significant risk factor associated with male factor infertility [11]. We postulated the mechanisms for infertility among non-leukocytospermic patients with normal semen parameters and high or normal ROS levels. These were (1) direct generation of oxygen radicals by low concentrations of leukocytes; (2) significant DNA damage in samples containing abnormal levels of ROS-producing spermatozoa; (3) diminished levels of antioxidants in the seminal plasma; or (4) the presence of immature sperm in substantial quantities in semen [3, 44, 45].

High ROS levels are known to significantly affect proteins and lipids, induce apoptosis and cause DNA damage, leading to male infertility [46]. Cells that are in a state of oxidative stress are more likely to have altered protein expression. Utilizing novel proteomic tools and bioinformatics we have recently demonstrated the effect of increased ROS levels on protein alterations seen in the spermatozoa and seminal plasma of subjects demonstrating high ROS levels when compared to those with physiological levels [17, 18].

The proteome provides a dynamic understanding of post-genomic events and characterizing these events will further our understanding of the underlying functional changes in men diagnosed with various male-infertility-associated etiologies [47, 48]. Current literature suggests that while causes of elevated oxidative stress levels are multifactorial, current management of oxidative stress is unable to accurately predict the benefit for male infertility.

In the present study, the Reactome software showed that DEPs in Low, Medium or High ROS group were involved in the metabolism of proteins, immune system, disease, transmembrane (TM) transport, extracellular matrix organization, signal transduction, post-translational modifications, and cellular response to stress. (Figures 4 and 5). DAVID analysis indicated that DEPs were involved in the cellular functions of lipid metabolism, small molecule biochemistry, and nucleic acid metabolism (Table 3). The overexpression of these proteins increased with increasing ROS levels indicating an overactivity of protein function in response to stress. In an excellent review by Amaral et al. [19], the authors demonstrated that the pathways involved in metabolism and energy production have been recognized as the most significant cellular pathway in the human spermatozoa. These authors further showed that 26% of the proteins contributing to the reactome belong to the Metabolism and energy production group. The contribution by the metabolism and energy groups comprised of the following: Glycolysis and gluconeogenesis (8%), Krebs cycle (13%), Mitochondrial electron transport chain/oxidative phosphorylation (20%), lipid metabolism (24%), amino acid metabolism (9%), nucleotide metabolism (7%) and other carbohydrate pathways (9%). Human sperm can utilize carbohydrate, lipid as well as nucleotide metabolism as an energy source. An increasing body of data suggests that mitochondrial activity is intimately related to sperm function such as sperm motility and fertilization. The sperm proteome is also enriched in proteins related to protein metabolism which include pathways implicated in protein translation, folding, post-translational modifications and protein degradation. The overexpressed proteins were seen in all ROS groups with varying distribution of the cellular pathways as shown by the reactome analysis.

The role of membrane trafficking is well known in spermiogenesis. The sperm proteome is enriched in proteins that mediate membrane fusion and promotes the release of acrosomal contents. The proteins involved in the final sperm-oocyte fusion event are also present in the sperm proteome. Many of the overexpressed proteins are also involved in signal transduction, extracellular matrix organization and cellular response to stress.

Many of the DEP that were underexpressed in the 3 ROS groups were also involved in the important pathways such as metabolism of proteins and lipids, signal transduction, biological oxidations, post-translational protein modifications, metabolism of proteins, transmembrane transport of small molecules, signal transduction, apoptosis, and cellular response to stress.

Compared to the Low ROS group, there were proteins that were uniquely expressed in the fertile control group. These were involved in disease, apoptosis, biological oxidation, signal transduction and immune system. In the medium ROS group, the proteins that were uniquely expressed in the fertile group were involved in events such as metabolism, metabolism of lipids and lipoproteins, signaling by Wnt and by Robo receptor, hemostasis and signal transduction. Compared to the High ROS group, DEP that were uniquely expressed in the fertile group were involved in metabolism, electron transport from NADPH to ferrodoxin, synthesis of phosphatidylinositol phosphate (PIP) at the Golgi membrane, phospholipid metabolism, mitochondrial iron-sulfur cluster biogenesis, metabolism of lipids and lipoproteins as well as phosphoionositide (PI) metabolism that play a role in lipid signaling, cell signaling and membrane trafficking.

The Reactome analysis also identified proteins that were uniquely expressed in each ROS group. Proteins that were unique to the Low ROS group were involved in Toll-like Receptor 9 (TLR9) cascade, toll-like receptor cascades, and innate immune system. These receptors mediate response to unmethylated CpG dinucleotides in bacterial DNA to trigger an immune response. The presence of these receptors may be indicative of leukocyte contamination in ROS generating semen samples. Proteins that were uniquely expressed in the Medium ROS group were involved in disease, gene expression, metabolism of proteins, toll-like receptors cascades, nuclear import of Rev proteins and both innate and adaptive immune system. Innate immunity is an inherent immunity (defense) that is already present at birth.

Similarly in the High ROS group, a significantly high percentage of uniquely expressed proteins were involved in metabolism, electron transport from NADPH to ferredoxin, and metabolism of the phospholipids, lipids, lipoprotein synthesis of PIP at the Golgi membrane suggesting that these proteins were playing a key role in optimal spermatozoa function. On the other hand, in the patients within the High ROS group, proteins were unique and involved in cellular response to stress, signal transduction, metabolism of proteins, metabolism of lipids and lipoproteins suggesting that these proteins were operating under high oxidative stress conditions that was affecting the metabolic activity of the sperm and in signal transduction and response to stress.

The most significant pathways in the male gamete are those involved in protein metabolism, membrane trafficking, apoptosis, cell cycle, hemostasis and meiosis. Developmental biology and extracellular matrix organization were also detected as putative sperm pathways but with lower probability. Signaling transduction is not a major pathway but some signaling pathways that are seen to be active include signaling by Wnt proteins, a family of secreted lipid-modified signaling glycoproteins [19]. The overexpression/under expression or the unique presence of the proteins in the ROS groups only compared to the fertile group is indicative of the problems in the sperm functions that are affected or altered as a result of increased presence of ROS in these men. These alterations may be responsible for poor fertility seen in these men compared to the fertile men.

We also demonstrated that while oxidative phosphorylation is important for sperm function, the major pathway for energy metabolism was the glycolytic pathway. Majority of the proteins were involved in cellular metabolic and regulatory processes. We demonstrated in our earlier work that a number of proteins that were overexpressed or underexpressed in ROS+ group were involved in cellular processes, metabolic process, response to stress and transport. Furthermore, a small number of proteins were involved in post-translational processes and protein folding [18].

DAVID software also identified a number of proteins that were involved in key functions in the 3 ROS groups. In the functional categories related to reproduction and/or spermatogenic events, 12 proteins were identified in the low ROS group, 109 in the medium ROS and 44 in the high ROS group that may be modified and were underexpressed compared to the fertile group. The various functional annotations are shown in Table 3.

The majority of the overexpressed proteins were associated with various functions such as acetylation, phosphoproteins, Ca^2+^ ion binding, oxidoreductase, defense response, protein transport, etc. whereas other proteins involved in signal peptide, nucleotide, binding, gamete generation, spermatogenesis, protein folding and spermatid development were underexpressed especially in the high ROS group. This is the category with the largest distribution of proteins that were differentially expressed highlighting the deviation of the various functions from the fertile group.

We further identified the DEPs in all 3 ROS groups that are known to play a role in reproduction and spermatogenesis that were underexpressed, over or were uniquely expressed to each of the 3 ROS groups compared to the fertile controls (Table 4). These proteins highlight the deviation from the normal functions that are seen in fertile men in the presence of physiological levels of ROS. We identified 6 DEP (Calmegin, Tripeptidyl peptidase II, Dynein intermediate chain 2, axonemal, Heat shock 70 kDa protein 4L, Early endosome antigen 1, and Plasma serine protease inhibitor) that were present in all the 3 ROS groups with varying expression levels and therefore may serve as potential candidates of oxidative stress (Table 4).

In conclusion, we have for the first time demonstrated poor sperm quality that is associated with elevated oxidative stress level by categorizing the patient semen samples into low, medium and high ROS groups. These abnormalities can be explained by the altered expression of specifically DEPs Calmegin, tripeptidyl synthesase, dynein intermediate chain 2, heat shock 70 kDa protein 4L, early endosome antigen 1, plasma serine protease inhibitor in patient groups. These DEPs may serve as potential biomarkers in the identification of the effect of increasing ROS level on protein profile of spermatozoa in infertile men. Further validation of DEPs is necessary to establish the role of these proteins as biomarkers of oxidative stress induced male factor infertility.

## Electronic supplementary material

Additional file 1: Table S2a: Spermatozoa proteins in fertile men. (DOCX 308 KB)

Additional file 2: Table S2b: Spermatozoa proteins in Low ROS group. (DOCX 305 KB)

Additional file 3: Table S2c: Spermatozoa proteins in Medium ROS group. (DOCX 294 KB)

Additional file 4: Table S2d: Spermatozoa proteins in High ROS group. (DOCX 301 KB)
