# Supplementary material for: Impact of precise modulation of reactive oxygen species levels on spermatozoa proteins in infertile men
Source: Clin Proteomics. 2015 Feb 9;12(1):4. doi: 10.1186/1559-0275-12-4 (PMC4429661; doi:10.1186/1559-0275-12-4)
Supplement: Supplementary file 1 — Additional file 1: Table S2a: Spermatozoa proteins in fertile men. (DOCX 308 KB) [file 12014_2014_99_MOESM1_ESM.docx]

| **Table S2a. Spermatozoa proteins in fertile men.** | |  |  |  |  |  |  |  |  |  |  |
| --- | --- | --- | --- | --- | --- | --- | --- | --- | --- | --- | --- |
|  |  |  |  |  |  |  |  |  |  |  |  |
| Protein | accession | MW |  | Fertile group | | Fertile group | | | Fertile group | | |
|  |  | kDa |  | Replicate 1 | |  | Replicate 2 | |  | Replicate 3 | |
| lactotransferrin isoform 1 precursor | 54607120 | 78 | 121 | 85% | 3855 | 124 | 82% | 2799 | 122 | 82% | 3404 |
| dynein heavy chain 8, axonemal isoform X1 | 578811443 | 539 | 92 | 25% | 230 | 100 | 25% | 314 | 101 | 26% | 287 |
| fibronectin isoform 3 preproprotein | 16933542 | 259 | 78 | 39% | 392 | 85 | 46% | 504 | 81 | 44% | 474 |
| aminopeptidase N isoform X1 | 530407092 | 110 | 63 | 56% | 508 | 58 | 56% | 514 | 59 | 56% | 525 |
| heat shock protein HSP 90-alpha isoform 1 | 153792590 | 98 | 59 | 54% | 687 | 60 | 57% | 657 | 57 | 56% | 701 |
| endoplasmin precursor | 4507677 | 92 | 58 | 63% | 795 | 57 | 63% | 697 | 54 | 62% | 878 |
| UDP-glucose:glycoprotein glucosyltransferase 1 isoform X3 | 578804605 | 176 | 51 | 46% | 179 | 60 | 52% | 260 | 58 | 49% | 219 |
| angiotensin-converting enzyme isoform 1 precursor | 4503273 | 150 | 50 | 42% | 427 | 56 | 43% | 506 | 55 | 41% | 485 |
| semenogelin-2 precursor | 4506885 | 65 | 50 | 52% | 528 | 45 | 54% | 631 | 52 | 52% | 635 |
| trifunctional enzyme subunit alpha, mitochondrial precursor | 20127408 | 83 | 49 | 73% | 1041 | 54 | 74% | 877 | 48 | 72% | 957 |
| aconitate hydratase, mitochondrial precursor | 4501867 | 85 | 48 | 70% | 450 | 50 | 65% | 444 | 49 | 68% | 459 |
| hexokinase-1 isoform X2 | 530393498 | 103 | 48 | 52% | 400 | 46 | 48% | 384 | 47 | 50% | 355 |
| serum albumin preproprotein | 4502027 | 69 | 46 | 77% | 496 | 45 | 73% | 466 | 45 | 72% | 450 |
| 60 heat shock protein, mitochondrial isoform X1 | 530370277 | 61 | 46 | 75% | 525 | 43 | 77% | 487 | 43 | 75% | 499 |
| tripeptidyl-peptidase 2 isoform X2 | 530423399 | 143 | 45 | 41% | 198 | 45 | 42% | 213 | 47 | 45% | 244 |
| 78 glucose-regulated protein precursor | 16507237 | 72 | 45 | 69% | 611 | 44 | 65% | 561 | 46 | 65% | 612 |
| ATP synthase subunit alpha, mitochondrial isoform a precursor | 50345984 | 60 | 41 | 67% | 606 | 33 | 61% | 584 | 36 | 62% | 563 |
| laminin subunit beta-2 isoform X1 | 530372442 | 196 | 39 | 35% | 110 | 47 | 41% | 155 | 40 | 34% | 132 |
| ATP synthase subunit beta, mitochondrial precursor | 32189394 | 57 | 39 | 80% | 887 | 39 | 78% | 810 | 41 | 78% | 889 |
| myoferlin isoform b | 19718759 | 233 | 38 | 15% | 96 | 38 | 22% | 99 | 30 | 15% | 83 |
| heat shock-related 70 protein 2 | 13676857 | 70 | 38 | 69% | 477 | 37 | 64% | 462 | 36 | 67% | 489 |
| hypoxia up-regulated protein 1 precursor | 5453832 | 111 | 37 | 43% | 288 | 45 | 47% | 311 | 43 | 47% | 324 |
| transitional endoplasmic reticulum ATPase | 6005942 | 89 | 37 | 67% | 257 | 39 | 63% | 235 | 37 | 62% | 253 |
| plastin-2 isoform X2 | 530402335 | 70 | 37 | 76% | 194 | 36 | 76% | 174 | 38 | 75% | 192 |
| carnitine O-palmitoyltransferase 2, mitochondrial precursor | 4503023 | 74 | 37 | 68% | 212 | 36 | 69% | 231 | 36 | 67% | 235 |
| protein disulfide-isomerase A3 precursor | 21361657 | 57 | 36 | 64% | 317 | 36 | 66% | 326 | 42 | 63% | 363 |
| neutral alpha-glucosidase AB isoform 3 precursor | 88900491 | 109 | 35 | 52% | 175 | 34 | 55% | 168 | 40 | 62% | 211 |
| trifunctional enzyme subunit beta, mitochondrial isoform 1 precursor | 4504327 | 51 | 35 | 72% | 413 | 34 | 69% | 375 | 33 | 69% | 464 |
| sodium/potassium-transporting ATPase subunit alpha-3 isoform 1 | 22748667 | 112 | 35 | 42% | 135 | 32 | 41% | 131 | 37 | 45% | 156 |
| plasma membrane calcium-transporting ATPase 4 isoform 4b | 48255957 | 134 | 33 | 31% | 129 | 40 | 38% | 160 | 40 | 37% | 161 |
| NAD-dependent malic enzyme, mitochondrial isoform 1 precursor | 4505145 | 65 | 33 | 75% | 189 | 31 | 74% | 184 | 29 | 71% | 200 |
| T-complex protein 1 subunit eta isoform a | 5453607 | 59 | 33 | 74% | 252 | 29 | 72% | 228 | 32 | 74% | 272 |
| laminin subunit gamma-1 precursor | 145309326 | 178 | 32 | 31% | 98 | 42 | 42% | 131 | 39 | 37% | 118 |
| L-lactate dehydrogenase C chain | 9257228 | 36 | 32 | 84% | 509 | 35 | 80% | 454 | 33 | 79% | 534 |
| long-chain-fatty-acid--CoA ligase 6 isoform e | 327412327 | 79 | 32 | 49% | 136 | 33 | 52% | 137 | 32 | 51% | 151 |
| A-kinase anchor protein 4 isoform 2 | 21493039 | 93 | 31 | 40% | 223 | 33 | 45% | 289 | 36 | 44% | 267 |
| semenogelin-1 preproprotein | 4506883 | 52 | 31 | 63% | 304 | 32 | 59% | 380 | 33 | 61% | 347 |
| NADH-ubiquinone oxidoreductase 75 subunit, mitochondrial isoform 5 | 316983160 | 81 | 31 | 59% | 144 | 32 | 59% | 130 | 33 | 59% | 150 |
| actin, cytoplasmic 2 | 316659409 | 42 | 31 | 84% | 482 | 32 | 82% | 418 | 31 | 84% | 439 |
| acetyl-CoA acetyltransferase, mitochondrial precursor | 4557237 | 45 | 31 | 67% | 390 | 30 | 73% | 376 | 31 | 65% | 402 |
| cytosol aminopeptidase | 41393561 | 56 | 31 | 78% | 329 | 29 | 73% | 320 | 31 | 77% | 353 |
| T-complex protein 1 subunit theta isoform 1 | 48762932 | 60 | 31 | 64% | 194 | 28 | 57% | 162 | 29 | 55% | 175 |
| succinate dehydrogenase [ubiquinone] flavoprotein subunit, mitochondrial | 156416003 | 73 | 30 | 71% | 283 | 31 | 70% | 270 | 34 | 72% | 317 |
| neprilysin isoform X1 | 578807443 | 86 | 30 | 52% | 176 | 30 | 53% | 161 | 30 | 53% | 165 |
| importin subunit beta-1 isoform 1 | 19923142 | 97 | 30 | 57% | 149 | 26 | 52% | 127 | 27 | 49% | 143 |
| isoleucine--tRNA ligase, mitochondrial precursor | 46852147 | 114 | 30 | 42% | 127 | 24 | 40% | 114 | 29 | 43% | 141 |
| slit homolog 2 protein isoform X1 | 530376407 | 170 | 29 | 26% | 73 | 32 | 29% | 83 | 26 | 23% | 74 |
| alpha-enolase isoform 1 | 4503571 | 47 | 29 | 75% | 285 | 30 | 74% | 266 | 31 | 76% | 280 |
| citrate synthase, mitochondrial precursor | 38327625 | 52 | 29 | 60% | 278 | 30 | 60% | 254 | 28 | 62% | 271 |
| pyruvate kinase PKM isoform b | 33286420 | 58 | 29 | 57% | 170 | 28 | 55% | 156 | 27 | 58% | 176 |
| long-chain-fatty-acid--CoA ligase 1 isoform X3 | 530377352 | 78 | 28 | 42% | 149 | 31 | 46% | 178 | 32 | 48% | 174 |
| ectonucleotide pyrophosphatase/phosphodiesterase family member 3 | 111160296 | 100 | 28 | 43% | 168 | 30 | 51% | 197 | 31 | 52% | 180 |
| tubulin beta-4B chain | 5174735 | 50 | 28 | 79% | 478 | 29 | 79% | 471 | 28 | 79% | 511 |
| dipeptidyl peptidase 4 | 18765694 | 88 | 28 | 34% | 92 | 29 | 33% | 128 | 26 | 33% | 109 |
| 2-oxoglutarate dehydrogenase, mitochondrial isoform 1 precursor | 51873036 | 116 | 28 | 43% | 138 | 27 | 45% | 128 | 28 | 48% | 140 |
| leucine-rich repeat-containing protein 37B precursor | 53829385 | 106 | 27 | 40% | 347 | 29 | 40% | 294 | 30 | 40% | 332 |
| tubulin alpha-3C/D chain | 156564363 | 50 | 27 | 71% | 489 | 28 | 76% | 449 | 30 | 78% | 491 |
| sperm equatorial segment protein 1 precursor | 21717832 | 39 | 27 | 52% | 305 | 28 | 55% | 333 | 30 | 61% | 322 |
| saccharopine dehydrogenase-like oxidoreductase | 55770836 | 47 | 27 | 81% | 525 | 28 | 83% | 517 | 29 | 83% | 575 |
| T-complex protein 1 subunit epsilon | 24307939 | 60 | 27 | 64% | 171 | 27 | 69% | 154 | 28 | 65% | 179 |
| protein disulfide-isomerase A4 precursor | 4758304 | 73 | 27 | 48% | 122 | 26 | 39% | 100 | 31 | 47% | 117 |
| T-complex protein 1 subunit beta isoform 1 | 5453603 | 57 | 27 | 60% | 225 | 24 | 56% | 209 | 26 | 61% | 251 |
| succinyl-CoA:3-ketoacid coenzyme A transferase 1, mitochondrial precursor | 4557817 | 56 | 27 | 69% | 319 | 24 | 61% | 220 | 24 | 68% | 273 |
| carboxypeptidase D isoform 1 precursor | 22202611 | 153 | 26 | 27% | 86 | 31 | 31% | 93 | 32 | 34% | 106 |
| nuclear pore complex protein Nup93 isoform X1 | 530424559 | 93 | 26 | 52% | 130 | 30 | 43% | 119 | 29 | 47% | 119 |
| fumarate hydratase, mitochondrial | 19743875 | 55 | 26 | 63% | 346 | 29 | 65% | 369 | 27 | 65% | 423 |
| dolichyl-diphosphooligosaccharide--protein glycosyltransferase subunit 1 precursor | 4506675 | 69 | 26 | 54% | 189 | 28 | 53% | 213 | 28 | 58% | 214 |
| presequence protease, mitochondrial isoform 2 precursor | 41352061 | 117 | 26 | 38% | 82 | 24 | 40% | 105 | 25 | 41% | 109 |
| prolactin-inducible protein precursor | 4505821 | 17 | 26 | 77% | 802 | 19 | 77% | 1259 | 25 | 77% | 949 |
| very long-chain specific acyl-CoA dehydrogenase, mitochondrial isoform 3 | 394025723 | 73 | 25 | 52% | 161 | 30 | 58% | 156 | 27 | 50% | 159 |
| aspartate aminotransferase, mitochondrial isoform 1 precursor | 73486658 | 48 | 25 | 72% | 144 | 29 | 74% | 159 | 26 | 71% | 160 |
| sodium/potassium-transporting ATPase subunit alpha-4 isoform 1 | 153946397 | 114 | 25 | 41% | 107 | 27 | 44% | 136 | 24 | 39% | 127 |
| ruvB-like 2 | 5730023 | 51 | 25 | 66% | 185 | 26 | 63% | 152 | 28 | 71% | 184 |
| T-complex protein 1 subunit delta isoform a | 38455427 | 58 | 25 | 58% | 143 | 26 | 58% | 166 | 24 | 51% | 161 |
| sperm acrosome membrane-associated protein 1 precursor | 13569934 | 32 | 25 | 62% | 322 | 25 | 62% | 460 | 22 | 62% | 368 |
| protein disulfide-isomerase precursor | 20070125 | 57 | 25 | 58% | 143 | 24 | 54% | 161 | 25 | 54% | 186 |
| 2,4-dienoyl-CoA reductase, mitochondrial precursor | 4503301 | 36 | 25 | 77% | 386 | 24 | 77% | 514 | 24 | 78% | 606 |
| T-complex protein 1 subunit alpha isoform a | 57863257 | 60 | 25 | 63% | 182 | 24 | 51% | 139 | 24 | 63% | 182 |
| cytochrome b-c1 complex subunit 1, mitochondrial precursor | 46593007 | 53 | 25 | 71% | 211 | 24 | 66% | 188 | 23 | 66% | 224 |
| leucine-rich repeat-containing protein 37A3 isoform X2 | 530429826 | 184 | 25 | 17% | 178 | 23 | 17% | 186 | 22 | 17% | 190 |
| cullin-associated NEDD8-dissociated protein 1 | 21361794 | 136 | 25 | 34% | 87 | 22 | 31% | 78 | 23 | 32% | 85 |
| 26S proteasome non-ATPase regulatory subunit 1 isoform 1 | 25777600 | 106 | 24 | 32% | 94 | 29 | 37% | 104 | 25 | 35% | 80 |
| malate dehydrogenase, mitochondrial isoform 1 precursor | 21735621 | 36 | 24 | 65% | 394 | 26 | 66% | 439 | 25 | 66% | 421 |
| acrosin-binding protein precursor | 17999524 | 61 | 24 | 57% | 207 | 25 | 62% | 277 | 27 | 65% | 273 |
| clusterin preproprotein | 355594753 | 52 | 24 | 43% | 310 | 23 | 43% | 338 | 24 | 43% | 338 |
| phosphoglycerate kinase 2 | 31543397 | 45 | 24 | 71% | 154 | 21 | 70% | 150 | 23 | 74% | 152 |
| dynein heavy chain 17, axonemal | 256542310 | 509 | 23 | 6.30% | 51 | 32 | 9.40% | 81 | 30 | 8.50% | 74 |
| filamin-B isoform 2 | 105990514 | 278 | 23 | 14% | 54 | 28 | 18% | 64 | 32 | 20% | 74 |
| cullin-3 isoform X1 | 578805029 | 92 | 23 | 32% | 82 | 23 | 42% | 74 | 21 | 36% | 74 |
| zona pellucida-binding protein 1 isoform 1 precursor | 229577313 | 40 | 23 | 69% | 557 | 21 | 61% | 415 | 22 | 67% | 458 |
| 6-phosphofructokinase type C isoform X2 | 530392191 | 86 | 22 | 31% | 108 | 25 | 38% | 111 | 27 | 39% | 129 |
| agrin precursor | 54873613 | 215 | 22 | 18% | 49 | 22 | 19% | 66 | 22 | 19% | 67 |
| prostatic acid phosphatase isoform TM-PAP precursor | 197116348 | 48 | 22 | 46% | 215 | 19 | 42% | 235 | 20 | 48% | 237 |
| mannosyl-oligosaccharide glucosidase isoform 1 | 149999606 | 92 | 22 | 38% | 72 | 19 | 37% | 55 | 20 | 41% | 71 |
| glucose-6-phosphate isomerase isoform X2 | 530416229 | 63 | 22 | 65% | 152 | 18 | 50% | 117 | 20 | 51% | 148 |
| 4-trimethylaminobutyraldehyde dehydrogenase | 115387104 | 56 | 21 | 48% | 129 | 23 | 54% | 108 | 22 | 48% | 142 |
| heat shock 70 protein 1-like isoform X4 | 530428008 | 70 | 21 | 53% | 192 | 22 | 56% | 183 | 26 | 61% | 217 |
| dihydrolipoyl dehydrogenase, mitochondrial isoform 1 precursor | 91199540 | 54 | 21 | 59% | 303 | 21 | 58% | 266 | 21 | 64% | 327 |
| adipocyte plasma membrane-associated protein | 24308201 | 46 | 21 | 58% | 147 | 20 | 54% | 148 | 21 | 56% | 159 |
| calnexin precursor | 66933005 | 68 | 21 | 50% | 137 | 20 | 48% | 116 | 19 | 45% | 130 |
| carnitine O-acetyltransferase isoform 2 | 383209673 | 69 | 21 | 51% | 111 | 19 | 38% | 108 | 21 | 41% | 124 |
| myeloperoxidase precursor | 4557759 | 84 | 21 | 30% | 96 | 19 | 25% | 84 | 19 | 24% | 86 |
| fatty-acid amide hydrolase 1 | 166795287 | 63 | 21 | 63% | 99 | 16 | 54% | 88 | 18 | 54% | 102 |
| rab GDP dissociation inhibitor beta isoform 1 | 6598323 | 51 | 21 | 61% | 85 | 14 | 44% | 63 | 21 | 60% | 85 |
| cytochrome b-c1 complex subunit 2, mitochondrial precursor | 50592988 | 48 | 20 | 64% | 258 | 24 | 69% | 237 | 22 | 65% | 246 |
| sperm-associated antigen 6 isoform 1 | 6912678 | 55 | 20 | 66% | 132 | 22 | 75% | 111 | 21 | 73% | 120 |
| T-complex protein 1 subunit gamma isoform a | 63162572 | 61 | 20 | 55% | 127 | 20 | 49% | 111 | 22 | 52% | 137 |
| protein MENT precursor | 20149646 | 37 | 20 | 66% | 321 | 19 | 66% | 338 | 19 | 62% | 318 |
| lysine--tRNA ligase isoform 1 | 194272210 | 71 | 20 | 39% | 50 | 19 | 37% | 59 | 13 | 26% | 39 |
| ADP/ATP translocase 4 | 13775208 | 35 | 20 | 53% | 141 | 18 | 53% | 158 | 23 | 67% | 161 |
| L-lactate dehydrogenase A-like 6B | 15082234 | 42 | 20 | 57% | 138 | 18 | 59% | 119 | 20 | 65% | 164 |
| stress-70 protein, mitochondrial precursor | 24234688 | 74 | 20 | 43% | 134 | 17 | 35% | 118 | 20 | 39% | 131 |
| glucosidase 2 subunit beta isoform X1 | 578833384 | 61 | 20 | 44% | 107 | 17 | 31% | 92 | 18 | 31% | 93 |
| T-complex protein 1 subunit zeta isoform a | 4502643 | 58 | 20 | 37% | 115 | 16 | 35% | 103 | 19 | 36% | 117 |
| thioredoxin domain-containing protein 3 | 148839372 | 67 | 20 | 57% | 72 | 15 | 34% | 52 | 19 | 49% | 60 |
| importin subunit alpha-1 | 4504897 | 58 | 20 | 61% | 90 | 14 | 54% | 93 | 18 | 64% | 102 |
| mucin-5B precursor | 301172750 | 596 | 19 | 4.70% | 63 | 31 | 8.00% | 90 | 25 | 6.60% | 84 |
| valine--tRNA ligase isoform X1 | 530382523 | 141 | 19 | 16% | 63 | 24 | 20% | 94 | 25 | 22% | 87 |
| carboxypeptidase Z isoform 1 precursor | 62388877 | 74 | 19 | 46% | 147 | 23 | 47% | 136 | 22 | 50% | 146 |
| myosin-9 | 12667788 | 227 | 19 | 15% | 54 | 22 | 15% | 86 | 23 | 14% | 67 |
| calreticulin precursor | 4757900 | 48 | 19 | 70% | 183 | 22 | 71% | 172 | 21 | 78% | 182 |
| heat shock 70 protein 4L | 31541941 | 95 | 19 | 31% | 81 | 21 | 33% | 86 | 22 | 32% | 90 |
| proteasome activator complex subunit 4 | 163644283 | 211 | 19 | 15% | 45 | 21 | 16% | 63 | 20 | 15% | 60 |
| medium-chain specific acyl-CoA dehydrogenase, mitochondrial isoform b precursor | 187960098 | 47 | 19 | 56% | 153 | 20 | 55% | 152 | 20 | 54% | 174 |
| probable C-mannosyltransferase DPY19L2 isoform X1 | 578823598 | 85 | 19 | 28% | 87 | 20 | 26% | 103 | 19 | 27% | 102 |
| short-chain specific acyl-CoA dehydrogenase, mitochondrial precursor | 4557233 | 44 | 19 | 67% | 136 | 19 | 73% | 137 | 21 | 75% | 144 |
| calcium-binding mitochondrial carrier protein Aralar1 | 21361103 | 75 | 19 | 41% | 71 | 17 | 39% | 68 | 19 | 41% | 73 |
| peroxiredoxin-4 precursor | 5453549 | 31 | 19 | 67% | 97 | 17 | 62% | 95 | 16 | 65% | 102 |
| elongation factor 1-gamma | 4503481 | 50 | 19 | 57% | 111 | 16 | 57% | 119 | 19 | 56% | 142 |
| bifunctional glutamate/proline--tRNA ligase | 62241042 | 171 | 18 | 19% | 47 | 26 | 25% | 64 | 21 | 19% | 57 |
| dynactin subunit 1 isoform 5 | 299890871 | 137 | 18 | 20% | 54 | 22 | 27% | 61 | 24 | 27% | 64 |
| fructose-bisphosphate aldolase A isoform 1 | 34577112 | 39 | 18 | 77% | 115 | 20 | 82% | 132 | 18 | 79% | 129 |
| succinate dehydrogenase [ubiquinone] iron-sulfur subunit, mitochondrial precursor | 115387094 | 32 | 18 | 64% | 99 | 19 | 59% | 130 | 20 | 65% | 134 |
| heat shock protein HSP 90-beta isoform X1 | 530381931 | 83 | 18 | 57% | 86 | 18 | 58% | 69 | 17 | 54% | 90 |
| pyruvate dehydrogenase E1 component subunit beta, mitochondrial isoform 1 precursor | 156564403 | 39 | 18 | 65% | 143 | 17 | 64% | 177 | 16 | 64% | 180 |
| prenylcysteine oxidase 1 precursor | 166795301 | 57 | 18 | 50% | 95 | 16 | 47% | 89 | 18 | 47% | 98 |
| cAMP-dependent protein kinase type II-alpha regulatory subunit isoform X1 | 530372834 | 46 | 18 | 59% | 74 | 16 | 53% | 68 | 17 | 57% | 80 |
| 26S proteasome non-ATPase regulatory subunit 2 isoform 1 | 25777602 | 100 | 18 | 30% | 48 | 14 | 21% | 44 | 12 | 18% | 39 |
| clathrin heavy chain 1 isoform X2 | 530411491 | 192 | 17 | 12% | 55 | 22 | 15% | 73 | 21 | 12% | 62 |
| ruvB-like 1 | 4506753 | 50 | 17 | 60% | 108 | 19 | 63% | 117 | 19 | 61% | 124 |
| cathelicidin antimicrobial peptide preproprotein | 348041314 | 20 | 17 | 62% | 113 | 18 | 61% | 139 | 18 | 61% | 114 |
| glutathione S-transferase Mu 3 | 23065552 | 27 | 17 | 69% | 113 | 18 | 74% | 144 | 18 | 70% | 123 |
| dipeptidase 3 isoform a precursor | 193211608 | 56 | 17 | 37% | 122 | 17 | 39% | 107 | 18 | 39% | 123 |
| epididymal sperm-binding protein 1 precursor | 301601648 | 26 | 17 | 78% | 188 | 17 | 78% | 195 | 17 | 72% | 200 |
| acrosin precursor | 148613878 | 46 | 17 | 43% | 207 | 16 | 40% | 191 | 17 | 41% | 203 |
| aspartate aminotransferase, cytoplasmic | 4504067 | 46 | 17 | 64% | 70 | 16 | 68% | 76 | 17 | 57% | 71 |
| delta(3,5)-Delta(2,4)-dienoyl-CoA isomerase, mitochondrial precursor | 70995211 | 36 | 17 | 64% | 179 | 16 | 64% | 272 | 16 | 60% | 215 |
| 3-ketoacyl-CoA thiolase, mitochondrial | 167614485 | 42 | 17 | 59% | 86 | 16 | 58% | 79 | 16 | 57% | 91 |
| NADH dehydrogenase [ubiquinone] flavoprotein 1, mitochondrial isoform 1 precursor | 20149568 | 51 | 17 | 58% | 70 | 16 | 53% | 66 | 16 | 47% | 71 |
| prostate-specific antigen isoform 1 preproprotein | 4502173 | 29 | 17 | 84% | 339 | 16 | 84% | 371 | 15 | 84% | 338 |
| protein disulfide-isomerase A6 isoform d precursor | 5031973 | 48 | 17 | 48% | 99 | 16 | 50% | 135 | 15 | 48% | 110 |
| alpha-actinin-4 | 12025678 | 105 | 17 | 27% | 48 | 16 | 31% | 47 | 15 | 27% | 40 |
| ras-related protein Rab-2A isoform a | 4506365 | 24 | 17 | 70% | 176 | 15 | 67% | 187 | 17 | 68% | 181 |
| apoptosis-inducing factor 1, mitochondrial isoform 1 precursor | 4757732 | 67 | 17 | 41% | 80 | 12 | 33% | 49 | 16 | 38% | 66 |
| V-type proton ATPase catalytic subunit A | 19913424 | 68 | 16 | 44% | 50 | 17 | 43% | 51 | 16 | 42% | 56 |
| vesicular integral-membrane protein VIP36 precursor | 5803023 | 40 | 16 | 60% | 121 | 16 | 60% | 143 | 16 | 60% | 147 |
| transmembrane emp24 domain-containing protein 10 precursor | 98986464 | 25 | 16 | 43% | 104 | 16 | 56% | 145 | 16 | 48% | 122 |
| 3-hydroxyisobutyrate dehydrogenase, mitochondrial precursor | 23308751 | 35 | 16 | 61% | 136 | 16 | 63% | 161 | 15 | 61% | 140 |
| 3-hydroxyacyl-CoA dehydrogenase type-2 isoform 1 | 4758504 | 27 | 16 | 85% | 74 | 16 | 89% | 101 | 14 | 89% | 95 |
| mycophenolic acid acyl-glucuronide esterase, mitochondrial isoform 1 precursor | 8923001 | 34 | 16 | 58% | 122 | 15 | 58% | 130 | 19 | 61% | 134 |
| retinal dehydrogenase 1 | 21361176 | 55 | 16 | 53% | 40 | 15 | 47% | 47 | 18 | 57% | 52 |
| ferritin, mitochondrial precursor | 29126241 | 28 | 16 | 60% | 106 | 15 | 68% | 124 | 14 | 55% | 116 |
| elongation factor 2 | 4503483 | 95 | 16 | 31% | 61 | 15 | 29% | 56 | 13 | 26% | 59 |
| superoxide dismutase [Mn], mitochondrial isoform A precursor | 67782307 | 25 | 16 | 73% | 104 | 14 | 82% | 141 | 15 | 85% | 115 |
| EGF-like repeat and discoidin I-like domain-containing protein 3 isoform 1 precursor | 31317224 | 54 | 16 | 50% | 49 | 12 | 38% | 42 | 13 | 37% | 41 |
| cysteine-rich secretory protein 1 isoform 1 precursor | 25121982 | 28 | 16 | 61% | 123 | 12 | 60% | 124 | 12 | 69% | 137 |
| lon protease homolog, mitochondrial isoform 1 precursor | 21396489 | 106 | 16 | 24% | 42 | 12 | 21% | 45 | 10 | 16% | 30 |
| izumo sperm-egg fusion protein 4 isoform 1 precursor | 89903025 | 24 | 16 | 55% | 191 | 10 | 52% | 254 | 16 | 55% | 218 |
| electron transfer flavoprotein subunit alpha, mitochondrial isoform a | 4503607 | 35 | 15 | 67% | 114 | 17 | 71% | 116 | 16 | 63% | 121 |
| lactadherin isoform a preproprotein | 167830475 | 43 | 15 | 59% | 105 | 17 | 59% | 91 | 16 | 59% | 87 |
| erlin-2 isoform X2 | 530387549 | 38 | 15 | 53% | 64 | 17 | 60% | 57 | 14 | 58% | 57 |
| NADPH--cytochrome P450 reductase | 127139033 | 77 | 15 | 36% | 53 | 17 | 41% | 58 | 14 | 35% | 56 |
| enoyl-CoA hydratase, mitochondrial | 194097323 | 31 | 15 | 64% | 77 | 16 | 64% | 112 | 17 | 72% | 88 |
| proteasome subunit beta type-1 | 4506193 | 26 | 15 | 65% | 86 | 15 | 70% | 92 | 14 | 66% | 90 |
| AFG3-like protein 2 | 300192933 | 89 | 15 | 23% | 35 | 15 | 26% | 38 | 13 | 22% | 44 |
| prosaposin isoform a preproprotein | 11386147 | 58 | 15 | 36% | 81 | 14 | 35% | 82 | 17 | 43% | 84 |
| isocitrate dehydrogenase [NADP] cytoplasmic | 538917681 | 47 | 15 | 43% | 52 | 14 | 43% | 62 | 13 | 38% | 58 |
| dehydrogenase/reductase SDR family member 7 precursor | 7706318 | 38 | 15 | 58% | 111 | 13 | 54% | 85 | 12 | 51% | 103 |
| heat shock 70 protein 1A/1B | 167466173 | 70 | 15 | 55% | 79 | 12 | 49% | 76 | 14 | 52% | 80 |
| plasma serine protease inhibitor preproprotein | 194018472 | 46 | 15 | 49% | 86 | 12 | 42% | 72 | 13 | 44% | 70 |
| protein ERGIC-53 precursor | 5031873 | 58 | 15 | 41% | 56 | 12 | 30% | 65 | 11 | 31% | 56 |
| SPARC-related modular calcium-binding protein 2 isoform 2 precursor | 262050673 | 50 | 15 | 46% | 75 | 11 | 32% | 62 | 14 | 46% | 66 |
| LETM1 and EF-hand domain-containing protein 1, mitochondrial precursor | 6912482 | 83 | 15 | 27% | 51 | 11 | 24% | 42 | 13 | 26% | 45 |
| acylamino-acid-releasing enzyme | 23510451 | 81 | 15 | 43% | 33 | 11 | 27% | 32 | 11 | 24% | 27 |
| electron transfer flavoprotein subunit beta isoform 1 | 4503609 | 28 | 14 | 51% | 76 | 16 | 68% | 87 | 15 | 57% | 94 |
| dolichyl-diphosphooligosaccharide--protein glycosyltransferase 48 subunit precursor | 20070197 | 51 | 14 | 52% | 102 | 15 | 51% | 93 | 16 | 52% | 94 |
| 26S proteasome non-ATPase regulatory subunit 3 | 25777612 | 61 | 14 | 28% | 57 | 14 | 36% | 72 | 13 | 34% | 65 |
| cAMP-dependent protein kinase type I-alpha regulatory subunit isoform a | 443497964 | 43 | 14 | 33% | 84 | 14 | 28% | 70 | 13 | 31% | 93 |
| dolichyl-diphosphooligosaccharide--protein glycosyltransferase subunit 2 isoform 1 precursor | 35493916 | 69 | 14 | 32% | 62 | 14 | 39% | 83 | 12 | 30% | 65 |
| isovaleryl-CoA dehydrogenase, mitochondrial isoform 1 precursor | 226958412 | 47 | 14 | 54% | 63 | 14 | 52% | 68 | 11 | 45% | 63 |
| glutamate carboxypeptidase 2 isoform 1 | 4758398 | 84 | 14 | 25% | 51 | 13 | 22% | 60 | 13 | 25% | 45 |
| triosephosphate isomerase isoform 1 | 4507645 | 27 | 14 | 74% | 61 | 13 | 71% | 59 | 12 | 71% | 47 |
| peroxiredoxin-5, mitochondrial isoform a precursor | 6912238 | 22 | 14 | 57% | 73 | 12 | 56% | 90 | 13 | 57% | 82 |
| succinyl-CoA ligase [ADP-forming] subunit beta, mitochondrial precursor | 11321583 | 50 | 14 | 27% | 62 | 12 | 23% | 54 | 12 | 25% | 65 |
| zinc-alpha-2-glycoprotein precursor | 4502337 | 34 | 14 | 51% | 62 | 11 | 35% | 65 | 13 | 50% | 76 |
| nucleoporin p54 isoform 1 | 26051237 | 55 | 13 | 40% | 41 | 15 | 38% | 46 | 15 | 43% | 52 |
| puromycin-sensitive aminopeptidase | 158937236 | 103 | 13 | 24% | 39 | 15 | 27% | 44 | 14 | 25% | 56 |
| mucin-6 precursor | 151301154 | 257 | 13 | 7.60% | 31 | 14 | 11% | 36 | 16 | 11% | 37 |
| glyceraldehyde-3-phosphate dehydrogenase isoform 1 | 576583524 | 36 | 13 | 66% | 151 | 14 | 68% | 155 | 14 | 72% | 163 |
| pyruvate dehydrogenase E1 component subunit alpha, testis-specific form, mitochondrial precursor | 4885543 | 43 | 13 | 44% | 73 | 13 | 41% | 74 | 16 | 60% | 76 |
| protein NipSnap homolog 3A | 22267436 | 28 | 13 | 62% | 60 | 13 | 68% | 84 | 14 | 69% | 70 |
| deoxyguanosine kinase, mitochondrial isoform a precursor | 18426967 | 32 | 13 | 54% | 72 | 13 | 58% | 72 | 13 | 54% | 82 |
| proteasome subunit alpha type-4 isoform 1 | 156713442 | 29 | 13 | 69% | 80 | 13 | 67% | 102 | 12 | 65% | 75 |
| serpin B6 isoform c | 425876766 | 44 | 13 | 47% | 44 | 13 | 47% | 45 | 12 | 41% | 52 |
| collagen alpha-1(XVIII) chain isoform 2 precursor | 110611233 | 136 | 13 | 15% | 50 | 12 | 14% | 65 | 17 | 18% | 68 |
| alpha-centractin | 5031569 | 43 | 13 | 52% | 83 | 12 | 45% | 65 | 16 | 62% | 71 |
| ATP synthase subunit d, mitochondrial isoform a | 5453559 | 18 | 13 | 74% | 94 | 12 | 72% | 95 | 14 | 74% | 109 |
| phosphoglycerate mutase 2 | 50593010 | 29 | 13 | 58% | 70 | 12 | 58% | 68 | 13 | 58% | 81 |
| protein DJ-1 isoform X1 | 530360487 | 20 | 13 | 76% | 51 | 12 | 76% | 67 | 13 | 76% | 58 |
| NADH dehydrogenase [ubiquinone] iron-sulfur protein 3, mitochondrial precursor | 4758788 | 30 | 13 | 52% | 51 | 12 | 49% | 61 | 12 | 49% | 65 |
| 14-3-3 protein epsilon | 5803225 | 29 | 13 | 46% | 52 | 12 | 46% | 56 | 11 | 46% | 49 |
| 2-hydroxyacyl-CoA lyase 1 isoform b | 548923731 | 61 | 13 | 35% | 36 | 11 | 31% | 26 | 12 | 34% | 32 |
| glyceraldehyde-3-phosphate dehydrogenase, testis-specific | 7657116 | 45 | 13 | 56% | 106 | 11 | 47% | 85 | 11 | 44% | 88 |
| calcium-binding mitochondrial carrier protein Aralar2 isoform 1 | 237649019 | 74 | 13 | 44% | 58 | 9 | 33% | 45 | 13 | 40% | 51 |
| mitochondria-eating protein | 21687119 | 61 | 13 | 28% | 49 | 8 | 23% | 50 | 14 | 29% | 57 |
| armadillo repeat-containing protein 3 isoform 1 | 189409124 | 96 | 13 | 21% | 38 | 8 | 16% | 19 | 12 | 19% | 34 |
| ATP synthase F(0) complex subunit B1, mitochondrial isoform X1 | 530362759 | 44 | 12 | 27% | 62 | 15 | 37% | 90 | 14 | 32% | 91 |
| enoyl-CoA delta isomerase 2, mitochondrial isoform 1 | 260275230 | 40 | 12 | 47% | 81 | 13 | 51% | 71 | 13 | 51% | 75 |
| cytochrome c1, heme protein, mitochondrial precursor | 21359867 | 35 | 12 | 58% | 103 | 13 | 58% | 155 | 12 | 58% | 126 |
| cytochrome c oxidase subunit 5B, mitochondrial precursor | 17017988 | 14 | 12 | 60% | 54 | 13 | 60% | 79 | 10 | 60% | 64 |
| ropporin-1A | 21359920 | 24 | 12 | 71% | 110 | 12 | 71% | 126 | 15 | 76% | 138 |
| proteasome subunit alpha type-6 isoform a | 23110944 | 27 | 12 | 46% | 40 | 12 | 62% | 65 | 14 | 70% | 59 |
| succinate-semialdehyde dehydrogenase, mitochondrial isoform 2 precursor | 4507229 | 57 | 12 | 53% | 63 | 12 | 43% | 55 | 14 | 47% | 51 |
| signal peptidase complex subunit 2 | 162417971 | 25 | 12 | 45% | 64 | 12 | 44% | 70 | 13 | 44% | 85 |
| enoyl-CoA delta isomerase 1, mitochondrial isoform 1 precursor | 62530384 | 33 | 12 | 55% | 66 | 12 | 55% | 81 | 12 | 55% | 81 |
| ATP synthase subunit O, mitochondrial precursor | 4502303 | 23 | 12 | 68% | 75 | 12 | 69% | 70 | 11 | 63% | 83 |
| prohibitin isoform 1 | 4505773 | 30 | 12 | 66% | 57 | 12 | 66% | 69 | 11 | 62% | 72 |
| electron transfer flavoprotein-ubiquinone oxidoreductase, mitochondrial isoform 1 precursor | 119703746 | 68 | 12 | 32% | 40 | 12 | 30% | 46 | 10 | 23% | 34 |
| mitochondrial inner membrane protein isoform 1 | 154354964 | 84 | 12 | 24% | 48 | 11 | 27% | 48 | 16 | 32% | 66 |
| proteasome subunit alpha type-1 isoform 1 | 23110935 | 30 | 12 | 62% | 51 | 10 | 51% | 56 | 14 | 69% | 57 |
| proteasome subunit alpha type-2 | 4506181 | 26 | 12 | 51% | 54 | 10 | 51% | 61 | 13 | 51% | 59 |
| EF-hand calcium-binding domain-containing protein 1 isoform a | 13375787 | 24 | 12 | 70% | 47 | 10 | 70% | 46 | 12 | 66% | 49 |
| ES1 protein homolog, mitochondrial-like isoform X1 | 578836294 | 28 | 12 | 66% | 61 | 10 | 51% | 93 | 11 | 63% | 74 |
| endoplasmic reticulum resident protein 44 precursor | 52487191 | 47 | 12 | 42% | 54 | 10 | 37% | 41 | 11 | 37% | 41 |
| glutathione S-transferase P | 4504183 | 23 | 12 | 69% | 45 | 10 | 70% | 63 | 11 | 69% | 57 |
| extracellular matrix protein 1 isoform 3 precursor | 322302700 | 64 | 12 | 30% | 41 | 10 | 31% | 36 | 10 | 28% | 41 |
| protein sel-1 homolog 1 isoform 1 precursor | 19923669 | 89 | 12 | 28% | 32 | 10 | 26% | 28 | 10 | 22% | 31 |
| membrane-associated progesterone receptor component 1 isoform 1 | 5729875 | 22 | 12 | 57% | 42 | 9 | 60% | 38 | 10 | 53% | 33 |
| sulfhydryl oxidase 1 isoform a precursor | 13325075 | 83 | 12 | 22% | 25 | 9 | 19% | 26 | 9 | 19% | 26 |
| maltase-glucoamylase, intestinal isoform X1 | 578814724 | 312 | 11 | 5.60% | 18 | 20 | 10% | 50 | 19 | 10% | 42 |
| sodium/potassium-transporting ATPase subunit alpha-1 isoform a | 21361181 | 113 | 11 | 32% | 33 | 15 | 37% | 56 | 15 | 36% | 51 |
| galectin-3-binding protein precursor | 5031863 | 65 | 11 | 19% | 41 | 14 | 18% | 54 | 13 | 21% | 57 |
| dynein intermediate chain 2, axonemal isoform X4 | 530412670 | 69 | 11 | 37% | 39 | 14 | 36% | 32 | 12 | 33% | 36 |
| prohibitin-2 isoform 1 | 221307584 | 33 | 11 | 36% | 45 | 13 | 48% | 61 | 12 | 45% | 49 |
| heat shock cognate 71 protein isoform X1 | 578822169 | 71 | 11 | 41% | 46 | 13 | 39% | 47 | 12 | 41% | 52 |
| ras-related protein Rab-14 | 19923483 | 24 | 11 | 67% | 42 | 13 | 70% | 56 | 10 | 67% | 44 |
| ubiquitin-like modifier-activating enzyme 1 isoform X1 | 530421539 | 123 | 11 | 17% | 35 | 12 | 17% | 33 | 15 | 22% | 43 |
| peroxiredoxin-6 | 4758638 | 25 | 11 | 62% | 44 | 12 | 68% | 49 | 14 | 70% | 59 |
| hyaluronidase PH-20 isoform 1 | 21314606 | 58 | 11 | 32% | 80 | 12 | 30% | 101 | 13 | 36% | 88 |
| arachidonate 15-lipoxygenase B isoform d | 85067501 | 76 | 11 | 32% | 32 | 12 | 36% | 39 | 12 | 36% | 41 |
| solute carrier family 2, facilitated glucose transporter member 14 isoform a | 23592238 | 56 | 11 | 21% | 67 | 12 | 21% | 81 | 11 | 21% | 86 |
| proteasome subunit beta type-5 isoform 1 | 4506201 | 28 | 11 | 46% | 38 | 11 | 46% | 45 | 13 | 50% | 35 |
| aldose reductase | 4502049 | 36 | 11 | 62% | 36 | 11 | 60% | 40 | 13 | 68% | 46 |
| isocitrate dehydrogenase [NAD] subunit alpha, mitochondrial precursor | 5031777 | 40 | 11 | 40% | 77 | 11 | 37% | 49 | 12 | 44% | 63 |
| hydroxyacyl-coenzyme A dehydrogenase, mitochondrial isoform 1 precursor | 296179427 | 36 | 11 | 55% | 127 | 11 | 57% | 144 | 11 | 55% | 131 |
| lipoprotein lipase precursor | 4557727 | 53 | 11 | 36% | 48 | 11 | 33% | 54 | 9 | 28% | 51 |
| proteasome subunit alpha type-5 isoform 1 | 23110942 | 26 | 11 | 61% | 47 | 10 | 52% | 52 | 11 | 60% | 50 |
| izumo sperm-egg fusion protein 2 isoform X2 | 578833934 | 24 | 11 | 50% | 44 | 10 | 50% | 57 | 11 | 50% | 56 |
| adenylate kinase isoenzyme 1 isoform X1 | 530390694 | 23 | 11 | 54% | 66 | 10 | 54% | 70 | 11 | 56% | 72 |
| uncharacterized protein C9orf9 | 33285006 | 19 | 11 | 80% | 63 | 10 | 80% | 67 | 10 | 80% | 87 |
| acyl-CoA dehydrogenase family member 9, mitochondrial | 21361497 | 69 | 11 | 28% | 38 | 10 | 25% | 35 | 10 | 26% | 35 |
| cytochrome c oxidase subunit 4 isoform 1, mitochondrial isoform X1 | 530423589 | 20 | 11 | 50% | 52 | 10 | 51% | 61 | 9 | 43% | 59 |
| choline transporter-like protein 5 isoform B | 194239633 | 82 | 11 | 15% | 26 | 9 | 13% | 38 | 14 | 18% | 38 |
| epoxide hydrolase 1 | 209862837 | 53 | 11 | 35% | 27 | 9 | 25% | 29 | 11 | 41% | 26 |
| phosphate carrier protein, mitochondrial isoform b precursor | 4505775 | 40 | 11 | 37% | 39 | 9 | 34% | 35 | 10 | 36% | 38 |
| glutathione reductase, mitochondrial isoform 1 precursor | 50301238 | 56 | 11 | 30% | 42 | 9 | 27% | 35 | 10 | 24% | 39 |
| hypoxanthine-guanine phosphoribosyltransferase | 4504483 | 25 | 11 | 63% | 42 | 9 | 54% | 28 | 10 | 54% | 38 |
| NADH-cytochrome b5 reductase 3 isoform 3 | 284448551 | 38 | 11 | 44% | 37 | 9 | 34% | 40 | 8 | 37% | 34 |
| 3-hydroxyisobutyryl-CoA hydrolase, mitochondrial isoform 1 precursor | 37594471 | 43 | 11 | 41% | 30 | 9 | 36% | 21 | 7 | 25% | 20 |
| uncharacterized protein KIAA2013 precursor | 25286703 | 69 | 11 | 36% | 35 | 7 | 18% | 20 | 11 | 39% | 27 |
| calmegin isoform X1 | 578808711 | 70 | 11 | 25% | 24 | 7 | 23% | 18 | 10 | 27% | 23 |
| vesicle-fusing ATPase | 156564401 | 83 | 11 | 17% | 33 | 7 | 11% | 16 | 8 | 8.70% | 18 |
| heat shock 70 protein 4 | 38327039 | 94 | 11 | 20% | 23 | 6 | 13% | 15 | 9 | 12% | 22 |
| dihydrolipoyllysine-residue acetyltransferase component of pyruvate dehydrogenase complex, mitochondrial precursor | 31711992 | 69 | 10 | 29% | 65 | 13 | 32% | 70 | 12 | 32% | 65 |
| heat shock protein 75 , mitochondrial isoform 1 precursor | 155722983 | 80 | 10 | 23% | 31 | 13 | 26% | 36 | 9 | 17% | 28 |
| sarcoplasmic/endoplasmic reticulum calcium ATPase 2 isoform b | 24638454 | 115 | 10 | 14% | 22 | 12 | 18% | 37 | 15 | 20% | 42 |
| nodal modulator 2 isoform 2 precursor | 27734709 | 134 | 10 | 11% | 26 | 12 | 16% | 29 | 14 | 17% | 33 |
| cytochrome c oxidase subunit 5A, mitochondrial precursor | 190885499 | 17 | 10 | 70% | 52 | 12 | 71% | 62 | 12 | 71% | 69 |
| A-kinase anchor protein 3 | 507834051 | 95 | 10 | 15% | 24 | 12 | 21% | 34 | 12 | 20% | 36 |
| single-stranded DNA-binding protein, mitochondrial precursor | 374671775 | 17 | 10 | 74% | 52 | 12 | 67% | 84 | 11 | 73% | 73 |
| mesencephalic astrocyte-derived neurotrophic factor precursor | 299523086 | 21 | 10 | 51% | 34 | 12 | 54% | 55 | 10 | 51% | 43 |
| isochorismatase domain-containing protein 2, mitochondrial isoform 1 | 209969695 | 22 | 10 | 84% | 103 | 11 | 85% | 118 | 12 | 87% | 113 |
| serotransferrin precursor | 4557871 | 77 | 10 | 19% | 27 | 11 | 21% | 24 | 12 | 27% | 31 |
| transmembrane emp24 domain-containing protein 9 precursor | 39725636 | 27 | 10 | 43% | 41 | 11 | 42% | 70 | 11 | 43% | 54 |
| ezrin | 21614499 | 69 | 10 | 19% | 43 | 11 | 25% | 40 | 11 | 25% | 49 |
| ras-related protein Rab-11B | 190358517 | 24 | 10 | 52% | 39 | 11 | 50% | 35 | 11 | 52% | 43 |
| tetratricopeptide repeat protein 25 | 13899233 | 77 | 10 | 23% | 31 | 11 | 21% | 29 | 11 | 25% | 30 |
| bifunctional ATP-dependent dihydroxyacetone kinase/FAD-AMP lyase (cyclizing) | 20149621 | 59 | 10 | 32% | 37 | 11 | 31% | 30 | 11 | 31% | 40 |
| NADH dehydrogenase [ubiquinone] iron-sulfur protein 2, mitochondrial isoform X1 | 530364807 | 53 | 10 | 34% | 43 | 10 | 29% | 32 | 13 | 37% | 56 |
| endoplasmic reticulum resident protein 29 isoform 1 precursor | 5803013 | 29 | 10 | 50% | 56 | 10 | 55% | 61 | 12 | 57% | 62 |
| 26S proteasome non-ATPase regulatory subunit 8 | 156631005 | 40 | 10 | 37% | 36 | 10 | 37% | 40 | 10 | 37% | 40 |
| thioredoxin reductase 2, mitochondrial isoform 1 precursor | 22035672 | 57 | 10 | 37% | 32 | 10 | 34% | 33 | 9 | 27% | 29 |
| nucleobindin-2 isoform X1 | 578820554 | 50 | 10 | 42% | 57 | 10 | 33% | 49 | 8 | 25% | 47 |
| protein-L-isoaspartate(D-aspartate) O-methyltransferase isoform 1 | 226530908 | 30 | 10 | 60% | 33 | 10 | 65% | 41 | 8 | 44% | 32 |
| proteasome subunit alpha type-7 | 4506189 | 28 | 10 | 55% | 45 | 9 | 47% | 47 | 11 | 61% | 54 |
| stomatin-like protein 2, mitochondrial isoform a | 7305503 | 39 | 10 | 50% | 44 | 9 | 48% | 48 | 10 | 53% | 51 |
| NADH dehydrogenase [ubiquinone] 1 beta subcomplex subunit 9 isoform 1 | 6274550 | 22 | 10 | 66% | 29 | 9 | 59% | 35 | 9 | 61% | 27 |
| calcium-binding tyrosine phosphorylation-regulated protein isoform a | 24797108 | 53 | 10 | 27% | 56 | 8 | 24% | 72 | 12 | 30% | 72 |
| izumo sperm-egg fusion protein 1 precursor | 194097475 | 39 | 10 | 44% | 30 | 8 | 44% | 16 | 12 | 51% | 26 |
| A disintegrin and metalloproteinase with thrombospondin motifs 1 preproprotein | 50845384 | 105 | 10 | 14% | 34 | 8 | 12% | 24 | 11 | 14% | 41 |
| 6-phosphogluconate dehydrogenase, decarboxylating | 40068518 | 53 | 10 | 30% | 40 | 8 | 22% | 37 | 10 | 28% | 36 |
| 26S proteasome non-ATPase regulatory subunit 6 isoform 2 | 7661914 | 46 | 10 | 24% | 29 | 8 | 26% | 26 | 9 | 28% | 34 |
| sorbitol dehydrogenase | 156627571 | 38 | 10 | 40% | 50 | 8 | 34% | 33 | 8 | 34% | 35 |
| nuclear pore membrane glycoprotein 210-like isoform 1 precursor | 117414168 | 211 | 9 | 6.60% | 20 | 14 | 9.40% | 25 | 8 | 5.20% | 19 |
| Golgi apparatus protein 1 isoform 2 precursor | 224586815 | 136 | 9 | 9.60% | 31 | 13 | 15% | 28 | 16 | 20% | 44 |
| disintegrin and metalloproteinase domain-containing protein 32 precursor | 148664238 | 88 | 9 | 11% | 28 | 12 | 15% | 42 | 12 | 15% | 36 |
| cytochrome b-c1 complex subunit Rieske, mitochondrial | 163644321 | 30 | 9 | 39% | 60 | 12 | 67% | 77 | 11 | 65% | 77 |
| glypican-1 precursor | 167001141 | 62 | 9 | 23% | 25 | 12 | 32% | 35 | 10 | 28% | 26 |
| alpha-mannosidase 2C1 isoform 2 | 374532775 | 117 | 9 | 11% | 23 | 11 | 14% | 32 | 14 | 20% | 32 |
| atlastin-3 isoform 1 | 45827806 | 61 | 9 | 34% | 43 | 11 | 34% | 35 | 10 | 26% | 33 |
| complement component 1 Q subcomponent-binding protein, mitochondrial precursor | 4502491 | 31 | 9 | 55% | 45 | 11 | 56% | 61 | 9 | 53% | 47 |
| peptidyl-prolyl cis-trans isomerase B precursor | 4758950 | 24 | 9 | 45% | 49 | 10 | 45% | 51 | 13 | 51% | 52 |
| apolipoprotein A-I isoform X1 | 530398067 | 31 | 9 | 34% | 32 | 10 | 37% | 44 | 12 | 43% | 42 |
| sodium/potassium-transporting ATPase subunit beta-3 | 4502281 | 32 | 9 | 49% | 53 | 10 | 49% | 52 | 11 | 49% | 51 |
| elongation factor 1-alpha 1 | 4503471 | 50 | 9 | 31% | 35 | 10 | 29% | 53 | 11 | 42% | 59 |
| ADP/ATP translocase 2 | 156071459 | 33 | 9 | 35% | 21 | 10 | 31% | 24 | 11 | 40% | 29 |
| glycine--tRNA ligase precursor | 116805340 | 83 | 9 | 24% | 32 | 10 | 26% | 29 | 11 | 26% | 36 |
| metalloproteinase inhibitor 3 precursor | 4507513 | 24 | 9 | 55% | 40 | 10 | 59% | 56 | 10 | 59% | 57 |
| gamma-glutamyltranspeptidase 1 precursor | 572152963 | 61 | 9 | 14% | 42 | 10 | 16% | 54 | 10 | 14% | 52 |
| dynein light chain 1, axonemal isoform 1 | 164607156 | 22 | 9 | 53% | 41 | 10 | 47% | 51 | 10 | 53% | 46 |
| adenylate kinase 2, mitochondrial isoform a | 4502013 | 26 | 9 | 56% | 29 | 10 | 55% | 28 | 10 | 54% | 32 |
| V-type proton ATPase subunit B, brain isoform | 19913428 | 57 | 9 | 32% | 33 | 10 | 30% | 30 | 8 | 26% | 33 |
| voltage-dependent calcium channel subunit alpha-2/delta-2 isoform X1 | 530373385 | 129 | 9 | 6.80% | 25 | 10 | 12% | 24 | 7 | 6.30% | 18 |
| beta-lactamase-like protein 2 | 7705793 | 33 | 9 | 42% | 23 | 9 | 44% | 35 | 12 | 49% | 32 |
| isoaspartyl peptidase/L-asparaginase | 145275200 | 32 | 9 | 51% | 33 | 9 | 51% | 42 | 12 | 62% | 40 |
| axonemal dynein light intermediate polypeptide 1 | 37595560 | 32 | 9 | 41% | 25 | 9 | 37% | 25 | 12 | 50% | 36 |
| proteasome activator complex subunit 1 isoform 1 | 5453990 | 29 | 9 | 47% | 24 | 9 | 49% | 30 | 11 | 56% | 34 |
| proteasome subunit beta type-2 isoform 1 | 4506195 | 23 | 9 | 70% | 38 | 9 | 70% | 45 | 10 | 74% | 55 |
| glypican-4 precursor | 21614525 | 62 | 9 | 25% | 36 | 9 | 28% | 39 | 10 | 30% | 41 |
| hydroxyacylglutathione hydrolase, mitochondrial isoform 1 precursor | 94538322 | 34 | 9 | 47% | 38 | 9 | 34% | 48 | 10 | 53% | 44 |
| actin-related protein T2 | 29893808 | 42 | 9 | 49% | 31 | 9 | 46% | 33 | 10 | 51% | 46 |
| fumarylacetoacetate hydrolase domain-containing protein 2B | 40786394 | 35 | 9 | 46% | 66 | 9 | 46% | 85 | 9 | 46% | 80 |
| transcription factor A, mitochondrial isoform 1 precursor | 4507401 | 29 | 9 | 42% | 39 | 9 | 42% | 42 | 9 | 41% | 45 |
| G-protein coupled receptor 64 isoform 2 precursor | 119943116 | 110 | 9 | 11% | 45 | 9 | 11% | 45 | 9 | 11% | 48 |
| NADH dehydrogenase [ubiquinone] flavoprotein 2, mitochondrial precursor | 222080062 | 27 | 9 | 42% | 34 | 9 | 47% | 39 | 8 | 38% | 37 |
| NADH dehydrogenase [ubiquinone] 1 alpha subcomplex subunit 9, mitochondrial precursor | 6681764 | 43 | 9 | 29% | 41 | 9 | 29% | 33 | 8 | 26% | 31 |
| choline dehydrogenase, mitochondrial | 217272839 | 65 | 9 | 24% | 29 | 8 | 23% | 31 | 12 | 31% | 37 |
| cAMP-dependent protein kinase catalytic subunit alpha isoform 2 | 46909584 | 40 | 9 | 32% | 35 | 8 | 29% | 14 | 11 | 36% | 35 |
| 3-oxoacyl-[acyl-carrier-protein] synthase, mitochondrial isoform X1 | 578806264 | 49 | 9 | 42% | 28 | 8 | 32% | 19 | 10 | 45% | 25 |
| ATP synthase subunit gamma, mitochondrial isoform L (liver) precursor | 50345988 | 33 | 9 | 42% | 97 | 8 | 39% | 101 | 9 | 42% | 118 |
| copper homeostasis protein cutC homolog | 148596990 | 29 | 9 | 61% | 43 | 8 | 55% | 41 | 9 | 61% | 39 |
| membrane-associated progesterone receptor component 2 | 291621647 | 26 | 9 | 43% | 30 | 8 | 39% | 37 | 9 | 40% | 39 |
| heme oxygenase 2 isoform a | 555943918 | 42 | 9 | 36% | 50 | 8 | 30% | 44 | 8 | 28% | 50 |
| nuclear pore glycoprotein p62 | 301069416 | 53 | 9 | 23% | 35 | 8 | 22% | 38 | 8 | 20% | 35 |
| malectin precursor | 7661948 | 32 | 9 | 35% | 36 | 8 | 35% | 44 | 8 | 33% | 37 |
| carnitine O-palmitoyltransferase 1, muscle isoform isoform a | 4758050 | 88 | 9 | 13% | 31 | 7 | 7.60% | 29 | 14 | 18% | 48 |
| carbonic anhydrase 4 isoform X1 | 530412793 | 38 | 9 | 32% | 31 | 7 | 23% | 33 | 10 | 32% | 42 |
| sperm surface protein Sp17 | 8394343 | 17 | 9 | 61% | 38 | 7 | 64% | 48 | 9 | 61% | 38 |
| thioredoxin-related transmembrane protein 4 precursor | 40254947 | 39 | 9 | 23% | 67 | 7 | 23% | 72 | 8 | 19% | 77 |
| basigin isoform X1 | 530425374 | 29 | 9 | 43% | 25 | 7 | 33% | 29 | 8 | 44% | 27 |
| anterior gradient protein 2 homolog isoform X1 | 530384410 | 20 | 9 | 56% | 42 | 6 | 42% | 50 | 9 | 61% | 46 |
| NADH-cytochrome b5 reductase 2 isoform X1 | 530395465 | 38 | 9 | 41% | 22 | 6 | 32% | 30 | 9 | 43% | 39 |
| phospholipase A2, membrane associated precursor | 239915985 | 16 | 9 | 44% | 38 | 5 | 28% | 25 | 9 | 47% | 46 |
| mitochondrial-processing peptidase subunit beta precursor | 94538354 | 54 | 9 | 26% | 17 | 5 | 17% | 16 | 7 | 24% | 20 |
| tenascin precursor | 153946395 | 241 | 8 | 5.10% | 20 | 13 | 9.70% | 28 | 11 | 8.00% | 26 |
| integrin alpha-M isoform 1 precursor | 224831239 | 127 | 8 | 9.50% | 19 | 12 | 13% | 30 | 10 | 13% | 27 |
| solute carrier family 2, facilitated glucose transporter member 5 isoform X1 | 530360630 | 55 | 8 | 12% | 31 | 12 | 18% | 44 | 10 | 18% | 38 |
| nuclear pore complex protein Nup155 isoform 1 | 24430149 | 155 | 8 | 7.40% | 21 | 11 | 9.00% | 32 | 16 | 13% | 40 |
| DNA damage-binding protein 1 | 148529014 | 127 | 8 | 13% | 14 | 11 | 17% | 27 | 15 | 22% | 36 |
| matrix-remodeling-associated protein 5 precursor | 139948432 | 312 | 8 | 4.20% | 19 | 11 | 6.30% | 30 | 11 | 6.50% | 24 |
| peroxiredoxin-2 | 32189392 | 22 | 8 | 64% | 29 | 11 | 67% | 44 | 10 | 59% | 42 |
| peroxiredoxin-1 | 4505591 | 22 | 8 | 57% | 29 | 11 | 71% | 37 | 10 | 67% | 39 |
| cytochrome c oxidase subunit II (mitochondrion) | 251831110 | 26 | 8 | 45% | 94 | 11 | 61% | 112 | 9 | 61% | 110 |
| transmembrane and coiled-coil domain-containing protein 2 | 56847610 | 20 | 8 | 53% | 52 | 11 | 54% | 68 | 9 | 53% | 56 |
| translin isoform 1 | 4759270 | 26 | 8 | 38% | 34 | 11 | 59% | 44 | 8 | 44% | 42 |
| prostate and testis expressed protein 1 precursor | 19923082 | 14 | 8 | 56% | 45 | 11 | 71% | 78 | 6 | 56% | 59 |
| mitochondrial 2-oxoglutarate/malate carrier protein isoform 1 | 21361114 | 34 | 8 | 25% | 23 | 10 | 31% | 37 | 12 | 45% | 42 |
| alpha-aminoadipic semialdehyde synthase, mitochondrial | 13027640 | 102 | 8 | 19% | 23 | 10 | 20% | 23 | 12 | 19% | 25 |
| CDGSH iron-sulfur domain-containing protein 1 | 8923930 | 12 | 8 | 71% | 49 | 10 | 80% | 59 | 10 | 80% | 58 |
| WAP four-disulfide core domain protein 8 isoform X1 | 530418513 | 28 | 8 | 36% | 28 | 10 | 40% | 53 | 10 | 39% | 44 |
| dynein intermediate chain 1, axonemal isoform 2 | 526479830 | 80 | 8 | 11% | 18 | 10 | 25% | 23 | 9 | 17% | 22 |
| 14-3-3 protein zeta/delta isoform X1 | 530389315 | 42 | 8 | 26% | 38 | 9 | 29% | 52 | 12 | 31% | 47 |
| cytochrome c | 11128019 | 12 | 8 | 57% | 49 | 9 | 61% | 56 | 10 | 59% | 55 |
| T-complex protein 1 subunit zeta-2 isoform 1 | 58331173 | 58 | 8 | 38% | 49 | 9 | 32% | 48 | 8 | 36% | 51 |
| apolipoprotein O isoform X1 | 578837961 | 22 | 8 | 65% | 31 | 9 | 68% | 43 | 8 | 65% | 37 |
| tryptophan--tRNA ligase, cytoplasmic isoform X4 | 578826102 | 53 | 8 | 33% | 24 | 9 | 34% | 24 | 8 | 31% | 26 |
| complement decay-accelerating factor isoform 2 precursor | 168693643 | 49 | 8 | 27% | 39 | 9 | 28% | 35 | 6 | 19% | 36 |
| FUN14 domain-containing protein 2 | 24371248 | 21 | 8 | 48% | 31 | 8 | 53% | 30 | 10 | 54% | 36 |
| vesicle-associated membrane protein-associated protein A isoform 2 | 94721252 | 28 | 8 | 41% | 55 | 8 | 48% | 78 | 8 | 41% | 69 |
| proteasome subunit beta type-4 | 22538467 | 29 | 8 | 48% | 43 | 8 | 48% | 66 | 8 | 48% | 49 |
| epididymal secretory protein E1 precursor | 5453678 | 17 | 8 | 52% | 42 | 8 | 66% | 40 | 8 | 63% | 35 |
| azurocidin preproprotein | 11342670 | 27 | 8 | 45% | 26 | 8 | 51% | 41 | 8 | 51% | 32 |
| chitinase domain-containing protein 1 isoform a precursor | 218083142 | 45 | 8 | 34% | 29 | 8 | 37% | 21 | 7 | 21% | 25 |
| calmodulin isoform X1 | 578826144 | 17 | 8 | 79% | 33 | 7 | 76% | 40 | 10 | 79% | 38 |
| peroxisomal membrane protein 11B isoform 1 | 4505719 | 28 | 8 | 35% | 22 | 7 | 39% | 24 | 10 | 48% | 33 |
| NADH dehydrogenase [ubiquinone] 1 beta subcomplex subunit 10 | 4758774 | 21 | 8 | 51% | 40 | 7 | 52% | 34 | 9 | 57% | 40 |
| phospholipid hydroperoxide glutathione peroxidase, mitochondrial isoform A precursor | 75709200 | 22 | 8 | 47% | 18 | 7 | 45% | 21 | 9 | 50% | 32 |
| alkyldihydroxyacetonephosphate synthase, peroxisomal precursor | 4501993 | 73 | 8 | 24% | 17 | 7 | 21% | 16 | 9 | 20% | 14 |
| mRNA export factor isoform X2 | 530418462 | 45 | 8 | 32% | 21 | 7 | 29% | 17 | 9 | 41% | 20 |
| sperm acrosome membrane-associated protein 3 | 27777653 | 23 | 8 | 33% | 80 | 7 | 33% | 93 | 8 | 33% | 81 |
| cytosolic non-specific dipeptidase isoform X2 | 530414265 | 53 | 8 | 27% | 20 | 7 | 21% | 19 | 7 | 24% | 17 |
| malate dehydrogenase, cytoplasmic isoform 1 | 312283701 | 39 | 8 | 41% | 30 | 7 | 39% | 29 | 6 | 28% | 32 |
| secreted frizzled-related protein 1 precursor | 56117838 | 35 | 8 | 31% | 13 | 7 | 26% | 17 | 6 | 32% | 13 |
| synaptic vesicle membrane protein VAT-1 homolog | 18379349 | 42 | 8 | 20% | 22 | 7 | 17% | 19 | 5 | 17% | 18 |
| epimerase family protein SDR39U1 isoform 1 | 116812630 | 31 | 8 | 37% | 24 | 6 | 27% | 28 | 8 | 34% | 26 |
| peptidyl-prolyl cis-trans isomerase FKBP2 precursor | 17149844 | 16 | 8 | 66% | 23 | 6 | 61% | 33 | 7 | 61% | 24 |
| eukaryotic translation initiation factor 3 subunit M | 23397429 | 43 | 8 | 32% | 22 | 6 | 28% | 16 | 7 | 34% | 20 |
| 3'(2'),5'-bisphosphate nucleotidase 1 isoform 1 | 116812595 | 33 | 8 | 31% | 19 | 6 | 29% | 16 | 7 | 19% | 16 |
| binder of sperm protein homolog 1 isoform X1 | 530415450 | 16 | 8 | 58% | 24 | 6 | 58% | 25 | 6 | 58% | 21 |
| schlafen-like protein 1 isoform X1 | 530362068 | 46 | 8 | 23% | 38 | 6 | 20% | 25 | 6 | 22% | 31 |
| metalloreductase STEAP4 isoform 1 | 328887949 | 52 | 8 | 22% | 22 | 5 | 19% | 18 | 8 | 17% | 28 |
| disintegrin and metalloproteinase domain-containing protein 30 preproprotein | 31881770 | 89 | 8 | 14% | 28 | 5 | 12% | 27 | 8 | 14% | 34 |
| isocitrate dehydrogenase [NAD] subunit beta, mitochondrial isoform a precursor | 28178821 | 42 | 8 | 31% | 28 | 5 | 18% | 23 | 6 | 19% | 24 |
| membrane metallo-endopeptidase-like 1 | 239049391 | 89 | 8 | 18% | 15 | 5 | 7.70% | 18 | 6 | 10% | 24 |
| retinal dehydrogenase 2 isoform 1 | 25777724 | 57 | 8 | 34% | 15 | 5 | 20% | 8 | 6 | 24% | 13 |
| dipeptidyl peptidase 2 isoform X1 | 530426726 | 62 | 8 | 23% | 20 | 5 | 17% | 13 | 5 | 17% | 15 |
| glyoxylate reductase/hydroxypyruvate reductase | 6912396 | 36 | 8 | 47% | 27 | 4 | 29% | 12 | 7 | 44% | 23 |
| tissue alpha-L-fucosidase precursor | 119360348 | 54 | 8 | 28% | 17 | 4 | 16% | 13 | 6 | 23% | 14 |
| spectrin alpha chain, non-erythrocytic 1 isoform X2 | 578817799 | 289 | 7 | 4.00% | 17 | 16 | 9.70% | 40 | 13 | 6.40% | 28 |
| vacuolar protein sorting-associated protein 13A isoform B | 15619008 | 351 | 7 | 4.00% | 16 | 14 | 8.30% | 31 | 13 | 6.90% | 29 |
| laminin subunit alpha-5 precursor | 21264602 | 400 | 7 | 1.50% | 21 | 12 | 3.00% | 27 | 10 | 2.50% | 25 |
| aspartyl aminopeptidase | 156416028 | 53 | 7 | 31% | 23 | 10 | 41% | 22 | 10 | 37% | 24 |
| protein NipSnap homolog 1 isoform 1 | 193211616 | 33 | 7 | 40% | 28 | 10 | 43% | 46 | 9 | 51% | 30 |
| diablo homolog, mitochondrial isoform 2 | 21070978 | 21 | 7 | 51% | 27 | 10 | 73% | 51 | 8 | 67% | 37 |
| 14-3-3 protein theta | 5803227 | 28 | 7 | 38% | 28 | 10 | 43% | 33 | 6 | 38% | 26 |
| ATP-citrate synthase isoform X2 | 530412284 | 125 | 7 | 9.50% | 18 | 9 | 13% | 28 | 11 | 12% | 31 |
| L-xylulose reductase isoform 2 | 304571975 | 26 | 7 | 51% | 25 | 9 | 55% | 36 | 10 | 64% | 41 |
| carbonic anhydrase 2 | 4557395 | 29 | 7 | 43% | 21 | 9 | 53% | 31 | 10 | 57% | 32 |
| delta-1-pyrroline-5-carboxylate dehydrogenase, mitochondrial isoform a precursor | 25777734 | 62 | 7 | 15% | 19 | 9 | 24% | 23 | 10 | 24% | 22 |
| nicastrin isoform 2 | 589269130 | 77 | 7 | 19% | 31 | 9 | 24% | 42 | 9 | 24% | 43 |
| redox-regulatory protein FAM213A isoform 2 precursor | 344925834 | 24 | 7 | 40% | 26 | 9 | 44% | 35 | 9 | 37% | 35 |
| dihydrolipoyllysine-residue succinyltransferase component of 2-oxoglutarate dehydrogenase complex, mitochondrial isoform 1 precursor | 19923748 | 49 | 7 | 16% | 23 | 9 | 21% | 30 | 9 | 21% | 34 |
| proteasome subunit beta type-7 proprotein | 4506203 | 30 | 7 | 51% | 38 | 9 | 55% | 50 | 8 | 55% | 43 |
| protein DPCD | 39930355 | 23 | 7 | 47% | 18 | 9 | 53% | 21 | 7 | 47% | 17 |
| nitrilase homolog 1 isoform 3 | 297632348 | 34 | 7 | 27% | 28 | 8 | 32% | 36 | 11 | 41% | 37 |
| inositol monophosphatase 1 isoform 2 | 221625487 | 37 | 7 | 26% | 36 | 8 | 26% | 48 | 8 | 23% | 35 |
| putative lipoyltransferase 2, mitochondrial precursor | 221554520 | 25 | 7 | 40% | 21 | 8 | 47% | 33 | 8 | 47% | 23 |
| outer dense fiber protein 2 isoform 4 | 310750402 | 76 | 7 | 16% | 23 | 8 | 14% | 25 | 7 | 11% | 20 |
| proteasome subunit beta type-3 | 22538465 | 23 | 7 | 42% | 34 | 8 | 44% | 60 | 6 | 40% | 40 |
| transmembrane emp24 domain-containing protein 7 precursor | 32996709 | 25 | 7 | 44% | 32 | 7 | 38% | 45 | 9 | 35% | 37 |
| ecto-ADP-ribosyltransferase 3 isoform b precursor | 21361167 | 43 | 7 | 32% | 30 | 7 | 25% | 44 | 8 | 30% | 47 |
| transmembrane emp24 domain-containing protein 4 precursor | 33457308 | 26 | 7 | 36% | 30 | 7 | 41% | 41 | 8 | 46% | 32 |
| serine protease 58 precursor | 48255915 | 27 | 7 | 47% | 25 | 7 | 47% | 32 | 7 | 47% | 34 |
| peroxisomal membrane protein 11C isoform 1 | 18087833 | 27 | 7 | 44% | 23 | 7 | 44% | 33 | 6 | 33% | 30 |
| annexin A5 | 4502107 | 36 | 7 | 24% | 17 | 6 | 30% | 18 | 10 | 40% | 29 |
| large proline-rich protein BAG6 isoform X10 | 578811941 | 120 | 7 | 6.60% | 23 | 6 | 5.20% | 23 | 8 | 8.50% | 25 |
| 26S proteasome non-ATPase regulatory subunit 7 | 25777615 | 37 | 7 | 38% | 32 | 6 | 32% | 22 | 7 | 37% | 26 |
| dolichyl-diphosphooligosaccharide--protein glycosyltransferase subunit STT3A isoform a | 22749415 | 81 | 7 | 9.10% | 17 | 6 | 8.70% | 18 | 7 | 11% | 19 |
| nucleotide exchange factor SIL1 precursor | 11968009 | 52 | 7 | 22% | 24 | 6 | 18% | 20 | 7 | 21% | 20 |
| ras-related protein Rab-7a | 34147513 | 23 | 7 | 42% | 20 | 6 | 37% | 18 | 7 | 46% | 23 |
| titin isoform IC | 388998877 | 3994 | 7 | 0.17% | 7 | 6 | 0.08% | 12 | 6 | 0.12% | 7 |
| deoxyuridine 5'-triphosphate nucleotidohydrolase, mitochondrial isoform 1 precursor | 70906441 | 27 | 7 | 34% | 22 | 6 | 33% | 23 | 6 | 33% | 23 |
| dihydropteridine reductase | 208973246 | 26 | 7 | 40% | 13 | 6 | 37% | 15 | 6 | 33% | 20 |
| vesicle-associated membrane protein 3 | 4759300 | 11 | 7 | 53% | 43 | 6 | 41% | 54 | 5 | 41% | 42 |
| acid ceramidase isoform X1 | 530387776 | 43 | 7 | 27% | 30 | 6 | 24% | 23 | 5 | 24% | 19 |
| NADH dehydrogenase [ubiquinone] 1 alpha subcomplex subunit 8 | 7657369 | 20 | 7 | 47% | 23 | 6 | 52% | 22 | 5 | 40% | 21 |
| cytoskeleton-associated protein 4 | 19920317 | 66 | 7 | 15% | 17 | 6 | 15% | 13 | 5 | 13% | 15 |
| cytochrome b-c1 complex subunit 7 isoform 1 | 5454152 | 14 | 7 | 52% | 25 | 5 | 43% | 25 | 9 | 59% | 33 |
| receptor expression-enhancing protein 6 | 19923919 | 21 | 7 | 39% | 48 | 5 | 33% | 46 | 8 | 39% | 51 |
| apolipoprotein A-IV precursor | 71773110 | 45 | 7 | 26% | 19 | 5 | 18% | 21 | 6 | 22% | 21 |
| ADP-ribosyl cyclase 1 | 38454326 | 34 | 7 | 29% | 21 | 5 | 20% | 20 | 4 | 16% | 16 |
| serine/threonine-protein phosphatase PP1-gamma catalytic subunit isoform 2 | 350276247 | 39 | 7 | 27% | 22 | 5 | 16% | 13 | 4 | 17% | 14 |
| neuropathy target esterase isoform a | 260656037 | 151 | 7 | 12% | 18 | 4 | 3.70% | 12 | 4 | 4.60% | 6 |
| kinectin isoform a | 118498356 | 156 | 6 | 3.50% | 10 | 10 | 8.80% | 20 | 9 | 5.50% | 19 |
| peroxisomal multifunctional enzyme type 2 isoform 2 | 4504505 | 80 | 6 | 13% | 23 | 9 | 19% | 29 | 13 | 22% | 33 |
| dynactin subunit 2 isoform 3 | 387527974 | 44 | 6 | 32% | 27 | 9 | 39% | 25 | 8 | 37% | 19 |
| lysozyme-like protein 4 precursor | 21389465 | 16 | 6 | 58% | 22 | 9 | 65% | 34 | 7 | 65% | 32 |
| importin-5 isoform X2 | 530423350 | 126 | 6 | 7.20% | 19 | 8 | 11% | 26 | 10 | 13% | 30 |
| heat shock protein beta-1 | 4504517 | 23 | 6 | 62% | 27 | 8 | 66% | 46 | 8 | 71% | 30 |
| CD177 antigen precursor | 110735433 | 46 | 6 | 29% | 24 | 8 | 37% | 32 | 8 | 37% | 36 |
| transmembrane protein 126A isoform 1 | 14150017 | 22 | 6 | 47% | 25 | 8 | 62% | 47 | 8 | 63% | 32 |
| mitochondrial-processing peptidase subunit alpha isoform 1 precursor | 24308013 | 58 | 6 | 24% | 20 | 8 | 31% | 25 | 8 | 23% | 24 |
| histone H2B type 1-A | 24586679 | 14 | 6 | 41% | 68 | 8 | 48% | 87 | 7 | 43% | 82 |
| izumo sperm-egg fusion protein 3 isoform X1 | 530389630 | 28 | 6 | 34% | 37 | 8 | 41% | 42 | 7 | 38% | 32 |
| endophilin-B1 isoform X1 | 578799206 | 45 | 6 | 23% | 22 | 8 | 20% | 35 | 7 | 25% | 36 |
| peptidyl-prolyl cis-trans isomerase FKBP11 isoform 1 precursor | 7706131 | 22 | 6 | 48% | 19 | 8 | 59% | 23 | 7 | 53% | 23 |
| proteasome subunit alpha type-3 isoform 2 | 23110939 | 28 | 6 | 31% | 20 | 8 | 37% | 31 | 6 | 27% | 32 |
| 26S proteasome non-ATPase regulatory subunit 13 isoform 1 | 157502193 | 43 | 6 | 25% | 15 | 8 | 27% | 20 | 5 | 18% | 15 |
| UTP--glucose-1-phosphate uridylyltransferase isoform a | 48255966 | 57 | 6 | 13% | 22 | 7 | 18% | 19 | 9 | 27% | 24 |
| serum amyloid P-component precursor | 4502133 | 25 | 6 | 26% | 21 | 7 | 29% | 32 | 8 | 34% | 24 |
| cysteine desulfurase, mitochondrial isoform a precursor | 32307132 | 50 | 6 | 18% | 23 | 7 | 20% | 18 | 8 | 27% | 21 |
| AP-1 complex subunit beta-1 isoform b | 260436860 | 104 | 6 | 11% | 23 | 7 | 11% | 18 | 8 | 13% | 23 |
| synaptophysin-like protein 1 isoform a | 5803185 | 29 | 6 | 30% | 83 | 7 | 31% | 101 | 7 | 31% | 102 |
| lysosome-associated membrane glycoprotein 1 precursor | 112380628 | 45 | 6 | 12% | 40 | 7 | 13% | 40 | 7 | 14% | 41 |
| L-lactate dehydrogenase B chain | 291575128 | 37 | 6 | 27% | 24 | 7 | 24% | 25 | 7 | 35% | 28 |
| thioredoxin-dependent peroxide reductase, mitochondrial isoform b | 32483377 | 26 | 6 | 39% | 15 | 7 | 42% | 17 | 7 | 53% | 16 |
| phosphatidylglycerophosphatase and protein-tyrosine phosphatase 1 isoform 1 | 148224884 | 23 | 6 | 37% | 16 | 7 | 43% | 23 | 7 | 44% | 17 |
| mannose-1-phosphate guanyltransferase beta isoform 1 | 11761619 | 43 | 6 | 25% | 20 | 7 | 30% | 16 | 6 | 28% | 12 |
| aquaporin-7 isoform X1 | 530389886 | 37 | 6 | 28% | 16 | 7 | 28% | 26 | 6 | 28% | 18 |
| dynein light chain 2, cytoplasmic | 18087855 | 10 | 6 | 53% | 26 | 7 | 83% | 36 | 5 | 28% | 29 |
| phosphatidylethanolamine-binding protein 1 preproprotein | 4505621 | 21 | 6 | 57% | 25 | 7 | 61% | 27 | 5 | 48% | 23 |
| solute carrier organic anion transporter family member 6A1 isoform X2 | 530379669 | 79 | 6 | 10% | 17 | 7 | 12% | 17 | 5 | 8.50% | 15 |
| NAD(P)H-hydrate epimerase precursor | 91984773 | 32 | 6 | 44% | 18 | 7 | 50% | 26 | 4 | 23% | 16 |
| abhydrolase domain-containing protein 16A isoform a | 15100151 | 63 | 6 | 13% | 19 | 6 | 16% | 21 | 9 | 21% | 23 |
| 26S proteasome non-ATPase regulatory subunit 11 | 394953908 | 47 | 6 | 21% | 27 | 6 | 16% | 17 | 8 | 26% | 24 |
| perilipin-3 isoform 1 | 255958282 | 47 | 6 | 21% | 20 | 6 | 28% | 22 | 8 | 28% | 21 |
| parkin coregulated gene protein isoform 1 | 122939204 | 33 | 6 | 30% | 16 | 6 | 30% | 14 | 7 | 34% | 24 |
| histone H2AX | 4504253 | 15 | 6 | 32% | 42 | 6 | 32% | 46 | 6 | 32% | 45 |
| tricarboxylate transport protein, mitochondrial isoform a precursor | 21389315 | 34 | 6 | 29% | 34 | 6 | 28% | 39 | 6 | 26% | 29 |
| midkine isoform a precursor | 396080281 | 16 | 6 | 38% | 22 | 6 | 38% | 25 | 6 | 38% | 28 |
| NADH dehydrogenase [ubiquinone] iron-sulfur protein 8, mitochondrial isoform X3 | 530396822 | 20 | 6 | 42% | 24 | 6 | 42% | 34 | 6 | 42% | 30 |
| ras-related protein Rab-5C isoform b | 354721184 | 27 | 6 | 38% | 16 | 6 | 38% | 20 | 6 | 38% | 17 |
| polyribonucleotide nucleotidyltransferase 1, mitochondrial precursor | 188528628 | 86 | 6 | 9.80% | 15 | 6 | 13% | 12 | 6 | 12% | 16 |
| polyubiquitin-B precursor | 11024714 | 26 | 6 | 17% | 36 | 6 | 26% | 45 | 5 | 17% | 43 |
| mitochondrial thiamine pyrophosphate carrier | 186928856 | 36 | 6 | 16% | 13 | 6 | 19% | 14 | 5 | 16% | 14 |
| 60S ribosomal protein L12 | 4506597 | 18 | 6 | 55% | 17 | 6 | 55% | 23 | 5 | 45% | 19 |
| importin subunit alpha-3 | 4504901 | 58 | 6 | 27% | 17 | 6 | 22% | 13 | 5 | 22% | 15 |
| dihydroorotate dehydrogenase (quinone), mitochondrial | 45006951 | 43 | 6 | 23% | 13 | 6 | 25% | 16 | 3 | 12% | 12 |
| ubiquitin carboxyl-terminal hydrolase isozyme L1 | 21361091 | 25 | 6 | 48% | 11 | 6 | 42% | 13 | 3 | 30% | 5 |
| membrane cofactor protein isoform 4 precursor | 24432108 | 43 | 6 | 19% | 18 | 5 | 14% | 17 | 8 | 19% | 15 |
| endoplasmic reticulum lectin 1 isoform 2 precursor | 188528696 | 52 | 6 | 17% | 18 | 5 | 16% | 16 | 7 | 18% | 14 |
| CD59 glycoprotein preproprotein | 10835165 | 14 | 6 | 37% | 55 | 5 | 36% | 41 | 6 | 37% | 60 |
| probable inactive ribonuclease-like protein 13 precursor | 59276062 | 18 | 6 | 35% | 33 | 5 | 35% | 40 | 6 | 35% | 41 |
| inorganic pyrophosphatase 2, mitochondrial isoform 1 precursor | 29171702 | 38 | 6 | 19% | 15 | 5 | 25% | 20 | 6 | 29% | 27 |
| probable inactive serine protease 37 isoform 1 precursor | 285394164 | 26 | 6 | 41% | 15 | 5 | 37% | 18 | 6 | 37% | 19 |
| ERO1-like protein beta precursor | 239582761 | 54 | 6 | 19% | 19 | 5 | 16% | 13 | 6 | 18% | 17 |
| dolichyl-diphosphooligosaccharide--protein glycosyltransferase subunit STT3B | 30578410 | 94 | 6 | 8.00% | 15 | 5 | 7.10% | 16 | 6 | 8.40% | 17 |
| acetolactate synthase-like protein isoform X1 | 530414456 | 68 | 6 | 17% | 13 | 5 | 14% | 15 | 6 | 11% | 12 |
| NADP-dependent malic enzyme | 4505143 | 64 | 6 | 22% | 14 | 5 | 18% | 11 | 6 | 27% | 12 |
| radial spoke head protein 6 homolog A | 13540559 | 81 | 6 | 17% | 16 | 5 | 14% | 14 | 6 | 16% | 15 |
| eukaryotic translation initiation factor 3 subunit A | 4503509 | 167 | 6 | 7.70% | 15 | 5 | 4.60% | 14 | 5 | 5.90% | 10 |
| arylsulfatase A isoform a precursor | 313569795 | 54 | 6 | 21% | 17 | 5 | 22% | 17 | 5 | 22% | 16 |
| glycerol-3-phosphate dehydrogenase, mitochondrial isoform X1 | 530370176 | 81 | 6 | 15% | 12 | 5 | 12% | 13 | 4 | 8.10% | 10 |
| phosphoenolpyruvate carboxykinase [GTP], mitochondrial isoform 1 precursor | 66346721 | 71 | 6 | 11% | 11 | 5 | 11% | 12 | 4 | 9.20% | 10 |
| 2-oxoisovalerate dehydrogenase subunit beta, mitochondrial precursor | 34101272 | 43 | 6 | 19% | 15 | 4 | 16% | 6 | 9 | 33% | 20 |
| superoxide dismutase [Cu-Zn] | 4507149 | 16 | 6 | 60% | 29 | 4 | 43% | 26 | 8 | 64% | 35 |
| methylmalonyl-CoA mutase, mitochondrial isoform X1 | 530382073 | 83 | 6 | 11% | 14 | 4 | 11% | 10 | 8 | 11% | 17 |
| serine/threonine-protein phosphatase PGAM5, mitochondrial isoform 1 | 281604136 | 32 | 6 | 27% | 14 | 4 | 20% | 8 | 6 | 26% | 8 |
| lysosomal alpha-glucosidase isoform X1 | 530411863 | 105 | 6 | 13% | 13 | 4 | 11% | 9 | 6 | 12% | 16 |
| glutathione S-transferase omega-2 isoform 1 | 38016131 | 28 | 6 | 27% | 17 | 4 | 20% | 15 | 5 | 24% | 13 |
| arrestin domain-containing protein 5 | 122937478 | 38 | 6 | 14% | 16 | 4 | 11% | 12 | 5 | 14% | 12 |
| serine/threonine-protein phosphatase 2A activator isoform b | 30065643 | 37 | 6 | 37% | 17 | 4 | 31% | 12 | 5 | 34% | 16 |
| small membrane A-kinase anchor protein | 110349742 | 11 | 6 | 88% | 19 | 4 | 61% | 16 | 4 | 61% | 21 |
| cystatin-S precursor | 4503109 | 16 | 6 | 59% | 17 | 4 | 38% | 15 | 4 | 40% | 18 |
| coiled-coil domain-containing protein 63 isoform X1 | 578823376 | 66 | 6 | 17% | 14 | 4 | 12% | 10 | 4 | 5.70% | 11 |
| phosphoglycerate kinase 1 | 4505763 | 45 | 6 | 35% | 24 | 4 | 22% | 13 | 3 | 23% | 15 |
| V-type proton ATPase subunit E 2 | 18087815 | 26 | 6 | 20% | 18 | 3 | 14% | 7 | 6 | 20% | 20 |
| L-amino-acid oxidase isoform 1 precursor | 23821023 | 63 | 6 | 14% | 23 | 3 | 11% | 13 | 5 | 8.80% | 21 |
| SPRY domain-containing protein 7 isoform 1 | 20531765 | 22 | 6 | 45% | 16 | 3 | 29% | 14 | 5 | 42% | 17 |
| transmembrane emp24 domain-containing protein 2 precursor | 5803149 | 23 | 6 | 34% | 34 | 3 | 26% | 35 | 4 | 34% | 35 |
| phosphoglycolate phosphatase | 108796653 | 34 | 6 | 23% | 10 | 3 | 21% | 8 | 3 | 21% | 11 |
| vesicle-trafficking protein SEC22b precursor | 380837121 | 25 | 6 | 39% | 13 | 2 | 13% | 6 | 7 | 48% | 18 |
| NME1-NME2 protein | 66392203 | 30 | 6 | 22% | 13 | 2 | 10% | 2 | 5 | 22% | 10 |
| cleft lip and palate transmembrane protein 1 isoform 2 | 4502897 | 76 | 5 | 14% | 14 | 9 | 24% | 25 | 8 | 17% | 23 |
| cofilin-1 | 5031635 | 19 | 5 | 42% | 17 | 8 | 54% | 21 | 6 | 44% | 21 |
| phosphoglycerate mutase 1 | 4505753 | 29 | 5 | 52% | 23 | 7 | 58% | 40 | 8 | 59% | 25 |
| eukaryotic translation initiation factor 3 subunit L isoform 2 | 339275831 | 61 | 5 | 14% | 10 | 7 | 15% | 16 | 8 | 13% | 17 |
| plastin-3 isoform 2 | 288915539 | 68 | 5 | 27% | 16 | 7 | 28% | 21 | 7 | 26% | 21 |
| ubiquitin domain-containing protein 2 | 109659841 | 26 | 5 | 42% | 12 | 7 | 56% | 22 | 7 | 48% | 16 |
| ADP-ribosylation factor 1 | 4502201 | 21 | 5 | 34% | 19 | 7 | 50% | 29 | 6 | 43% | 20 |
| metaxin-2 | 5729937 | 30 | 5 | 44% | 13 | 7 | 55% | 23 | 6 | 51% | 21 |
| neurotrypsin precursor | 300244530 | 97 | 5 | 7.00% | 15 | 7 | 13% | 17 | 6 | 9.70% | 21 |
| prostasin preproprotein | 4506153 | 36 | 5 | 30% | 24 | 7 | 32% | 27 | 5 | 30% | 25 |
| adenylyl cyclase-associated protein 1 | 5453595 | 52 | 5 | 12% | 19 | 7 | 20% | 30 | 4 | 12% | 18 |
| dnaJ homolog subfamily C member 5 | 45504382 | 22 | 5 | 46% | 16 | 6 | 55% | 22 | 7 | 55% | 26 |
| 26S proteasome non-ATPase regulatory subunit 4 | 5292161 | 41 | 5 | 17% | 19 | 6 | 16% | 19 | 7 | 24% | 21 |
| L-lactate dehydrogenase A chain isoform 1 | 5031857 | 37 | 5 | 34% | 20 | 6 | 33% | 21 | 6 | 40% | 20 |
| multifunctional protein ADE2 isoform 2 | 5453539 | 47 | 5 | 22% | 22 | 6 | 30% | 24 | 6 | 28% | 23 |
| cat eye syndrome critical region protein 5 isoform 2 precursor | 14861834 | 46 | 5 | 18% | 14 | 6 | 30% | 14 | 6 | 27% | 18 |
| SET domain-containing protein 9 isoform 1 | 285397547 | 34 | 5 | 25% | 16 | 6 | 31% | 15 | 6 | 31% | 22 |
| ras-related protein Rab-18 isoform 1 | 10880989 | 23 | 5 | 32% | 14 | 6 | 41% | 16 | 6 | 41% | 15 |
| carboxypeptidase E preproprotein | 4503009 | 53 | 5 | 16% | 14 | 6 | 22% | 20 | 5 | 16% | 20 |
| transmembrane emp24 domain-containing protein 5 isoform 1 precursor | 282165814 | 26 | 5 | 25% | 19 | 6 | 35% | 28 | 5 | 25% | 21 |
| mitochondrial import receptor subunit TOM40 homolog | 193083120 | 38 | 5 | 23% | 13 | 6 | 32% | 15 | 5 | 23% | 14 |
| signal recognition particle receptor subunit beta | 284795266 | 30 | 5 | 24% | 11 | 6 | 27% | 15 | 5 | 24% | 17 |
| acyl-protein thioesterase 1 isoform 1 | 5453722 | 25 | 5 | 45% | 14 | 6 | 44% | 19 | 5 | 38% | 14 |
| pyruvate dehydrogenase E1 component subunit alpha, somatic form, mitochondrial isoform 2 precursor | 291084742 | 48 | 5 | 28% | 16 | 6 | 30% | 20 | 4 | 23% | 14 |
| HD domain-containing protein 2 | 116875826 | 23 | 5 | 35% | 14 | 6 | 47% | 16 | 4 | 36% | 15 |
| dipeptidyl peptidase 3 isoform 1 | 86792661 | 83 | 5 | 13% | 9 | 6 | 16% | 10 | 3 | 11% | 7 |
| cysteine-rich secretory protein 2 isoform X10 | 530382514 | 27 | 5 | 40% | 24 | 5 | 44% | 23 | 7 | 46% | 27 |
| synaptojanin-2-binding protein | 157388993 | 16 | 5 | 35% | 21 | 5 | 35% | 22 | 7 | 51% | 28 |
| proteasome activator complex subunit 2 | 30410792 | 27 | 5 | 32% | 20 | 5 | 33% | 16 | 7 | 45% | 27 |
| metalloproteinase inhibitor 1 precursor | 4507509 | 23 | 5 | 41% | 15 | 5 | 42% | 21 | 6 | 46% | 20 |
| testis-expressed sequence 101 protein isoform 1 | 194018544 | 29 | 5 | 24% | 57 | 5 | 24% | 57 | 5 | 24% | 52 |
| cadherin-1 preproprotein | 4757960 | 97 | 5 | 6.90% | 11 | 5 | 6.90% | 19 | 5 | 4.50% | 20 |
| elongation factor Ts, mitochondrial isoform 2 precursor | 171846268 | 35 | 5 | 34% | 14 | 5 | 30% | 22 | 5 | 34% | 17 |
| stromal cell-derived factor 2-like protein 1 precursor | 56243533 | 24 | 5 | 52% | 29 | 5 | 52% | 25 | 5 | 52% | 31 |
| gamma-glutamyl hydrolase precursor | 4503987 | 36 | 5 | 20% | 20 | 5 | 23% | 19 | 5 | 24% | 29 |
| probable threonine protease PRSS50 precursor | 7019563 | 43 | 5 | 16% | 18 | 5 | 18% | 17 | 5 | 18% | 32 |
| proteasome subunit alpha type-7-like isoform 2 | 68303563 | 28 | 5 | 50% | 10 | 5 | 50% | 19 | 5 | 50% | 13 |
| peptidase inhibitor 15 isoform X2 | 578815666 | 29 | 5 | 28% | 14 | 5 | 26% | 14 | 5 | 28% | 13 |
| cathepsin D preproprotein | 4503143 | 45 | 5 | 18% | 12 | 5 | 21% | 17 | 5 | 14% | 13 |
| 14-3-3 protein gamma | 21464101 | 28 | 5 | 40% | 15 | 5 | 39% | 18 | 5 | 43% | 17 |
| acylpyruvase FAHD1, mitochondrial isoform 2 | 13654274 | 25 | 5 | 43% | 17 | 5 | 43% | 17 | 5 | 43% | 18 |
| programmed cell death protein 6 isoform 2 | 389565483 | 22 | 5 | 48% | 12 | 5 | 48% | 11 | 5 | 48% | 13 |
| 26S protease regulatory subunit 10B | 195539395 | 46 | 5 | 13% | 16 | 5 | 17% | 15 | 5 | 17% | 15 |
| erlin-1 isoform X1 | 530392883 | 39 | 5 | 34% | 17 | 5 | 33% | 19 | 4 | 24% | 15 |
| serine protease 55 isoform 1 precursor | 110578663 | 39 | 5 | 22% | 19 | 5 | 17% | 17 | 4 | 19% | 19 |
| NADH dehydrogenase [ubiquinone] 1 beta subcomplex subunit 8, mitochondrial isoform 3 | 548923784 | 18 | 5 | 36% | 12 | 5 | 36% | 17 | 4 | 36% | 10 |
| UPF0598 protein C8orf82 | 49169841 | 24 | 5 | 34% | 13 | 5 | 38% | 14 | 4 | 30% | 12 |
| 60S ribosomal protein L13 isoform 1 | 15431295 | 24 | 5 | 19% | 12 | 5 | 23% | 10 | 4 | 18% | 10 |
| 4F2 cell-surface antigen heavy chain isoform c | 65506891 | 68 | 5 | 12% | 12 | 5 | 13% | 13 | 4 | 11% | 15 |
| isocitrate dehydrogenase [NADP], mitochondrial isoform 1 precursor | 28178832 | 51 | 5 | 14% | 14 | 5 | 15% | 11 | 3 | 11% | 7 |
| phospholipase A1 member A isoform 2 precursor | 332688256 | 48 | 5 | 17% | 12 | 4 | 13% | 12 | 7 | 23% | 15 |
| putative GTP cyclohydrolase 1 type 2 NIF3L1 isoform 1 | 209862879 | 42 | 5 | 21% | 17 | 4 | 17% | 10 | 6 | 27% | 16 |
| doublecortin domain-containing protein 2C | 566006166 | 42 | 5 | 20% | 30 | 4 | 18% | 25 | 6 | 25% | 28 |
| elongation factor 1-delta isoform 1 | 304555581 | 71 | 5 | 8.20% | 18 | 4 | 9.10% | 19 | 6 | 15% | 21 |
| ADP/ATP translocase 1 | 55749577 | 33 | 5 | 29% | 14 | 4 | 25% | 10 | 6 | 40% | 17 |
| beta-galactosidase-1-like protein isoform X1 | 530370954 | 74 | 5 | 12% | 18 | 4 | 11% | 14 | 6 | 13% | 15 |
| prostate and testis expressed protein 3 precursor | 222136622 | 12 | 5 | 43% | 19 | 4 | 32% | 16 | 5 | 37% | 19 |
| histidine triad nucleotide-binding protein 2, mitochondrial isoform X1 | 578816758 | 18 | 5 | 52% | 22 | 4 | 41% | 19 | 5 | 52% | 21 |
| 10 heat shock protein, mitochondrial | 4504523 | 11 | 5 | 49% | 11 | 4 | 43% | 15 | 5 | 63% | 11 |
| ras-related protein Rab-27B isoform X1 | 530414276 | 25 | 5 | 28% | 13 | 4 | 28% | 12 | 5 | 23% | 14 |
| actin-related protein 3 isoform 1 | 5031573 | 47 | 5 | 27% | 15 | 4 | 23% | 13 | 5 | 27% | 12 |
| dipeptidase 1 precursor | 4758190 | 46 | 5 | 22% | 15 | 4 | 17% | 14 | 5 | 23% | 17 |
| creatine kinase B-type | 21536286 | 43 | 5 | 20% | 14 | 4 | 13% | 12 | 4 | 11% | 16 |
| lysozyme-like protein 6 precursor | 317008577 | 17 | 5 | 53% | 21 | 4 | 49% | 26 | 4 | 49% | 23 |
| syntaxin-12 | 28933465 | 32 | 5 | 33% | 18 | 4 | 26% | 21 | 4 | 22% | 21 |
| von Willebrand factor A domain-containing protein 1 isoform 1 precursor | 40068485 | 47 | 5 | 23% | 13 | 4 | 18% | 14 | 4 | 18% | 14 |
| ADP-ribosylation factor-like protein 8B | 8922601 | 22 | 5 | 40% | 11 | 4 | 27% | 10 | 4 | 27% | 15 |
| prostaglandin-H2 D-isomerase precursor | 32171249 | 21 | 5 | 45% | 16 | 4 | 36% | 15 | 3 | 28% | 13 |
| calpain small subunit 1 | 51599151 | 28 | 5 | 25% | 10 | 4 | 27% | 10 | 3 | 25% | 8 |
| endoplasmic reticulum-Golgi intermediate compartment protein 3 isoform a | 38327615 | 44 | 5 | 22% | 17 | 3 | 13% | 10 | 6 | 24% | 22 |
| S-phase kinase-associated protein 1 isoform b | 25777713 | 19 | 5 | 33% | 13 | 3 | 27% | 11 | 5 | 33% | 16 |
| calpain-1 catalytic subunit | 12408656 | 82 | 5 | 16% | 12 | 3 | 9.40% | 9 | 4 | 10% | 9 |
| cathepsin F precursor | 6042196 | 53 | 5 | 6.60% | 12 | 3 | 6.60% | 12 | 4 | 6.00% | 14 |
| alpha/beta hydrolase domain-containing protein 17B isoform X1 | 578817535 | 32 | 5 | 37% | 14 | 3 | 22% | 10 | 4 | 31% | 10 |
| protein phosphatase 1 regulatory subunit 7 isoform 1 | 4506013 | 42 | 5 | 25% | 14 | 3 | 13% | 15 | 4 | 16% | 8 |
| dnaJ homolog subfamily C member 3 precursor | 5453980 | 58 | 5 | 20% | 11 | 3 | 12% | 10 | 4 | 12% | 7 |
| rab GDP dissociation inhibitor alpha | 4503971 | 51 | 5 | 42% | 8 | 3 | 26% | 9 | 4 | 36% | 14 |
| nucleoporin NUP53 isoform X2 | 578803633 | 33 | 5 | 33% | 13 | 3 | 22% | 14 | 3 | 16% | 8 |
| cysteine--tRNA ligase, cytoplasmic isoform X1 | 578820846 | 87 | 5 | 6.90% | 14 | 3 | 5.70% | 8 | 3 | 7.30% | 7 |
| protein FAM3B isoform a precursor | 46255030 | 26 | 5 | 26% | 12 | 3 | 20% | 9 | 3 | 16% | 8 |
| putative transferase CAF17, mitochondrial precursor | 58197556 | 38 | 5 | 30% | 9 | 2 | 9.30% | 9 | 3 | 13% | 10 |
| long-chain-fatty-acid--CoA ligase ACSBG2 isoform a | 574584718 | 74 | 5 | 9.80% | 8 | 1 | 1.40% | 1 | 5 | 9.80% | 11 |
| lactoylglutathione lyase | 118402586 | 21 | 5 | 36% | 16 | 1 | 11% | 3 | 4 | 33% | 14 |
| spectrin beta chain, non-erythrocytic 1 isoform X1 | 530368012 | 275 | 4 | 2.60% | 10 | 14 | 8.60% | 29 | 10 | 5.80% | 23 |
| transmembrane protein 190 precursor | 21040263 | 19 | 4 | 27% | 29 | 7 | 38% | 26 | 8 | 42% | 33 |
| receptor expression-enhancing protein 5 | 115430112 | 21 | 4 | 15% | 33 | 7 | 26% | 40 | 7 | 32% | 41 |
| uncharacterized protein KIAA1683 isoform c | 224451036 | 123 | 4 | 5.40% | 10 | 7 | 8.50% | 20 | 7 | 8.50% | 16 |
| mitochondrial dicarboxylate carrier isoform 2 | 20149598 | 31 | 4 | 22% | 19 | 7 | 30% | 26 | 6 | 23% | 25 |
| alpha-1-antitrypsin precursor | 189163532 | 47 | 4 | 14% | 14 | 7 | 25% | 18 | 5 | 17% | 16 |
| nucleoporin p58/p45 isoform a | 30102928 | 61 | 4 | 7.00% | 17 | 6 | 16% | 27 | 6 | 16% | 25 |
| cytochrome c oxidase subunit 6B1 | 4502985 | 10 | 4 | 58% | 33 | 6 | 58% | 35 | 4 | 58% | 31 |
| annexin A4 | 4502105 | 36 | 4 | 18% | 9 | 6 | 28% | 14 | 4 | 13% | 8 |
| armadillo repeat-containing protein 12 isoform a | 31542280 | 41 | 4 | 17% | 15 | 6 | 22% | 14 | 4 | 18% | 21 |
| serine/threonine-protein phosphatase with EF-hands 1 isoform 1b | 23312374 | 73 | 4 | 8.50% | 11 | 6 | 13% | 13 | 4 | 7.80% | 10 |
| pyruvate dehydrogenase protein X component, mitochondrial isoform 2 | 203098816 | 51 | 4 | 14% | 9 | 6 | 21% | 10 | 3 | 5.80% | 8 |
| proteasome subunit beta type-6 isoform 1 proprotein | 23110925 | 25 | 4 | 29% | 20 | 5 | 34% | 28 | 8 | 47% | 30 |
| UPF0468 protein C16orf80 | 8392875 | 23 | 4 | 28% | 12 | 5 | 44% | 14 | 7 | 50% | 22 |
| myosin light chain 6B | 313851001 | 23 | 4 | 30% | 21 | 5 | 41% | 24 | 6 | 36% | 27 |
| exportin-7 | 154448892 | 124 | 4 | 7.30% | 14 | 5 | 8.40% | 13 | 6 | 8.70% | 14 |
| putative tyrosine-protein phosphatase TPTE isoform alpha | 109689707 | 64 | 4 | 8.20% | 8 | 5 | 9.80% | 11 | 6 | 10% | 12 |
| prenylated Rab acceptor protein 1 | 222144309 | 21 | 4 | 22% | 27 | 5 | 23% | 35 | 5 | 22% | 32 |
| signal peptidase complex catalytic subunit SEC11A isoform 4 | 429836837 | 19 | 4 | 18% | 24 | 5 | 34% | 30 | 5 | 34% | 32 |
| minor histocompatibility antigen H13 isoform 1 | 23308607 | 41 | 4 | 19% | 15 | 5 | 21% | 18 | 5 | 21% | 17 |
| neutrophil gelatinase-associated lipocalin precursor | 38455402 | 23 | 4 | 32% | 8 | 5 | 39% | 13 | 5 | 35% | 9 |
| ras-related protein Rab-6A isoform b | 38679888 | 24 | 4 | 29% | 15 | 5 | 39% | 21 | 5 | 39% | 18 |
| isopentenyl-diphosphate Delta-isomerase 1 | 40018633 | 32 | 4 | 23% | 10 | 5 | 30% | 9 | 5 | 30% | 12 |
| 40S ribosomal protein S18 | 11968182 | 18 | 4 | 26% | 13 | 5 | 32% | 15 | 5 | 32% | 13 |
| importin subunit alpha-4 | 34485722 | 58 | 4 | 26% | 7 | 5 | 31% | 13 | 5 | 34% | 14 |
| cystatin-C precursor | 4503107 | 16 | 4 | 33% | 20 | 5 | 45% | 23 | 4 | 33% | 23 |
| mitochondrial pyruvate carrier 2 isoform X1 | 578800799 | 14 | 4 | 33% | 16 | 5 | 54% | 26 | 4 | 33% | 22 |
| ATP synthase subunit g, mitochondrial | 51479156 | 11 | 4 | 47% | 16 | 5 | 47% | 15 | 4 | 47% | 19 |
| cytochrome c oxidase subunit I (mitochondrion) | 251831109 | 57 | 4 | 9.70% | 19 | 5 | 9.20% | 18 | 4 | 9.70% | 12 |
| protein NipSnap homolog 2 isoform 1 | 4503937 | 34 | 4 | 21% | 11 | 5 | 28% | 13 | 4 | 21% | 13 |
| ATP-binding cassette sub-family B member 6, mitochondrial | 9955963 | 94 | 4 | 9.10% | 9 | 5 | 11% | 10 | 4 | 8.60% | 10 |
| ras-related protein Rab-2B isoform 1 | 21361884 | 24 | 4 | 65% | 14 | 5 | 65% | 20 | 3 | 57% | 12 |
| purine nucleoside phosphorylase | 157168362 | 32 | 4 | 23% | 7 | 5 | 30% | 10 | 3 | 15% | 5 |
| NADH dehydrogenase [ubiquinone] iron-sulfur protein 7, mitochondrial | 187281616 | 24 | 4 | 27% | 15 | 5 | 38% | 19 | 3 | 23% | 18 |
| transmembrane protein 205 | 15529966 | 21 | 4 | 22% | 15 | 4 | 22% | 20 | 6 | 27% | 18 |
| voltage-gated hydrogen channel 1 isoform X3 | 578824327 | 32 | 4 | 27% | 17 | 4 | 24% | 21 | 6 | 34% | 20 |
| growth hormone-inducible transmembrane protein | 118200356 | 37 | 4 | 15% | 13 | 4 | 14% | 17 | 6 | 18% | 17 |
| histone H4 | 4504301 | 11 | 4 | 41% | 22 | 4 | 41% | 20 | 5 | 48% | 23 |
| succinyl-CoA ligase [ADP/GDP-forming] subunit alpha, mitochondrial precursor | 109452591 | 36 | 4 | 20% | 20 | 4 | 21% | 22 | 5 | 23% | 23 |
| protein FAM71B | 222418633 | 65 | 4 | 6.30% | 16 | 4 | 15% | 15 | 5 | 16% | 12 |
| histone H2A-Bbd type 2/3 | 63029935 | 13 | 4 | 54% | 20 | 4 | 54% | 26 | 5 | 67% | 23 |
| adenosylhomocysteinase isoform 1 | 9951915 | 48 | 4 | 7.40% | 13 | 4 | 13% | 13 | 5 | 13% | 19 |
| grpE protein homolog 1, mitochondrial precursor | 24308295 | 24 | 4 | 22% | 12 | 4 | 22% | 7 | 5 | 26% | 13 |
| voltage-dependent anion-selective channel protein 3 isoform 2 | 208879465 | 31 | 4 | 13% | 11 | 4 | 22% | 8 | 5 | 21% | 11 |
| serine protease HTRA1 precursor | 4506141 | 51 | 4 | 11% | 9 | 4 | 13% | 11 | 5 | 11% | 14 |
| elongation factor Tu, mitochondrial precursor | 34147630 | 50 | 4 | 15% | 9 | 4 | 17% | 14 | 5 | 18% | 14 |
| peptidyl-prolyl cis-trans isomerase F, mitochondrial precursor | 5031987 | 22 | 4 | 36% | 9 | 4 | 28% | 9 | 5 | 35% | 13 |
| nucleoside diphosphate kinase homolog 5 | 4505413 | 24 | 4 | 37% | 7 | 4 | 34% | 13 | 5 | 43% | 13 |
| ropporin-1B isoform X1 | 530373960 | 24 | 4 | 74% | 41 | 4 | 74% | 69 | 4 | 74% | 44 |
| glycodelin isoform X2 | 530426623 | 18 | 4 | 27% | 23 | 4 | 27% | 30 | 4 | 27% | 31 |
| sperm acrosome-associated protein 5 precursor | 120952755 | 18 | 4 | 31% | 21 | 4 | 31% | 23 | 4 | 31% | 28 |
| maleylacetoacetate isomerase isoform 1 | 22202624 | 24 | 4 | 28% | 14 | 4 | 24% | 14 | 4 | 26% | 21 |
| acyl-coenzyme A thioesterase 13 isoform 1 | 8923812 | 15 | 4 | 38% | 17 | 4 | 38% | 24 | 4 | 38% | 17 |
| signal peptidase complex subunit 3 | 11345462 | 20 | 4 | 19% | 18 | 4 | 19% | 22 | 4 | 19% | 25 |
| lysosome membrane protein 2 isoform 1 precursor | 5031631 | 54 | 4 | 14% | 8 | 4 | 14% | 11 | 4 | 14% | 15 |
| 26S protease regulatory subunit 6B isoform 1 | 5729991 | 47 | 4 | 20% | 17 | 4 | 25% | 19 | 4 | 17% | 11 |
| dolichol-phosphate mannosyltransferase subunit 1 | 4503363 | 30 | 4 | 23% | 14 | 4 | 23% | 9 | 4 | 23% | 12 |
| methylcrotonoyl-CoA carboxylase beta chain, mitochondrial | 11545863 | 61 | 4 | 12% | 10 | 4 | 13% | 11 | 4 | 18% | 9 |
| CD9 antigen | 4502693 | 25 | 4 | 21% | 29 | 4 | 21% | 29 | 3 | 21% | 27 |
| dynein light chain 1, cytoplasmic | 4505813 | 10 | 4 | 53% | 23 | 4 | 83% | 19 | 3 | 53% | 27 |
| ATP synthase subunit delta, mitochondrial precursor | 50345991 | 17 | 4 | 58% | 12 | 4 | 58% | 12 | 3 | 38% | 12 |
| transmembrane protease serine 2 isoform 2 | 205360943 | 54 | 4 | 16% | 6 | 4 | 16% | 9 | 3 | 8.30% | 9 |
| rho GDP-dissociation inhibitor 1 isoform a | 4757768 | 23 | 4 | 51% | 14 | 4 | 51% | 12 | 3 | 36% | 9 |
| disintegrin and metalloproteinase domain-containing protein 29 isoform X1 | 530377098 | 93 | 4 | 6.20% | 10 | 4 | 4.00% | 8 | 3 | 2.90% | 8 |
| transmembrane protein 89 precursor | 56847630 | 18 | 4 | 42% | 8 | 4 | 42% | 9 | 3 | 31% | 9 |
| 40S ribosomal protein S7 | 4506741 | 22 | 4 | 39% | 10 | 4 | 39% | 11 | 3 | 23% | 9 |
| platelet-activating factor acetylhydrolase isoform X1 | 530382617 | 50 | 4 | 18% | 11 | 4 | 18% | 12 | 2 | 9.80% | 9 |
| eukaryotic translation initiation factor 3 subunit F | 4503519 | 38 | 4 | 17% | 10 | 3 | 14% | 10 | 7 | 41% | 14 |
| methionine--tRNA ligase, cytoplasmic | 14043022 | 101 | 4 | 11% | 8 | 3 | 11% | 12 | 7 | 13% | 17 |
| coiled-coil domain-containing protein 90B, mitochondrial isoform 1 precursor | 20149663 | 30 | 4 | 28% | 13 | 3 | 24% | 10 | 6 | 33% | 20 |
| oxidoreductase HTATIP2 isoform a precursor | 148728166 | 30 | 4 | 21% | 8 | 3 | 17% | 9 | 6 | 32% | 14 |
| UMP-CMP kinase 2, mitochondrial isoform 1 precursor | 117606370 | 49 | 4 | 16% | 12 | 3 | 14% | 9 | 5 | 23% | 12 |
| matrilin-2 isoform b precursor | 62548862 | 105 | 4 | 7.20% | 10 | 3 | 5.40% | 5 | 5 | 9.30% | 9 |
| acrosomal protein SP-10 isoform a precursor | 4501879 | 28 | 4 | 18% | 40 | 3 | 18% | 56 | 4 | 18% | 43 |
| serine protease inhibitor Kazal-type 2 isoform 1 precursor | 413081531 | 14 | 4 | 51% | 22 | 3 | 51% | 31 | 4 | 51% | 24 |
| mitochondrial pyruvate carrier 1-like protein | 306922396 | 15 | 4 | 44% | 11 | 3 | 34% | 8 | 4 | 44% | 20 |
| transmembrane 9 superfamily member 3 precursor | 190194386 | 68 | 4 | 8.70% | 10 | 3 | 7.30% | 7 | 4 | 5.10% | 11 |
| NADH dehydrogenase [ubiquinone] 1 alpha subcomplex subunit 5 isoform 4 | 538921301 | 13 | 4 | 45% | 12 | 3 | 45% | 16 | 4 | 45% | 17 |
| protein canopy homolog 2 isoform 1 precursor | 7657176 | 21 | 4 | 34% | 9 | 3 | 23% | 7 | 4 | 31% | 14 |
| phosphatidylserine synthase 2 | 13540555 | 56 | 4 | 12% | 11 | 3 | 8.60% | 8 | 4 | 12% | 11 |
| exportin-2 isoform 1 | 29029559 | 110 | 4 | 8.80% | 15 | 3 | 6.20% | 8 | 4 | 9.20% | 11 |
| chloride anion exchanger | 4557535 | 85 | 4 | 6.00% | 7 | 3 | 4.60% | 8 | 4 | 6.00% | 10 |
| zymogen granule protein 16 homolog B precursor | 94536866 | 23 | 4 | 23% | 10 | 3 | 19% | 7 | 3 | 19% | 9 |
| 60S ribosomal protein L6 isoform X2 | 578824095 | 33 | 4 | 18% | 10 | 3 | 18% | 10 | 3 | 14% | 12 |
| estradiol 17-beta-dehydrogenase 12 | 7705855 | 34 | 4 | 23% | 11 | 3 | 18% | 7 | 3 | 18% | 8 |
| cation-dependent mannose-6-phosphate receptor isoform X1 | 530399159 | 31 | 4 | 25% | 15 | 3 | 21% | 12 | 3 | 21% | 19 |
| UDP-glucose 6-dehydrogenase isoform 1 | 4507813 | 55 | 4 | 14% | 11 | 3 | 8.30% | 3 | 3 | 11% | 8 |
| reticulocalbin-2 isoform b precursor | 426214088 | 39 | 4 | 22% | 12 | 3 | 16% | 7 | 3 | 15% | 7 |
| ubiquitin carboxyl-terminal hydrolase 7 isoform 2 | 557129038 | 126 | 4 | 6.80% | 5 | 3 | 4.80% | 6 | 3 | 4.80% | 6 |
| 60S ribosomal protein L18 isoform 2 | 395132436 | 18 | 4 | 30% | 10 | 3 | 23% | 9 | 3 | 23% | 9 |
| CDGSH iron-sulfur domain-containing protein 2 | 56605994 | 15 | 4 | 36% | 9 | 3 | 30% | 6 | 3 | 24% | 8 |
| NADH dehydrogenase [ubiquinone] 1 beta subcomplex subunit 11, mitochondrial isoform 1 | 20127561 | 18 | 4 | 36% | 8 | 3 | 29% | 9 | 3 | 29% | 9 |
| 26S protease regulatory subunit 4 | 24430151 | 49 | 4 | 15% | 8 | 3 | 12% | 7 | 3 | 12% | 7 |
| sphingomyelin phosphodiesterase isoform 2 precursor | 300795589 | 70 | 4 | 7.50% | 8 | 3 | 5.70% | 9 | 2 | 4.10% | 6 |
| polymeric immunoglobulin receptor isoform X1 | 530366266 | 85 | 4 | 9.60% | 10 | 3 | 7.00% | 11 | 2 | 5.00% | 7 |
| ATPase family AAA domain-containing protein 3A isoform 1 | 283436220 | 71 | 4 | 9.10% | 7 | 3 | 9.80% | 3 | 1 | 3.30% | 3 |
| protein FAM71A | 282721094 | 63 | 4 | 7.10% | 10 | 2 | 3.70% | 6 | 5 | 9.80% | 12 |
| alpha-1B-glycoprotein precursor | 21071030 | 54 | 4 | 14% | 10 | 2 | 6.90% | 2 | 4 | 14% | 9 |
| 60S ribosomal protein L9 isoform X1 | 530376503 | 22 | 4 | 43% | 9 | 2 | 24% | 4 | 4 | 43% | 14 |
| dnaJ homolog subfamily A member 4 isoform 3 | 194328762 | 42 | 4 | 15% | 10 | 2 | 12% | 6 | 3 | 12% | 7 |
| protein EFR3 homolog A | 154146218 | 93 | 4 | 6.30% | 4 | 2 | 4.60% | 6 | 3 | 6.30% | 11 |
| lipase member I isoform X2 | 578836445 | 56 | 4 | 8.70% | 5 | 2 | 2.50% | 4 | 2 | 4.10% | 6 |
| FAD synthase isoform 4 | 296434312 | 32 | 4 | 26% | 7 | 2 | 19% | 4 | 2 | 12% | 5 |
| 26S protease regulatory subunit 7 isoform 1 | 4506209 | 49 | 4 | 16% | 9 | 2 | 6.90% | 6 | 1 | 5.10% | 2 |
| prostate and testis expressed protein 4 precursor | 221554530 | 11 | 4 | 36% | 9 | 1 | 11% | 5 | 4 | 37% | 15 |
| metaxin-1 isoform 1 | 597955330 | 51 | 4 | 12% | 9 | 1 | 3.40% | 3 | 2 | 5.60% | 4 |
| translin-associated factor X-interacting protein 1 isoform b | 110227629 | 77 | 4 | 11% | 8 | 1 | 2.90% | 3 | 2 | 5.80% | 6 |
| 72 type IV collagenase isoform a preproprotein | 11342666 | 74 | 4 | 14% | 8 | 1 | 2.90% | 3 | 2 | 7.30% | 4 |
| succinyl-CoA:3-ketoacid coenzyme A transferase 2, mitochondrial precursor | 11545841 | 56 | 4 | 20% | 12 | ni | ni | ni | 5 | 20% | 17 |
| fatty acid synthase | 41872631 | 273 | 3 | 1.80% | 5 | 7 | 4.50% | 15 | 7 | 3.20% | 14 |
| BPI fold-containing family B member 1 precursor | 40807482 | 52 | 3 | 13% | 10 | 6 | 25% | 15 | 6 | 24% | 17 |
| 14-3-3 protein beta/alpha | 4507949 | 28 | 3 | 23% | 8 | 6 | 36% | 16 | 5 | 30% | 17 |
| transmembrane 9 superfamily member 4 precursor | 164519076 | 75 | 3 | 4.70% | 5 | 6 | 14% | 17 | 4 | 9.20% | 12 |
| synaptosomal-associated protein 23 isoform SNAP23A | 18765729 | 23 | 3 | 19% | 9 | 6 | 40% | 15 | 3 | 22% | 9 |
| ras-related protein Ral-A precursor | 33946329 | 24 | 3 | 18% | 5 | 6 | 31% | 22 | 2 | 14% | 8 |
| GLIPR1-like protein 2 isoform 1 | 394025727 | 40 | 3 | 14% | 14 | 5 | 24% | 17 | 6 | 24% | 13 |
| cytoplasmic dynein 1 heavy chain 1 | 33350932 | 532 | 3 | 0.62% | 5 | 5 | 1.40% | 10 | 5 | 1.00% | 11 |
| hsc70-interacting protein isoform 1 | 19923193 | 41 | 3 | 12% | 18 | 5 | 17% | 22 | 5 | 17% | 24 |
| GTP-binding nuclear protein Ran | 5453555 | 24 | 3 | 17% | 11 | 5 | 33% | 15 | 5 | 22% | 16 |
| solute carrier family 2, facilitated glucose transporter member 3 | 5902090 | 54 | 3 | 15% | 26 | 5 | 16% | 26 | 4 | 15% | 34 |
| cytochrome b5 type B | 83921614 | 17 | 3 | 31% | 10 | 5 | 52% | 16 | 4 | 39% | 15 |
| CMT1A duplicated region transcript 15 protein | 56090618 | 21 | 3 | 32% | 10 | 5 | 38% | 23 | 4 | 32% | 16 |
| serine/threonine-protein phosphatase 2B catalytic subunit gamma isoform isoform 2 | 21361290 | 58 | 3 | 9.20% | 8 | 5 | 17% | 11 | 4 | 15% | 9 |
| oligoribonuclease, mitochondrial precursor | 224496106 | 27 | 3 | 17% | 13 | 5 | 19% | 19 | 3 | 17% | 12 |
| secretory carrier-associated membrane protein 2 isoform X1 | 578826542 | 42 | 3 | 12% | 7 | 5 | 21% | 17 | 3 | 12% | 11 |
| BAG family molecular chaperone regulator 5 isoform a | 62548856 | 56 | 3 | 11% | 9 | 5 | 18% | 11 | 3 | 12% | 8 |
| transmembrane 9 superfamily member 2 precursor | 4758874 | 76 | 3 | 4.40% | 11 | 4 | 6.80% | 10 | 7 | 15% | 18 |
| sperm-associated antigen 17 | 46240864 | 252 | 3 | 2.50% | 5 | 4 | 3.00% | 8 | 7 | 3.00% | 11 |
| fragile X mental retardation 1 neighbor protein | 22749199 | 29 | 3 | 22% | 18 | 4 | 25% | 25 | 6 | 38% | 25 |
| lysosome-associated membrane glycoprotein 2 isoform C precursor | 169790833 | 45 | 3 | 7.10% | 18 | 4 | 9.20% | 26 | 5 | 15% | 23 |
| cell division control protein 42 homolog isoform 1 precursor | 4757952 | 21 | 3 | 30% | 11 | 4 | 35% | 14 | 5 | 40% | 13 |
| malonyl-CoA decarboxylase, mitochondrial | 110349750 | 55 | 3 | 12% | 7 | 4 | 13% | 12 | 5 | 15% | 13 |
| speriolin isoform 1 | 197276668 | 62 | 3 | 12% | 6 | 4 | 14% | 12 | 5 | 16% | 13 |
| translocon-associated protein subunit delta isoform 2 precursor | 5454090 | 19 | 3 | 24% | 14 | 4 | 24% | 26 | 4 | 31% | 25 |
| putative protein FAM71E2 | 223972704 | 100 | 3 | 6.90% | 9 | 4 | 7.90% | 14 | 4 | 7.90% | 11 |
| dnaJ homolog subfamily B member 11 precursor | 7706495 | 41 | 3 | 12% | 6 | 4 | 12% | 14 | 4 | 12% | 14 |
| ATP synthase subunit s, mitochondrial isoform a precursor | 51558774 | 25 | 3 | 13% | 9 | 4 | 26% | 11 | 4 | 18% | 9 |
| integral membrane protein 2B | 11527402 | 30 | 3 | 20% | 7 | 4 | 21% | 12 | 4 | 21% | 9 |
| actin-like protein 7A | 5729720 | 49 | 3 | 5.50% | 7 | 4 | 16% | 8 | 4 | 20% | 12 |
| maestro heat-like repeat-containing protein family member 7 | 223278410 | 146 | 3 | 3.10% | 7 | 4 | 3.80% | 8 | 4 | 3.20% | 10 |
| 40S ribosomal protein S5 | 13904870 | 23 | 3 | 22% | 5 | 4 | 32% | 11 | 4 | 32% | 8 |
| protein FAM3D isoform X1 | 530371875 | 25 | 3 | 16% | 9 | 4 | 27% | 11 | 4 | 21% | 10 |
| NADH dehydrogenase [ubiquinone] iron-sulfur protein 5 | 297206823 | 13 | 3 | 30% | 8 | 4 | 47% | 15 | 3 | 30% | 9 |
| mimitin, mitochondrial | 29789409 | 20 | 3 | 33% | 8 | 4 | 38% | 12 | 3 | 33% | 10 |
| tetraspanin-16 isoform 2 | 542133096 | 26 | 3 | 16% | 9 | 4 | 16% | 15 | 3 | 16% | 12 |
| BRI3-binding protein precursor | 19923665 | 28 | 3 | 17% | 7 | 4 | 18% | 15 | 3 | 17% | 11 |
| glycerophosphodiester phosphodiesterase domain-containing protein 1 isoform 1 | 260763876 | 36 | 3 | 12% | 11 | 4 | 17% | 11 | 3 | 13% | 10 |
| normal mucosa of esophagus-specific gene 1 protein | 14165280 | 10 | 3 | 43% | 7 | 4 | 43% | 14 | 3 | 43% | 10 |
| transforming protein RhoA precursor | 10835049 | 22 | 3 | 25% | 7 | 4 | 32% | 10 | 3 | 23% | 9 |
| phosphatidylinositide phosphatase SAC1 | 190014578 | 67 | 3 | 6.00% | 9 | 4 | 7.70% | 12 | 3 | 6.00% | 9 |
| secretory carrier-associated membrane protein 3 isoform 1 | 16445419 | 38 | 3 | 15% | 7 | 4 | 10% | 8 | 3 | 15% | 9 |
| choline transporter-like protein 2 isoform X1 | 530415036 | 81 | 3 | 6.90% | 7 | 4 | 8.00% | 11 | 3 | 6.90% | 7 |
| 26S proteasome non-ATPase regulatory subunit 14 | 5031981 | 35 | 3 | 24% | 14 | 4 | 31% | 9 | 2 | 18% | 8 |
| mitochondrial import receptor subunit TOM22 homolog | 9910382 | 16 | 3 | 50% | 10 | 4 | 58% | 15 | 2 | 35% | 6 |
| 15 selenoprotein isoform 1 precursor | 42741648 | 18 | 3 | 23% | 6 | 4 | 41% | 10 | 2 | 13% | 6 |
| nuclease EXOG, mitochondrial isoform 1 precursor | 224451073 | 41 | 3 | 18% | 9 | 4 | 23% | 9 | 2 | 7.60% | 5 |
| long-chain-fatty-acid--CoA ligase 3 | 42794754 | 80 | 3 | 7.90% | 7 | 3 | 9.00% | 6 | 8 | 11% | 14 |
| 60S ribosomal protein L7 | 15431301 | 29 | 3 | 18% | 9 | 3 | 17% | 8 | 5 | 19% | 14 |
| ubiquitin carboxyl-terminal hydrolase isozyme L3 isoform 2 | 5174741 | 26 | 3 | 17% | 7 | 3 | 22% | 11 | 5 | 29% | 12 |
| profilin-3 | 71274140 | 15 | 3 | 39% | 6 | 3 | 39% | 6 | 5 | 55% | 11 |
| B-cell receptor-associated protein 31 isoform b | 213511012 | 28 | 3 | 9.80% | 13 | 3 | 9.80% | 18 | 4 | 16% | 16 |
| GLIPR1-like protein 1 isoform X1 | 530400035 | 36 | 3 | 7.30% | 13 | 3 | 7.30% | 20 | 4 | 14% | 17 |
| 3-mercaptopyruvate sulfurtransferase isoform 1 | 194473668 | 35 | 3 | 18% | 13 | 3 | 18% | 15 | 4 | 18% | 17 |
| growth/differentiation factor 15 precursor | 153792495 | 34 | 3 | 18% | 6 | 3 | 18% | 9 | 4 | 22% | 12 |
| uncharacterized protein C7orf61 | 51972226 | 24 | 3 | 22% | 10 | 3 | 22% | 10 | 4 | 25% | 12 |
| sorting and assembly machinery component 50 homolog | 225543166 | 52 | 3 | 13% | 9 | 3 | 12% | 6 | 4 | 11% | 7 |
| vitamin K epoxide reductase complex subunit 1-like protein 1 isoform 1 | 46309463 | 20 | 3 | 17% | 8 | 3 | 17% | 9 | 4 | 22% | 10 |
| catalase | 4557014 | 60 | 3 | 12% | 7 | 3 | 9.70% | 8 | 4 | 19% | 9 |
| sortilin isoform 1 preproprotein | 17149834 | 92 | 3 | 6.60% | 12 | 3 | 4.10% | 7 | 4 | 4.90% | 9 |
| mitochondrial fission 1 protein | 151108473 | 17 | 3 | 24% | 7 | 3 | 24% | 12 | 4 | 34% | 11 |
| S-formylglutathione hydrolase | 33413400 | 31 | 3 | 29% | 6 | 3 | 27% | 7 | 4 | 35% | 11 |
| prostaglandin G/H synthase 2 precursor | 4506265 | 69 | 3 | 7.30% | 5 | 3 | 6.30% | 9 | 4 | 10% | 8 |
| glutaredoxin-related protein 5, mitochondrial precursor | 42516576 | 17 | 3 | 31% | 7 | 3 | 28% | 9 | 4 | 39% | 10 |
| gastricsin isoform 1 preproprotein | 4505757 | 42 | 3 | 7.00% | 26 | 3 | 7.00% | 26 | 3 | 7.00% | 29 |
| translocon-associated protein subunit alpha precursor | 169404009 | 32 | 3 | 12% | 21 | 3 | 12% | 23 | 3 | 12% | 25 |
| equatorin isoform 1 precursor | 239582757 | 33 | 3 | 19% | 21 | 3 | 19% | 26 | 3 | 19% | 23 |
| CD81 antigen | 4757944 | 26 | 3 | 25% | 19 | 3 | 25% | 25 | 3 | 25% | 29 |
| probable ergosterol biosynthetic protein 28 | 6005719 | 16 | 3 | 26% | 12 | 3 | 26% | 19 | 3 | 26% | 15 |
| cytochrome c oxidase subunit 6C proprotein | 4758040 | 9 | 3 | 44% | 8 | 3 | 44% | 6 | 3 | 44% | 12 |
| voltage-dependent anion-selective channel protein 1 isoform X3 | 530380137 | 31 | 3 | 12% | 6 | 3 | 12% | 7 | 3 | 12% | 5 |
| epididymal secretory protein E3-beta precursor | 11641279 | 18 | 3 | 24% | 12 | 3 | 20% | 8 | 3 | 24% | 13 |
| erythrocyte band 7 integral membrane protein isoform a | 38016911 | 32 | 3 | 17% | 10 | 3 | 13% | 11 | 3 | 17% | 11 |
| 40S ribosomal protein S2 | 15055539 | 31 | 3 | 12% | 9 | 3 | 12% | 9 | 3 | 12% | 9 |
| leucine-rich repeat-containing protein 37A2 precursor | 116325993 | 188 | 3 | 16% | 8 | 3 | 18% | 14 | 3 | 18% | 15 |
| protein CutA isoform X1 | 578811722 | 19 | 3 | 33% | 13 | 3 | 33% | 9 | 3 | 33% | 12 |
| vesicle transport protein GOT1B | 7705636 | 15 | 3 | 20% | 7 | 3 | 20% | 8 | 3 | 20% | 12 |
| transmembrane emp24 domain-containing protein 1 precursor | 5803040 | 25 | 3 | 18% | 11 | 3 | 18% | 16 | 3 | 18% | 9 |
| synaptogyrin-1 isoform 1b | 22035698 | 21 | 3 | 23% | 9 | 3 | 23% | 16 | 3 | 23% | 11 |
| carboxypeptidase O precursor | 27436871 | 43 | 3 | 11% | 6 | 3 | 8.80% | 6 | 3 | 7.00% | 6 |
| peptidyl-prolyl cis-trans isomerase A | 10863927 | 18 | 3 | 33% | 7 | 3 | 35% | 7 | 3 | 33% | 11 |
| valacyclovir hydrolase precursor | 221316588 | 33 | 3 | 16% | 6 | 3 | 15% | 9 | 3 | 16% | 8 |
| dynactin subunit 3 isoform 3 | 526479822 | 18 | 3 | 33% | 8 | 3 | 33% | 8 | 3 | 34% | 8 |
| UPF0160 protein MYG1, mitochondrial precursor | 145275185 | 42 | 3 | 8.00% | 11 | 3 | 9.60% | 7 | 3 | 8.00% | 7 |
| mitochondrial pyruvate carrier 1 isoform 1 | 7706369 | 12 | 3 | 44% | 9 | 3 | 44% | 9 | 3 | 44% | 12 |
| 40S ribosomal protein S8 | 4506743 | 24 | 3 | 19% | 5 | 3 | 19% | 7 | 3 | 19% | 8 |
| ATP-binding cassette sub-family G member 2 isoform X2 | 530378437 | 72 | 3 | 6.70% | 7 | 3 | 6.70% | 7 | 3 | 6.70% | 9 |
| leucine-rich repeat-containing protein 52 precursor | 194018474 | 35 | 3 | 14% | 7 | 3 | 12% | 8 | 3 | 12% | 6 |
| actin, alpha cardiac muscle 1 proprotein | 4885049 | 42 | 3 | 44% | 34 | 3 | 34% | 31 | 2 | 36% | 34 |
| protein S100-A9 | 4506773 | 13 | 3 | 32% | 9 | 3 | 36% | 12 | 2 | 25% | 6 |
| BPI fold-containing family A member 3 isoform 2 precursor | 109627656 | 24 | 3 | 28% | 20 | 3 | 28% | 21 | 2 | 15% | 21 |
| delta-aminolevulinic acid dehydratase | 189083849 | 36 | 3 | 15% | 7 | 3 | 20% | 6 | 2 | 17% | 5 |
| lipid phosphate phosphohydrolase 1 isoform 2 | 29171738 | 32 | 3 | 13% | 8 | 3 | 13% | 13 | 2 | 10% | 9 |
| actin-related protein 2/3 complex subunit 4 isoform a | 5031595 | 20 | 3 | 18% | 9 | 3 | 18% | 9 | 2 | 11% | 6 |
| cell adhesion molecule 1 isoform 1 precursor | 148664190 | 49 | 3 | 17% | 7 | 3 | 17% | 14 | 2 | 11% | 9 |
| NADH dehydrogenase [ubiquinone] iron-sulfur protein 6, mitochondrial precursor | 4758792 | 14 | 3 | 37% | 12 | 3 | 37% | 7 | 2 | 27% | 8 |
| insulin-like peptide INSL6 precursor | 38569396 | 25 | 3 | 19% | 8 | 3 | 19% | 6 | 2 | 15% | 4 |
| Niemann-Pick C1 protein precursor | 255652944 | 142 | 3 | 3.10% | 5 | 3 | 3.10% | 6 | 1 | 1.00% | 3 |
| stress-induced-phosphoprotein 1 isoform a | 544063423 | 68 | 3 | 5.60% | 8 | 2 | 3.40% | 4 | 6 | 13% | 14 |
| leucine-rich repeat-containing protein 37A3 precursor | 75677612 | 181 | 3 | 17% | 17 | 2 | 17% | 18 | 5 | 18% | 17 |
| probable Xaa-Pro aminopeptidase 3 isoform 1 | 11559925 | 57 | 3 | 12% | 12 | 2 | 8.90% | 6 | 5 | 15% | 11 |
| histidine triad nucleotide-binding protein 3 | 189217863 | 20 | 3 | 19% | 8 | 2 | 11% | 6 | 5 | 38% | 11 |
| sepiapterin reductase | 4507185 | 28 | 3 | 21% | 7 | 2 | 15% | 5 | 4 | 21% | 13 |
| mitochondrial inner membrane protease subunit 2 | 14211845 | 20 | 3 | 30% | 7 | 2 | 24% | 8 | 4 | 35% | 10 |
| cytochrome b-c1 complex subunit 6, mitochondrial | 83627705 | 11 | 3 | 48% | 15 | 2 | 48% | 9 | 3 | 58% | 9 |
| lanosterol synthase isoform X1 | 578836540 | 83 | 3 | 4.90% | 7 | 2 | 4.00% | 4 | 3 | 5.30% | 7 |
| 60S ribosomal protein L7a | 4506661 | 30 | 3 | 18% | 5 | 2 | 11% | 6 | 3 | 18% | 7 |
| fructose-bisphosphate aldolase C isoform X1 | 530410965 | 48 | 3 | 16% | 10 | 2 | 14% | 7 | 3 | 14% | 7 |
| CKLF-like MARVEL transmembrane domain-containing protein 1 isoform 13 | 148743783 | 31 | 3 | 11% | 7 | 2 | 8.00% | 6 | 3 | 11% | 8 |
| elongation factor 1-beta | 4503477 | 25 | 3 | 27% | 8 | 2 | 21% | 7 | 3 | 27% | 7 |
| dnaJ homolog subfamily A member 2 | 5031741 | 46 | 3 | 14% | 8 | 2 | 9.70% | 5 | 3 | 11% | 5 |
| probable proline--tRNA ligase, mitochondrial precursor | 34303926 | 53 | 3 | 14% | 7 | 2 | 6.70% | 3 | 3 | 10% | 4 |
| endoplasmic reticulum aminopeptidase 1 isoform X1 | 530380009 | 108 | 3 | 4.30% | 4 | 2 | 2.90% | 5 | 3 | 4.30% | 7 |
| succinyl-CoA ligase [GDP-forming] subunit beta, mitochondrial isoform 2 precursor | 157779135 | 47 | 3 | 12% | 8 | 2 | 7.40% | 2 | 3 | 7.40% | 4 |
| zona pellucida-binding protein 2 isoform 1 precursor | 84875535 | 36 | 3 | 16% | 11 | 2 | 10% | 6 | 2 | 10% | 10 |
| protein lunapark isoform X3 | 530371010 | 43 | 3 | 6.80% | 8 | 2 | 9.40% | 9 | 2 | 9.40% | 10 |
| SUN domain-containing protein 3 isoform 1 | 71834868 | 41 | 3 | 16% | 5 | 2 | 12% | 3 | 2 | 14% | 5 |
| AP-2 complex subunit alpha-2 isoform 2 | 27477041 | 104 | 3 | 6.20% | 3 | 2 | 4.30% | 3 | 2 | 5.20% | 6 |
| trans-2-enoyl-CoA reductase, mitochondrial isoform X1 | 530361129 | 44 | 3 | 11% | 6 | 2 | 6.20% | 7 | 2 | 8.20% | 9 |
| testis anion transporter 1 isoform X1 | 578811424 | 117 | 3 | 2.80% | 7 | 2 | 2.80% | 6 | 2 | 2.80% | 6 |
| atlastin-1 isoform b | 189181742 | 63 | 3 | 7.40% | 7 | 2 | 6.10% | 6 | 2 | 6.10% | 5 |
| arginine--tRNA ligase, cytoplasmic | 15149476 | 75 | 3 | 8.20% | 7 | 2 | 6.10% | 5 | 2 | 6.10% | 5 |
| eukaryotic translation elongation factor 1 epsilon-1 isoform 2 | 208879470 | 16 | 3 | 33% | 4 | 2 | 26% | 4 | 2 | 26% | 4 |
| GMP reductase 1 | 156104880 | 37 | 3 | 9.60% | 8 | 2 | 7.50% | 5 | 1 | 6.40% | 3 |
| voltage-dependent anion-selective channel protein 2 isoform 1 | 296317337 | 33 | 3 | 9.70% | 7 | 2 | 10% | 5 | 1 | 3.90% | 1 |
| myeloblastin precursor | 71361688 | 28 | 3 | 24% | 5 | 2 | 20% | 5 | 1 | 9.40% | 3 |
| aspartate--tRNA ligase, mitochondrial | 40789249 | 74 | 3 | 7.90% | 6 | 2 | 6.00% | 2 | ni | ni | ni |
| acyl carrier protein, mitochondrial precursor | 4826852 | 17 | 3 | 15% | 5 | 1 | 9.60% | 3 | 3 | 25% | 5 |
| transmembrane protein 209 | 66348165 | 63 | 3 | 12% | 6 | 1 | 4.60% | 3 | 3 | 9.60% | 5 |
| F-actin-capping protein subunit beta isoform 1 | 4826659 | 31 | 3 | 11% | 7 | 1 | 3.30% | 3 | 2 | 5.10% | 4 |
| izumo sperm-egg fusion protein 4 isoform X2 | 530425083 | 29 | 3 | 44% | 22 | 1 | 41% | 12 | 2 | 44% | 15 |
| haloacid dehalogenase-like hydrolase domain-containing protein 2 isoform X1 | 530413997 | 29 | 3 | 19% | 6 | 1 | 8.10% | 7 | 2 | 15% | 8 |
| EF-hand calcium-binding domain-containing protein 14 | 7662160 | 55 | 3 | 10% | 7 | 1 | 3.40% | 3 | 2 | 7.70% | 5 |
| 60S ribosomal protein L10a | 15431288 | 25 | 3 | 17% | 8 | 1 | 6.00% | 2 | 2 | 9.70% | 7 |
| nucleoporin Nup37 isoform X1 | 530400822 | 37 | 3 | 15% | 9 | 1 | 4.90% | 4 | 2 | 8.30% | 4 |
| annexin A6 isoform X2 | 530380520 | 75 | 3 | 6.40% | 9 | 1 | 2.40% | 2 | 1 | 2.40% | 1 |
| FAS-associated factor 2 | 24797106 | 53 | 3 | 10% | 6 | 1 | 6.50% | 1 | 1 | 6.50% | 1 |
| tubulin polymerization-promoting protein family member 2 | 226491350 | 19 | 3 | 32% | 5 | ni | ni | ni | 1 | 7.60% | 3 |
| mesothelin isoform X1 | 530407442 | 68 | 2 | 4.00% | 4 | 7 | 8.70% | 14 | 3 | 3.50% | 5 |
| UDP-glucose:glycoprotein glucosyltransferase 2 precursor | 238859593 | 175 | 2 | 2.20% | 2 | 6 | 7.60% | 17 | 8 | 9.20% | 15 |
| synaptogyrin-2 | 4759202 | 25 | 2 | 8.00% | 12 | 6 | 22% | 19 | 3 | 13% | 14 |
| mitochondrial carnitine/acylcarnitine carrier protein | 4557403 | 33 | 2 | 5.30% | 2 | 5 | 15% | 8 | 5 | 21% | 11 |
| isocitrate dehydrogenase [NAD] subunit gamma, mitochondrial isoform a precursor | 4758582 | 43 | 2 | 8.70% | 6 | 5 | 20% | 16 | 5 | 20% | 12 |
| 26S protease regulatory subunit 8 isoform 1 | 24497435 | 46 | 2 | 9.10% | 7 | 5 | 20% | 13 | 5 | 22% | 16 |
| reticulon-4 isoform A | 24431935 | 130 | 2 | 1.20% | 5 | 5 | 6.00% | 10 | 3 | 3.10% | 8 |
| protein FAM3C precursor | 91807125 | 25 | 2 | 13% | 5 | 5 | 25% | 13 | 2 | 13% | 6 |
| glycerol kinase 2 | 41393575 | 61 | 2 | 4.50% | 4 | 4 | 12% | 10 | 6 | 18% | 12 |
| ras-related protein Rab-1A isoform 1 | 4758988 | 23 | 2 | 20% | 3 | 4 | 38% | 16 | 4 | 29% | 9 |
| eukaryotic translation initiation factor 3 subunit K | 10801345 | 25 | 2 | 16% | 9 | 4 | 33% | 13 | 4 | 29% | 14 |
| C-Myc-binding protein | 57242777 | 12 | 2 | 30% | 7 | 4 | 55% | 13 | 4 | 55% | 13 |
| mitochondrial import receptor subunit TOM34 | 21361356 | 35 | 2 | 11% | 6 | 4 | 21% | 9 | 4 | 19% | 9 |
| 40S ribosomal protein S19 | 4506695 | 16 | 2 | 17% | 4 | 4 | 29% | 10 | 4 | 28% | 9 |
| dynactin subunit 4 isoform a | 208431793 | 53 | 2 | 9.90% | 6 | 4 | 16% | 9 | 4 | 13% | 8 |
| alpha-soluble NSF attachment protein | 47933379 | 33 | 2 | 4.40% | 4 | 4 | 19% | 6 | 3 | 18% | 7 |
| serine/threonine-protein phosphatase 2A 65 regulatory subunit A alpha isoform | 21361399 | 65 | 2 | 6.80% | 5 | 4 | 13% | 7 | 3 | 10% | 12 |
| short/branched chain specific acyl-CoA dehydrogenase, mitochondrial precursor | 4501859 | 47 | 2 | 7.60% | 4 | 4 | 16% | 10 | 3 | 12% | 11 |
| ribonuclease 4 precursor | 4506557 | 17 | 2 | 9.50% | 8 | 4 | 22% | 15 | 3 | 21% | 13 |
| leucine-rich repeat-containing protein 59 | 40254924 | 35 | 2 | 12% | 5 | 4 | 25% | 10 | 2 | 12% | 4 |
| solute carrier family 25 member 35 isoform X1 | 530410311 | 32 | 2 | 8.70% | 4 | 3 | 12% | 9 | 5 | 22% | 11 |
| dihydroxyacetone phosphate acyltransferase isoform X1 | 530366583 | 77 | 2 | 4.60% | 4 | 3 | 6.90% | 6 | 5 | 16% | 10 |
| sperm acrosome membrane-associated protein 4 precursor | 19424138 | 13 | 2 | 22% | 29 | 3 | 38% | 38 | 4 | 35% | 30 |
| monocarboxylate transporter 1 | 115583685 | 54 | 2 | 6.40% | 11 | 3 | 11% | 7 | 4 | 11% | 10 |
| fatty acid-binding protein, epidermal | 4557581 | 15 | 2 | 19% | 5 | 3 | 33% | 7 | 4 | 39% | 8 |
| cob(I)yrinic acid a,c-diamide adenosyltransferase, mitochondrial precursor | 16418349 | 27 | 2 | 10% | 5 | 3 | 18% | 12 | 4 | 22% | 10 |
| 26S protease regulatory subunit 6A | 21361144 | 49 | 2 | 5.70% | 5 | 3 | 9.30% | 7 | 4 | 13% | 6 |
| 6-phosphogluconolactonase | 6912586 | 28 | 2 | 14% | 3 | 3 | 29% | 7 | 4 | 38% | 6 |
| translin-associated protein X | 5174731 | 33 | 2 | 13% | 10 | 3 | 16% | 15 | 3 | 17% | 15 |
| protein S100-A11 | 5032057 | 12 | 2 | 46% | 8 | 3 | 56% | 10 | 3 | 56% | 9 |
| endoplasmic reticulum-Golgi intermediate compartment protein 2 | 50959176 | 43 | 2 | 9.30% | 6 | 3 | 13% | 6 | 3 | 13% | 8 |
| high mobility group protein B4 | 57232750 | 22 | 2 | 17% | 7 | 3 | 22% | 9 | 3 | 22% | 9 |
| fructose-1,6-bisphosphatase 1 | 16579888 | 37 | 2 | 8.30% | 8 | 3 | 13% | 8 | 3 | 13% | 6 |
| antileukoproteinase precursor | 4507065 | 14 | 2 | 15% | 10 | 3 | 33% | 10 | 3 | 27% | 13 |
| ribose-phosphate pyrophosphokinase 2 isoform 2 | 4506129 | 35 | 2 | 6.90% | 5 | 3 | 12% | 5 | 3 | 15% | 11 |
| 26S proteasome non-ATPase regulatory subunit 12 isoform 1 | 4506221 | 53 | 2 | 9.60% | 10 | 3 | 12% | 7 | 3 | 7.90% | 11 |
| glutamine synthetase isoform X1 | 578800828 | 42 | 2 | 9.90% | 5 | 3 | 12% | 8 | 3 | 10% | 6 |
| syntenin-1 isoform 1 | 55749490 | 32 | 2 | 15% | 9 | 3 | 18% | 10 | 3 | 18% | 11 |
| glycine cleavage system H protein, mitochondrial precursor | 49574537 | 19 | 2 | 17% | 4 | 3 | 39% | 13 | 3 | 39% | 8 |
| 60S acidic ribosomal protein P1 isoform 1 | 4506669 | 12 | 2 | 52% | 4 | 3 | 80% | 10 | 3 | 80% | 7 |
| dolichyl-diphosphooligosaccharide--protein glycosyltransferase subunit DAD1 | 4503253 | 12 | 2 | 11% | 6 | 3 | 28% | 12 | 3 | 19% | 8 |
| tumor protein p53-inducible protein 11 | 33695117 | 21 | 2 | 14% | 6 | 3 | 14% | 8 | 3 | 14% | 7 |
| mitoferrin-1 | 82775373 | 37 | 2 | 13% | 4 | 3 | 23% | 6 | 3 | 22% | 7 |
| vesicle-associated membrane protein-associated protein B/C isoform 1 | 4759302 | 27 | 2 | 17% | 5 | 3 | 18% | 4 | 3 | 18% | 9 |
| dehydrogenase/reductase SDR family member 11 precursor | 109715829 | 28 | 2 | 10% | 3 | 3 | 15% | 9 | 3 | 16% | 7 |
| leukocyte surface antigen CD47 isoform 2 precursor | 38683836 | 33 | 2 | 6.20% | 7 | 3 | 9.20% | 13 | 2 | 6.20% | 10 |
| CD63 antigen isoform A | 383872447 | 26 | 2 | 7.60% | 15 | 3 | 7.60% | 14 | 2 | 7.60% | 17 |
| alpha-actinin-1 isoform b | 4501891 | 103 | 2 | 14% | 4 | 3 | 15% | 7 | 2 | 16% | 4 |
| serine/threonine-protein phosphatase 2A catalytic subunit alpha isoform | 4506017 | 36 | 2 | 13% | 3 | 3 | 16% | 7 | 2 | 13% | 4 |
| acetyl-CoA acetyltransferase, cytosolic | 148539872 | 41 | 2 | 14% | 6 | 3 | 17% | 5 | 2 | 13% | 6 |
| aflatoxin B1 aldehyde reductase member 2 | 41327764 | 40 | 2 | 9.50% | 10 | 3 | 13% | 9 | 2 | 9.50% | 8 |
| sulfatase-modifying factor 2 isoform e precursor | 194248090 | 39 | 2 | 6.10% | 6 | 3 | 8.10% | 7 | 2 | 7.50% | 6 |
| actin-related protein T3 | 221139714 | 41 | 2 | 12% | 6 | 3 | 15% | 6 | 2 | 12% | 6 |
| glutathione S-transferase omega-1 isoform 1 | 4758484 | 28 | 2 | 19% | 5 | 3 | 25% | 6 | 2 | 19% | 5 |
| D-dopachrome decarboxylase isoform X1 | 578840688 | 23 | 2 | 11% | 6 | 3 | 17% | 11 | 2 | 11% | 8 |
| myosin regulatory light chain 12A isoform X1 | 530424789 | 20 | 2 | 12% | 5 | 3 | 19% | 6 | 2 | 12% | 6 |
| thioredoxin reductase 1, cytoplasmic isoform 1 | 148277065 | 60 | 2 | 11% | 8 | 3 | 18% | 7 | 2 | 13% | 5 |
| UPF0587 protein C1orf123 | 8923541 | 18 | 2 | 25% | 2 | 3 | 35% | 5 | 2 | 25% | 3 |
| dynein light chain roadblock-type 2 | 18702323 | 11 | 2 | 29% | 4 | 3 | 51% | 9 | 2 | 29% | 6 |
| polyadenylate-binding protein 1 isoform X1 | 530388906 | 71 | 2 | 4.70% | 3 | 3 | 7.50% | 5 | 2 | 4.90% | 4 |
| immunoglobulin-binding protein 1 | 4557663 | 39 | 2 | 6.50% | 6 | 3 | 13% | 7 | 2 | 6.50% | 6 |
| carbonic anhydrase 6 isoform 1 precursor | 70167127 | 35 | 2 | 10% | 5 | 3 | 18% | 7 | 2 | 17% | 7 |
| calcineurin B homologous protein 1 | 6005731 | 22 | 2 | 14% | 3 | 3 | 22% | 4 | 2 | 14% | 2 |
| zinc transporter ZIP3 isoform a | 32490561 | 34 | 2 | 9.60% | 4 | 3 | 20% | 8 | 1 | 7.00% | 5 |
| inactive serine protease 54 precursor | 122937420 | 44 | 2 | 7.10% | 6 | 3 | 12% | 14 | 1 | 4.60% | 6 |
| pyruvate dehydrogenase kinase, isozyme 3 isoform 1 precursor | 215422338 | 48 | 2 | 9.90% | 4 | 3 | 11% | 5 | 1 | 5.80% | 1 |
| thymidylate kinase isoform 1 | 42544174 | 24 | 2 | 17% | 5 | 3 | 20% | 6 | 1 | 5.70% | 1 |
| carbonyl reductase [NADPH] 1 isoform 1 | 4502599 | 30 | 2 | 15% | 4 | 3 | 21% | 5 | 1 | 11% | 3 |
| annexin A2 isoform 2 | 50845386 | 39 | 2 | 9.40% | 8 | 2 | 12% | 7 | 4 | 12% | 10 |
| protein disulfide-isomerase-like protein of the testis precursor | 28372543 | 67 | 2 | 3.40% | 3 | 2 | 4.30% | 5 | 4 | 8.00% | 12 |
| protein FAM162A | 49355721 | 17 | 2 | 20% | 12 | 2 | 20% | 17 | 3 | 25% | 18 |
| histone H2B type F-S-like | 578836330 | 14 | 2 | 40% | 11 | 2 | 41% | 14 | 3 | 41% | 11 |
| histone H3.3 | 4885385 | 15 | 2 | 29% | 8 | 2 | 29% | 13 | 3 | 35% | 9 |
| 14-3-3 protein sigma | 5454052 | 28 | 2 | 17% | 9 | 2 | 17% | 9 | 3 | 27% | 8 |
| ferritin heavy chain | 56682959 | 21 | 2 | 19% | 4 | 2 | 19% | 2 | 3 | 26% | 5 |
| programmed cell death 6-interacting protein isoform 1 | 22027538 | 96 | 2 | 5.90% | 6 | 2 | 5.90% | 6 | 3 | 8.60% | 8 |
| nucleosome assembly protein 1-like 1 | 21327708 | 45 | 2 | 7.20% | 6 | 2 | 7.20% | 6 | 3 | 9.70% | 9 |
| uncharacterized protein C19orf18 precursor | 22748999 | 24 | 2 | 18% | 7 | 2 | 18% | 6 | 3 | 24% | 9 |
| V-type proton ATPase subunit G 1 | 4757818 | 14 | 2 | 29% | 7 | 2 | 31% | 6 | 3 | 31% | 8 |
| 60S ribosomal protein L14 | 78000183 | 23 | 2 | 11% | 6 | 2 | 11% | 7 | 3 | 16% | 7 |
| vitronectin precursor | 88853069 | 54 | 2 | 5.20% | 6 | 2 | 5.20% | 4 | 3 | 5.20% | 8 |
| ganglioside-induced differentiation-associated protein 1 isoform a | 108773797 | 41 | 2 | 9.50% | 5 | 2 | 9.50% | 6 | 3 | 15% | 8 |
| GPI transamidase component PIG-S | 15088795 | 62 | 2 | 9.40% | 6 | 2 | 9.40% | 9 | 3 | 12% | 10 |
| isobutyryl-CoA dehydrogenase, mitochondrial isoform X1 | 530397985 | 47 | 2 | 8.50% | 6 | 2 | 8.50% | 5 | 3 | 7.60% | 6 |
| chloride intracellular channel protein 1 | 14251209 | 27 | 2 | 19% | 5 | 2 | 19% | 5 | 3 | 24% | 7 |
| adenylate kinase 7 | 148727333 | 83 | 2 | 4.30% | 5 | 2 | 4.30% | 5 | 3 | 6.50% | 7 |
| quinone oxidoreductase isoform a | 13236495 | 35 | 2 | 12% | 5 | 2 | 16% | 4 | 3 | 19% | 10 |
| beta-microseminoprotein isoform a precursor | 4557036 | 13 | 2 | 8.80% | 11 | 2 | 8.80% | 7 | 2 | 23% | 12 |
| vitamin D-binding protein isoform 3 precursor | 324021745 | 55 | 2 | 5.30% | 5 | 2 | 5.30% | 9 | 2 | 5.30% | 10 |
| ATP synthase subunit f, mitochondrial isoform 2a | 4757812 | 11 | 2 | 26% | 18 | 2 | 26% | 19 | 2 | 26% | 18 |
| succinate dehydrogenase cytochrome b560 subunit, mitochondrial isoform 1 precursor | 4506863 | 19 | 2 | 12% | 4 | 2 | 12% | 5 | 2 | 24% | 6 |
| glutamate dehydrogenase 1, mitochondrial precursor | 4885281 | 61 | 2 | 6.60% | 5 | 2 | 7.90% | 6 | 2 | 7.90% | 4 |
| lysozyme-like protein 2 | 73088987 | 22 | 2 | 20% | 12 | 2 | 20% | 13 | 2 | 20% | 18 |
| myosin light polypeptide 6 isoform 1 | 17986258 | 17 | 2 | 32% | 2 | 2 | 36% | 9 | 2 | 32% | 3 |
| tubulin alpha-1B chain | 57013276 | 50 | 2 | 51% | 5 | 2 | 56% | 15 | 2 | 65% | 15 |
| pituitary tumor-transforming gene 1 protein-interacting protein isoform 1 precursor | 4757886 | 20 | 2 | 17% | 8 | 2 | 17% | 15 | 2 | 17% | 9 |
| tubulin alpha-1A chain isoform 1 | 17986283 | 50 | 2 | 54% | 13 | 2 | 56% | 9 | 2 | 65% | 12 |
| leucine-rich repeat-containing protein 37A isoform X2 | 530413286 | 184 | 2 | 17% | 9 | 2 | 18% | 14 | 2 | 18% | 12 |
| immunoglobulin lambda-like polypeptide 5 isoform 1 | 295986608 | 23 | 2 | 16% | 9 | 2 | 16% | 14 | 2 | 16% | 8 |
| protein S100-A8 | 21614544 | 11 | 2 | 19% | 7 | 2 | 19% | 5 | 2 | 19% | 8 |
| fumarylacetoacetate hydrolase domain-containing protein 2A | 156231349 | 35 | 2 | 40% | 3 | 2 | 36% | 7 | 2 | 40% | 8 |
| WAP four-disulfide core domain protein 2 precursor | 56699495 | 13 | 2 | 21% | 6 | 2 | 21% | 7 | 2 | 21% | 12 |
| monocyte differentiation antigen CD14 precursor | 291575163 | 40 | 2 | 9.10% | 4 | 2 | 9.90% | 4 | 2 | 9.90% | 5 |
| 40S ribosomal protein S15 | 4506687 | 17 | 2 | 34% | 7 | 2 | 34% | 11 | 2 | 34% | 9 |
| pyruvate kinase PKM isoform X3 | 530405979 | 58 | 2 | 61% | 4 | 2 | 58% | 7 | 2 | 63% | 8 |
| cytochrome c oxidase subunit 7A2, mitochondrial precursor | 262118227 | 13 | 2 | 32% | 8 | 2 | 32% | 10 | 2 | 32% | 8 |
| dnaJ homolog subfamily C member 5B | 29126218 | 22 | 2 | 16% | 6 | 2 | 17% | 6 | 2 | 16% | 6 |
| cytochrome c oxidase protein 20 homolog | 37620210 | 13 | 2 | 31% | 6 | 2 | 31% | 5 | 2 | 31% | 6 |
| D-beta-hydroxybutyrate dehydrogenase, mitochondrial isoform X4 | 530375922 | 23 | 2 | 17% | 3 | 2 | 17% | 3 | 2 | 17% | 7 |
| estradiol 17-beta-dehydrogenase 8 | 15277342 | 27 | 2 | 10% | 5 | 2 | 9.20% | 6 | 2 | 10% | 4 |
| prostaglandin E synthase 2 isoform 1 | 13376617 | 42 | 2 | 12% | 5 | 2 | 8.80% | 5 | 2 | 12% | 6 |
| protein FAM209B isoform X1 | 530418105 | 27 | 2 | 10% | 7 | 2 | 10% | 8 | 2 | 10% | 9 |
| signal peptidase complex catalytic subunit SEC11C isoform X1 | 530414371 | 18 | 2 | 19% | 8 | 2 | 19% | 7 | 2 | 19% | 8 |
| cytochrome c oxidase subunit 6A1, mitochondrial | 17999528 | 12 | 2 | 43% | 3 | 2 | 43% | 7 | 2 | 43% | 6 |
| translation initiation factor eIF-2B subunit beta | 7657058 | 39 | 2 | 15% | 6 | 2 | 15% | 6 | 2 | 15% | 7 |
| aquaporin-5 | 4502183 | 28 | 2 | 8.70% | 6 | 2 | 8.70% | 6 | 2 | 8.70% | 9 |
| inositol-3-phosphate synthase 1 isoform X1 | 578833308 | 82 | 2 | 4.40% | 3 | 2 | 4.00% | 6 | 2 | 4.00% | 6 |
| 60S ribosomal protein L19 | 4506609 | 23 | 2 | 13% | 5 | 2 | 13% | 6 | 2 | 13% | 5 |
| mitochondrial ornithine transporter 1 | 7657585 | 33 | 2 | 15% | 4 | 2 | 15% | 4 | 2 | 15% | 4 |
| DCN1-like protein 1 | 36030883 | 30 | 2 | 9.70% | 7 | 2 | 9.70% | 6 | 2 | 9.70% | 9 |
| myeloid-associated differentiation marker | 19923903 | 35 | 2 | 10% | 6 | 2 | 10% | 5 | 2 | 10% | 6 |
| pleiotrophin precursor | 4506281 | 19 | 2 | 24% | 4 | 2 | 24% | 5 | 2 | 24% | 5 |
| prostaglandin E synthase | 4758910 | 17 | 2 | 22% | 7 | 2 | 16% | 6 | 2 | 22% | 5 |
| profilin-1 | 4826898 | 15 | 2 | 20% | 4 | 2 | 20% | 4 | 2 | 20% | 4 |
| bifunctional purine biosynthesis protein PURH isoform X1 | 578804376 | 59 | 2 | 11% | 5 | 2 | 11% | 6 | 2 | 7.70% | 5 |
| phosphoglucomutase-1 isoform 1 | 21361621 | 61 | 2 | 9.80% | 6 | 2 | 9.80% | 6 | 2 | 9.80% | 7 |
| spermatogenesis-associated protein 31D1 | 48717285 | 176 | 2 | 1.90% | 3 | 2 | 1.90% | 2 | 2 | 1.90% | 5 |
| protein CYR61 precursor | 31542331 | 42 | 2 | 7.10% | 5 | 2 | 12% | 4 | 2 | 7.10% | 4 |
| GTP:AMP phosphotransferase AK3, mitochondrial isoform a | 19923437 | 26 | 2 | 13% | 4 | 2 | 14% | 5 | 2 | 14% | 4 |
| WNT1-inducible-signaling pathway protein 2 precursor | 4507923 | 27 | 2 | 12% | 5 | 2 | 12% | 4 | 2 | 18% | 2 |
| ribosome-binding protein 1 | 110611218 | 109 | 2 | 2.70% | 4 | 2 | 4.60% | 3 | 2 | 2.40% | 5 |
| 60S ribosomal protein L24 | 4506619 | 18 | 2 | 13% | 3 | 2 | 11% | 6 | 2 | 13% | 5 |
| PH and SEC7 domain-containing protein 3 isoform a | 117606360 | 116 | 2 | 4.00% | 4 | 2 | 4.00% | 4 | 2 | 3.70% | 2 |
| unconventional myosin-VI isoform X2 | 530383144 | 149 | 2 | 2.30% | 4 | 2 | 1.20% | 4 | 1 | 1.20% | 2 |
| iron-sulfur cluster co-chaperone protein HscB, mitochondrial precursor | 50301234 | 27 | 2 | 13% | 5 | 2 | 15% | 4 | 1 | 4.30% | 2 |
| mitochondrial carrier homolog 2 | 7657347 | 33 | 2 | 15% | 3 | 2 | 15% | 7 | 1 | 7.90% | 6 |
| F-actin-capping protein subunit alpha-1 | 5453597 | 33 | 2 | 8.70% | 9 | 2 | 8.70% | 6 | 1 | 3.50% | 3 |
| protein O-linked-mannose beta-1,4-N-acetylglucosaminyltransferase 2 isoform X1 | 530373252 | 67 | 2 | 8.40% | 8 | 2 | 8.40% | 4 | 1 | 3.30% | 3 |
| NADH dehydrogenase [ubiquinone] 1 alpha subcomplex subunit 11 isoform 2 | 301129187 | 23 | 2 | 17% | 5 | 2 | 17% | 6 | 1 | 10% | 3 |
| 60S ribosomal protein L11 isoform 2 | 315221152 | 20 | 2 | 13% | 5 | 2 | 13% | 9 | 1 | 7.90% | 3 |
| trypsin-3 isoform 3 preproprotein | 342672030 | 28 | 2 | 5.00% | 7 | 2 | 8.80% | 6 | 1 | 5.00% | 6 |
| ras-related protein Rap-1b isoform 1 precursor | 58219792 | 21 | 2 | 13% | 5 | 2 | 7.60% | 4 | 1 | 6.50% | 3 |
| epididymal secretory protein E3-alpha precursor | 11386189 | 18 | 2 | 12% | 5 | 2 | 13% | 6 | 1 | 4.80% | 3 |
| glucosamine-6-phosphate isomerase 2 isoform X4 | 578808487 | 33 | 2 | 16% | 7 | 2 | 16% | 6 | 1 | 22% | 3 |
| spermatid-associated protein isoform 1 | 22749425 | 52 | 2 | 6.20% | 6 | 2 | 6.20% | 4 | 1 | 3.60% | 3 |
| 60S ribosomal protein L23a | 17105394 | 18 | 2 | 13% | 4 | 2 | 14% | 8 | 1 | 8.30% | 3 |
| calcium signal-modulating cyclophilin ligand | 4502559 | 33 | 2 | 9.80% | 4 | 2 | 9.80% | 5 | 1 | 6.80% | 3 |
| ethanolaminephosphotransferase 1 | 50083289 | 45 | 2 | 10% | 2 | 2 | 9.30% | 5 | 1 | 5.80% | 4 |
| presenilin-1 isoform I-463 | 195947397 | 52 | 2 | 7.30% | 3 | 2 | 7.30% | 5 | 1 | 4.10% | 1 |
| gelsolin isoform X1 | 530391018 | 83 | 2 | 5.10% | 2 | 2 | 3.60% | 2 | ni | ni | ni |
| alpha-1-antichymotrypsin precursor | 50659080 | 48 | 2 | 7.10% | 5 | 1 | 4.70% | 1 | 5 | 18% | 9 |
| ethanolamine-phosphate cytidylyltransferase isoform 1 | 296841136 | 46 | 2 | 9.10% | 6 | 1 | 5.40% | 5 | 4 | 14% | 11 |
| histone H1t | 20544168 | 22 | 2 | 11% | 6 | 1 | 5.30% | 1 | 3 | 15% | 7 |
| bifunctional epoxide hydrolase 2 isoform a | 27597073 | 63 | 2 | 7.60% | 9 | 1 | 3.80% | 6 | 3 | 10% | 12 |
| NADH dehydrogenase [ubiquinone] 1 beta subcomplex subunit 4 isoform 1 | 6041669 | 15 | 2 | 22% | 7 | 1 | 14% | 6 | 3 | 32% | 10 |
| protoporphyrinogen oxidase | 170650615 | 51 | 2 | 3.80% | 6 | 1 | 2.50% | 2 | 3 | 7.10% | 7 |
| nucleoporin NDC1 isoform 1 | 271398350 | 76 | 2 | 4.30% | 3 | 1 | 1.60% | 2 | 3 | 4.60% | 7 |
| syndecan-1 precursor | 29568086 | 32 | 2 | 5.50% | 4 | 1 | 5.50% | 3 | 3 | 5.50% | 5 |
| brain acid soluble protein 1 | 30795231 | 23 | 2 | 19% | 5 | 1 | 12% | 3 | 3 | 29% | 6 |
| magnesium transporter protein 1 | 215983058 | 42 | 2 | 5.40% | 4 | 1 | 2.70% | 3 | 3 | 7.90% | 8 |
| outer dense fiber protein 3 isoform 2 | 554790333 | 23 | 2 | 12% | 4 | 1 | 5.30% | 4 | 3 | 20% | 7 |
| 40S ribosomal protein SA | 59859885 | 33 | 2 | 15% | 4 | 1 | 4.40% | 3 | 3 | 14% | 7 |
| N-acetylated-alpha-linked acidic dipeptidase 2 isoform X2 | 530396166 | 77 | 2 | 5.10% | 3 | 1 | 2.30% | 2 | 3 | 5.50% | 4 |
| putative ATP-dependent Clp protease proteolytic subunit, mitochondrial precursor | 5174419 | 30 | 2 | 18% | 3 | 1 | 12% | 3 | 3 | 22% | 5 |
| 40S ribosomal protein S13 | 4506685 | 17 | 2 | 19% | 3 | 1 | 8.60% | 3 | 2 | 8.60% | 6 |
| 40S ribosomal protein S3a isoform 1 | 4506723 | 30 | 2 | 9.80% | 4 | 1 | 6.80% | 1 | 2 | 9.80% | 4 |
| tropomyosin alpha-3 chain isoform 2 | 24119203 | 29 | 2 | 9.70% | 4 | 1 | 5.60% | 3 | 2 | 10% | 4 |
| PRA1 family protein 3 | 5453704 | 22 | 2 | 16% | 5 | 1 | 10% | 6 | 2 | 16% | 5 |
| serine incorporator 3 precursor | 39812106 | 53 | 2 | 4.90% | 7 | 1 | 2.50% | 8 | 2 | 4.90% | 9 |
| macrophage migration inhibitory factor | 4505185 | 12 | 2 | 17% | 9 | 1 | 7.80% | 6 | 2 | 17% | 9 |
| solute carrier family 15 member 2 isoform a | 226371746 | 82 | 2 | 3.40% | 4 | 1 | 1.10% | 3 | 2 | 3.40% | 6 |
| beta-defensin 126 preproprotein | 13624333 | 12 | 2 | 9.00% | 6 | 1 | 8.10% | 4 | 2 | 9.00% | 6 |
| mitochondrial import inner membrane translocase subunit TIM50 | 48526509 | 50 | 2 | 4.60% | 4 | 1 | 2.20% | 3 | 2 | 5.70% | 5 |
| dnaJ homolog subfamily B member 1 | 5453690 | 38 | 2 | 10.00% | 6 | 1 | 5.30% | 3 | 2 | 8.80% | 7 |
| secretory carrier-associated membrane protein 4 | 17738287 | 26 | 2 | 10% | 4 | 1 | 4.80% | 3 | 2 | 10% | 4 |
| 26S proteasome non-ATPase regulatory subunit 5 isoform 1 | 4826952 | 56 | 2 | 5.60% | 4 | 1 | 4.20% | 3 | 2 | 6.00% | 4 |
| S-methyl-5'-thioadenosine phosphorylase | 47132622 | 31 | 2 | 14% | 2 | 1 | 8.10% | 2 | 2 | 14% | 5 |
| transmembrane protein 120A | 13994300 | 41 | 2 | 6.10% | 6 | 1 | 2.90% | 3 | 2 | 6.10% | 5 |
| legumain preproprotein | 56682962 | 49 | 2 | 4.60% | 4 | 1 | 4.60% | 3 | 2 | 4.60% | 4 |
| alpha-1,2-mannosyltransferase ALG9 isoform a | 118026921 | 71 | 2 | 5.00% | 4 | 1 | 2.60% | 3 | 2 | 5.00% | 6 |
| signal peptide peptidase-like 2B isoform 3 precursor | 116734691 | 56 | 2 | 3.50% | 4 | 1 | 2.20% | 3 | 2 | 3.50% | 6 |
| transcription elongation factor B polypeptide 1 isoform X1 | 530388540 | 12 | 2 | 30% | 4 | 1 | 18% | 3 | 2 | 29% | 6 |
| 60S ribosomal protein L15 isoform 1 | 15431293 | 24 | 2 | 13% | 4 | 1 | 5.90% | 1 | 2 | 9.30% | 4 |
| transaldolase | 5803187 | 38 | 2 | 8.00% | 4 | 1 | 3.30% | 2 | 2 | 8.00% | 3 |
| nuclear pore complex-interacting protein family member B9 isoform X1 | 530409579 | 50 | 2 | 6.20% | 4 | 1 | 3.90% | 3 | 2 | 6.00% | 2 |
| ropporin-1-like protein | 17572807 | 26 | 2 | 10% | 2 | 1 | 4.80% | 2 | 2 | 10% | 2 |
| beta-defensin 129 precursor | 18250304 | 20 | 2 | 16% | 3 | 1 | 3.80% | 1 | 1 | 3.80% | 2 |
| tripeptidyl-peptidase 1 preproprotein | 5729770 | 61 | 2 | 8.00% | 8 | 1 | 4.80% | 4 | 1 | 4.80% | 6 |
| torsin-1A-interacting protein 1 isoform 1 | 389886539 | 66 | 2 | 4.50% | 6 | 1 | 2.10% | 3 | 1 | 2.40% | 4 |
| heat shock protein 105 isoform X3 | 578824728 | 103 | 2 | 4.60% | 4 | 1 | 3.00% | 3 | 1 | 3.00% | 3 |
| regenerating islet-derived protein 3-gamma isoform X1 | 530367218 | 19 | 2 | 18% | 6 | 1 | 8.00% | 2 | 1 | 10% | 3 |
| lanC-like protein 1 isoform X1 | 530369695 | 46 | 2 | 10% | 5 | 1 | 5.40% | 2 | 1 | 5.40% | 3 |
| syntaxin-8 | 4759188 | 27 | 2 | 14% | 7 | 1 | 6.40% | 3 | 1 | 7.60% | 6 |
| coatomer subunit gamma-1 | 11559929 | 98 | 2 | 3.30% | 3 | 1 | 1.40% | 3 | 1 | 1.40% | 2 |
| bleomycin hydrolase | 4557367 | 53 | 2 | 8.10% | 5 | 1 | 2.40% | 3 | 1 | 2.40% | 3 |
| branched-chain-amino-acid aminotransferase, mitochondrial isoform b | 258614015 | 34 | 2 | 11% | 4 | 1 | 5.30% | 3 | 1 | 5.30% | 3 |
| NAD kinase 2, mitochondrial isoform X1 | 530378682 | 52 | 2 | 5.60% | 5 | 1 | 3.00% | 3 | 1 | 3.00% | 3 |
| nucleosome assembly protein 1-like 4 | 5174613 | 43 | 2 | 6.90% | 3 | 1 | 2.90% | 3 | 1 | 5.60% | 3 |
| transmembrane protein 191C | 333440473 | 39 | 2 | 14% | 4 | 1 | 6.60% | 4 | 1 | 6.60% | 3 |
| ferritin light chain | 20149498 | 20 | 2 | 18% | 4 | 1 | 8.60% | 3 | 1 | 9.10% | 3 |
| NAD-dependent protein deacetylase sirtuin-3, mitochondrial isoform X1 | 530395167 | 46 | 2 | 12% | 4 | 1 | 2.60% | 3 | 1 | 2.60% | 3 |
| dipeptidase 2 isoform X1 | 578829118 | 53 | 2 | 9.70% | 5 | 1 | 6.40% | 3 | 1 | 6.40% | 3 |
| reticulocalbin-1 precursor | 4506455 | 39 | 2 | 8.50% | 2 | 1 | 4.80% | 3 | 1 | 4.80% | 3 |
| importin subunit alpha-7 isoform X1 | 530362302 | 63 | 2 | 5.70% | 4 | 1 | 3.20% | 3 | 1 | 3.20% | 3 |
| phenylalanine--tRNA ligase beta subunit | 124028525 | 66 | 2 | 5.60% | 2 | 1 | 1.90% | 1 | 1 | 3.70% | 1 |
| coiled-coil domain-containing protein 151 | 117553613 | 69 | 2 | 4.50% | 5 | 1 | 2.00% | 1 | 1 | 2.00% | 1 |
| tetranectin precursor | 156627579 | 23 | 2 | 16% | 3 | 1 | 10% | 3 | 1 | 5.90% | 3 |
| prosalusin isoform a precursor | 145386578 | 36 | 2 | 9.00% | 3 | 1 | 4.00% | 2 | 1 | 4.00% | 1 |
| beta-actin-like protein 2 | 63055057 | 42 | 2 | 20% | 5 | 1 | 15% | 1 | ni | ni | ni |
| proteasomal ubiquitin receptor ADRM1 isoform 1 | 28373192 | 42 | 2 | 8.60% | 3 | 1 | 4.90% | 2 | ni | ni | ni |
| ADP-dependent glucokinase precursor | 31542509 | 54 | 2 | 13% | 6 | ni | ni | ni | 2 | 9.90% | 6 |
| L-lactate dehydrogenase A-like 6A isoform X1 | 578820358 | 39 | 2 | 14% | 4 | ni | ni | ni | 2 | 10% | 4 |
| carcinoembryonic antigen-related cell adhesion molecule 1 isoform 1 precursor | 19923195 | 58 | 2 | 4.90% | 4 | ni | ni | ni | 1 | 1.70% | 3 |
| ATP synthase subunit e, mitochondrial | 6005717 | 8 | 2 | 35% | 4 | ni | ni | ni | 1 | 20% | 3 |
| polypeptide N-acetylgalactosaminyltransferase 3 isoform X1 | 530370133 | 73 | 2 | 5.70% | 5 | ni | ni | ni | 1 | 2.10% | 2 |
| neuroplastin isoform c precursor | 238624147 | 44 | 2 | 7.90% | 3 | ni | ni | ni | 1 | 4.60% | 2 |
| nuclear protein localization protein 4 homolog | 157426879 | 68 | 2 | 8.70% | 4 | ni | ni | ni | 1 | 2.30% | 1 |
| ankyrin repeat domain-containing protein 17 isoform a | 38683807 | 274 | 2 | 2.50% | 2 | ni | ni | ni | ni | ni | ni |
| citrate lyase subunit beta-like protein, mitochondrial isoform X1 | 530423310 | 37 | 2 | 12% | 6 | ni | ni | ni | ni | ni | ni |
| nuclear migration protein nudC | 5729953 | 38 | 2 | 9.10% | 5 | ni | ni | ni | ni | ni | ni |
| coiled-coil domain-containing protein 47 isoform X1 | 530412564 | 56 | 2 | 5.40% | 3 | ni | ni | ni | ni | ni | ni |
| V-type proton ATPase subunit C 1 | 4502315 | 44 | 2 | 11% | 2 | ni | ni | ni | ni | ni | ni |
| mannose-6-phosphate isomerase isoform 4 | 574584840 | 40 | 2 | 8.60% | 3 | ni | ni | ni | ni | ni | ni |
| cysteine-rich secretory protein LCCL domain-containing 2 precursor | 13899332 | 56 | 1 | 3.80% | 3 | 5 | 17% | 13 | 4 | 13% | 13 |
| V-type proton ATPase 116 subunit a isoform 1 isoform b | 194097403 | 96 | 1 | 1.60% | 3 | 5 | 7.90% | 10 | 3 | 2.60% | 7 |
| glutathione synthetase isoform X2 | 530418095 | 52 | 1 | 4.00% | 5 | 4 | 13% | 12 | 5 | 16% | 17 |
| ras-related protein Rab-4B | 82659107 | 24 | 1 | 17% | 3 | 4 | 38% | 10 | 3 | 29% | 6 |
| annexin A1 | 4502101 | 39 | 1 | 6.90% | 1 | 3 | 15% | 6 | 4 | 19% | 7 |
| surfeit locus protein 4 isoform 1 | 19557691 | 30 | 1 | 3.70% | 1 | 3 | 15% | 11 | 3 | 15% | 8 |
| coiled-coil-helix-coiled-coil-helix domain-containing protein 6, mitochondrial | 14150134 | 26 | 1 | 6.00% | 3 | 3 | 24% | 9 | 3 | 24% | 5 |
| actin-related protein 2/3 complex subunit 2 | 23238211 | 34 | 1 | 9.30% | 2 | 3 | 18% | 7 | 2 | 16% | 9 |
| dehydrogenase/reductase SDR family member 7B isoform X1 | 578829832 | 40 | 1 | 3.80% | 3 | 3 | 7.80% | 8 | 2 | 3.80% | 2 |
| mannose-P-dolichol utilization defect 1 protein isoform X2 | 578830102 | 28 | 1 | 3.90% | 2 | 3 | 13% | 6 | 2 | 7.70% | 3 |
| SPARC-related modular calcium-binding protein 1 isoform 2 precursor | 11545873 | 48 | 1 | 4.60% | 6 | 3 | 9.20% | 13 | 2 | 6.50% | 9 |
| cysteine-rich secretory protein 3 isoform 1 precursor | 300244560 | 29 | 1 | 6.20% | 2 | 3 | 22% | 6 | 2 | 18% | 5 |
| lysophospholipase-like protein 1 | 20270341 | 26 | 1 | 4.20% | 2 | 3 | 17% | 6 | 2 | 13% | 2 |
| phosphatidylethanolamine-binding protein 4 precursor | 116812622 | 26 | 1 | 12% | 3 | 3 | 26% | 6 | 2 | 22% | 4 |
| mitochondrial peptide methionine sulfoxide reductase isoform c | 208609995 | 22 | 1 | 7.80% | 3 | 3 | 26% | 7 | 1 | 7.80% | 6 |
| sodium-dependent phosphate transport protein 2B isoform a | 110611906 | 76 | 1 | 1.60% | 1 | 3 | 7.20% | 5 | 1 | 1.60% | 3 |
| ADP-ribosylation factor 6 | 4502211 | 20 | 1 | 15% | 1 | 3 | 34% | 3 | 1 | 12% | 1 |
| iron-sulfur cluster assembly enzyme ISCU, mitochondrial isoform ISCU1 | 24307953 | 15 | 1 | 13% | 3 | 2 | 25% | 5 | 4 | 40% | 11 |
| threonine synthase-like 1 isoform X1 | 530392455 | 83 | 1 | 2.30% | 3 | 2 | 5.10% | 4 | 4 | 9.80% | 9 |
| guanine nucleotide-binding protein G(I)/G(S)/G(T) subunit beta-1 isoform 1 | 11321585 | 37 | 1 | 5.90% | 3 | 2 | 9.40% | 4 | 3 | 13% | 12 |
| calcyphosin isoform X1 | 530425453 | 30 | 1 | 5.50% | 2 | 2 | 9.10% | 2 | 3 | 13% | 5 |
| ribonuclease inhibitor | 21361547 | 50 | 1 | 7.20% | 3 | 2 | 12% | 4 | 3 | 18% | 8 |
| cathepsin B isoform X1 | 578815059 | 38 | 1 | 4.70% | 3 | 2 | 10% | 8 | 3 | 13% | 6 |
| 40S ribosomal protein S6 | 17158044 | 29 | 1 | 4.80% | 3 | 2 | 11% | 6 | 3 | 18% | 8 |
| amyloid beta A4 protein isoform h precursor | 324021738 | 85 | 1 | 4.40% | 3 | 2 | 5.70% | 4 | 3 | 5.10% | 6 |
| apolipoprotein E isoform X1 | 530416441 | 39 | 1 | 4.40% | 1 | 2 | 11% | 3 | 3 | 14% | 5 |
| calcium-binding tyrosine phosphorylation-regulated protein isoform c | 24797112 | 41 | 1 | 27% | 3 | 2 | 32% | 4 | 3 | 40% | 5 |
| protein FAM187B precursor | 22749005 | 42 | 1 | 2.70% | 2 | 2 | 9.50% | 4 | 3 | 12% | 6 |
| phosphoethanolamine/phosphocholine phosphatase isoform 1 | 219689097 | 32 | 1 | 8.20% | 6 | 2 | 13% | 7 | 2 | 13% | 7 |
| transmembrane and coiled-coil domain-containing protein 1 isoform a | 371872752 | 27 | 1 | 6.30% | 5 | 2 | 12% | 9 | 2 | 7.90% | 7 |
| COP9 signalosome complex subunit 7a isoform X1 | 530398666 | 30 | 1 | 5.10% | 3 | 2 | 11% | 7 | 2 | 9.50% | 3 |
| PRA1 family protein 2 | 6005794 | 19 | 1 | 6.20% | 6 | 2 | 16% | 10 | 2 | 16% | 7 |
| talin-1 | 223029410 | 270 | 1 | 0.83% | 1 | 2 | 2.20% | 2 | 2 | 1.80% | 2 |
| calcium and integrin-binding protein 1 isoform a | 480540325 | 26 | 1 | 6.50% | 3 | 2 | 14% | 7 | 2 | 14% | 4 |
| beta-centractin | 11342680 | 42 | 1 | 22% | 2 | 2 | 27% | 5 | 2 | 38% | 4 |
| ester hydrolase C11orf54 isoform c | 554506535 | 33 | 1 | 5.70% | 3 | 2 | 9.80% | 6 | 2 | 9.80% | 6 |
| spermatogenesis-associated protein 31C2 | 223468617 | 131 | 1 | 1.90% | 3 | 2 | 3.30% | 4 | 2 | 3.30% | 3 |
| NADH dehydrogenase [ubiquinone] 1 alpha subcomplex subunit 6 | 51317370 | 18 | 1 | 5.20% | 3 | 2 | 16% | 4 | 2 | 16% | 4 |
| desmoplakin isoform I | 58530840 | 332 | 1 | 0.59% | 1 | 2 | 1.30% | 6 | 2 | 1.30% | 4 |
| tetraspanin-1 | 21264578 | 26 | 1 | 5.40% | 3 | 2 | 7.50% | 6 | 2 | 7.50% | 4 |
| nicalin isoform X1 | 530425322 | 63 | 1 | 3.70% | 3 | 2 | 6.00% | 5 | 2 | 6.00% | 4 |
| NADH dehydrogenase subunit 5 (mitochondrion) | 251831117 | 67 | 1 | 3.30% | 2 | 2 | 8.00% | 7 | 2 | 8.00% | 6 |
| 40S ribosomal protein S12 | 14277700 | 15 | 1 | 6.10% | 2 | 2 | 28% | 3 | 2 | 23% | 6 |
| glycerophosphodiester phosphodiesterase 1 | 7706617 | 38 | 1 | 3.00% | 3 | 2 | 7.60% | 4 | 2 | 7.60% | 4 |
| OCIA domain-containing protein 1 isoform 4 | 269914128 | 28 | 1 | 5.20% | 3 | 2 | 12% | 4 | 2 | 16% | 4 |
| casein kinase I isoform gamma-1 | 98986450 | 49 | 1 | 2.60% | 1 | 2 | 5.90% | 4 | 2 | 5.90% | 4 |
| angiogenin precursor | 148277046 | 17 | 1 | 14% | 3 | 2 | 24% | 6 | 2 | 24% | 5 |
| ribosyldihydronicotinamide dehydrogenase [quinone] isoform X3 | 530382085 | 26 | 1 | 9.50% | 2 | 2 | 17% | 6 | 2 | 17% | 4 |
| 5'(3')-deoxyribonucleotidase, cytosolic type isoform 1 | 7657033 | 23 | 1 | 12% | 1 | 2 | 21% | 8 | 2 | 21% | 3 |
| synaptogyrin-4 | 22035702 | 26 | 1 | 6.40% | 18 | 2 | 16% | 25 | 1 | 6.40% | 19 |
| extracellular superoxide dismutase [Cu-Zn] precursor | 118582275 | 26 | 1 | 6.70% | 2 | 2 | 24% | 4 | 1 | 3.30% | 3 |
| endoplasmic reticulum-Golgi intermediate compartment protein 1 | 72534712 | 33 | 1 | 8.30% | 3 | 2 | 13% | 4 | 1 | 8.30% | 3 |
| protein Daple isoform X2 | 530403837 | 177 | 1 | 0.72% | 3 | 2 | 1.90% | 8 | 1 | 0.72% | 3 |
| V-type proton ATPase subunit d 1 | 19913432 | 40 | 1 | 5.10% | 1 | 2 | 6.80% | 4 | 1 | 3.10% | 3 |
| sorcin isoform C | 379317145 | 21 | 1 | 6.20% | 3 | 2 | 12% | 4 | 1 | 6.20% | 3 |
| 40S ribosomal protein S25 | 4506707 | 14 | 1 | 8.80% | 3 | 2 | 17% | 2 | 1 | 10% | 3 |
| oligosaccharyltransferase complex subunit OSTC isoform 1 | 24308271 | 17 | 1 | 4.70% | 1 | 2 | 13% | 4 | 1 | 8.10% | 4 |
| anoctamin-6 isoform c | 218156305 | 108 | 1 | 2.40% | 2 | 2 | 4.60% | 6 | 1 | 2.40% | 3 |
| aminoacyl tRNA synthase complex-interacting multifunctional protein 2 isoform X2 | 530384983 | 31 | 1 | 3.20% | 1 | 2 | 7.90% | 7 | 1 | 3.20% | 6 |
| attractin isoform 1 preproprotein | 21450861 | 159 | 1 | 1.10% | 3 | 2 | 2.00% | 4 | 1 | 1.10% | 3 |
| thioredoxin isoform 2 | 349732256 | 9 | 1 | 15% | 2 | 2 | 33% | 4 | 1 | 15% | 1 |
| 40S ribosomal protein S16 | 4506691 | 16 | 1 | 6.80% | 2 | 2 | 14% | 5 | 1 | 7.50% | 1 |
| coiled-coil-helix-coiled-coil-helix domain-containing protein 3, mitochondrial precursor | 8923390 | 26 | 1 | 4.80% | 3 | 2 | 11% | 5 | 1 | 4.80% | 3 |
| 60S ribosomal protein L36 | 16117794 | 12 | 1 | 9.50% | 1 | 2 | 22% | 5 | 1 | 12% | 1 |
| coatomer subunit zeta-1 isoform 1 | 7706337 | 20 | 1 | 13% | 1 | 2 | 12% | 3 | 1 | 13% | 2 |
| hornerin | 57864582 | 282 | 1 | 0.84% | 3 | 2 | 1.70% | 6 | 1 | 0.84% | 3 |
| gamma-glutamylcyclotransferase isoform 1 | 13129018 | 21 | 1 | 14% | 1 | 2 | 20% | 6 | 1 | 14% | 3 |
| adrenodoxin-like protein, mitochondrial precursor | 72534754 | 20 | 1 | 11% | 4 | 2 | 23% | 5 | 1 | 11% | 4 |
| NADPH:adrenodoxin oxidoreductase, mitochondrial isoform 7 | 384381471 | 48 | 1 | 5.00% | 3 | 2 | 8.70% | 4 | 1 | 5.00% | 3 |
| calmin | 19923599 | 112 | 1 | 2.40% | 3 | 2 | 5.10% | 4 | 1 | 2.40% | 3 |
| biotinidase isoform 1 | 528524481 | 61 | 1 | 3.10% | 1 | 2 | 5.50% | 6 | 1 | 3.10% | 2 |
| platelet-activating factor acetylhydrolase IB subunit alpha | 4557741 | 47 | 1 | 4.10% | 1 | 2 | 7.80% | 2 | 1 | 3.70% | 1 |
| UPF0577 protein KIAA1324 isoform 1 | 38569482 | 111 | 1 | 1.30% | 2 | 2 | 4.10% | 5 | ni | ni | ni |
| proteasome subunit beta type-10 precursor | 4506191 | 29 | 1 | 7.30% | 1 | 2 | 18% | 6 | ni | ni | ni |
| 45 calcium-binding protein isoform 2 precursor | 18699732 | 42 | 1 | 7.70% | 2 | 2 | 14% | 2 | ni | ni | ni |
| methylglutaconyl-CoA hydratase, mitochondrial precursor | 4502327 | 36 | 1 | 6.20% | 2 | 1 | 6.20% | 1 | 4 | 12% | 7 |
| dnaJ homolog subfamily C member 11 | 217035105 | 63 | 1 | 2.70% | 1 | 1 | 3.60% | 1 | 4 | 11% | 8 |
| lysosomal Pro-X carboxypeptidase isoform 2 preproprotein | 117306169 | 58 | 1 | 3.70% | 6 | 1 | 3.70% | 6 | 3 | 10% | 9 |
| NADH dehydrogenase [ubiquinone] 1 alpha subcomplex subunit 2 isoform 1 | 4505355 | 11 | 1 | 21% | 5 | 1 | 21% | 5 | 3 | 41% | 8 |
| NADH dehydrogenase [ubiquinone] 1 alpha subcomplex subunit 10, mitochondrial isoform X3 | 578804372 | 43 | 1 | 5.90% | 3 | 1 | 5.90% | 2 | 3 | 16% | 6 |
| ras-related C3 botulinum toxin substrate 1 isoform Rac1 | 9845511 | 21 | 1 | 15% | 1 | 1 | 15% | 3 | 3 | 27% | 8 |
| insulin-degrading enzyme isoform 1 | 155969707 | 118 | 1 | 0.88% | 1 | 1 | 1.20% | 1 | 3 | 4.40% | 5 |
| actin-related protein 2 isoform b | 5031571 | 45 | 1 | 9.10% | 1 | 1 | 9.10% | 3 | 3 | 18% | 6 |
| dnaJ homolog subfamily C member 10 isoform 1 precursor | 24308127 | 91 | 1 | 1.30% | 2 | 1 | 3.00% | 2 | 3 | 7.10% | 5 |
| dynein light chain Tctex-type 1 | 5730085 | 12 | 1 | 16% | 6 | 1 | 16% | 5 | 2 | 30% | 9 |
| beta-2-microglobulin precursor | 4757826 | 14 | 1 | 18% | 6 | 1 | 18% | 6 | 2 | 27% | 10 |
| alpha-2-antiplasmin isoform b precursor | 260064050 | 48 | 1 | 5.60% | 4 | 1 | 5.60% | 4 | 2 | 12% | 8 |
| histone H2B type 1-B | 10800140 | 14 | 1 | 40% | 1 | 1 | 40% | 1 | 2 | 41% | 5 |
| major prion protein preproprotein | 122056623 | 28 | 1 | 4.70% | 3 | 1 | 4.70% | 5 | 2 | 9.50% | 5 |
| actin-related protein 10 | 8923712 | 46 | 1 | 6.00% | 4 | 1 | 6.00% | 6 | 2 | 9.10% | 8 |
| tetraspanin-8 | 4759238 | 26 | 1 | 5.90% | 3 | 1 | 5.90% | 8 | 2 | 17% | 6 |
| LETM1 domain-containing protein 1 isoform X7 | 578823569 | 23 | 1 | 5.90% | 2 | 1 | 5.90% | 3 | 2 | 11% | 5 |
| GTP-binding protein SAR1b isoform X1 | 578810411 | 22 | 1 | 5.60% | 3 | 1 | 5.60% | 3 | 2 | 17% | 4 |
| lysozyme C precursor | 4557894 | 17 | 1 | 19% | 2 | 1 | 19% | 2 | 2 | 27% | 5 |
| protein transport protein Sec61 subunit beta | 5803165 | 10 | 1 | 10% | 2 | 1 | 10% | 1 | 2 | 26% | 4 |
| AP-1 complex subunit gamma-1 isoform b | 71772942 | 91 | 1 | 1.10% | 1 | 1 | 1.70% | 2 | 2 | 3.30% | 3 |
| LDLR chaperone MESD precursor | 74136552 | 26 | 1 | 7.30% | 1 | 1 | 7.30% | 3 | 2 | 13% | 2 |
| up-regulated during skeletal muscle growth protein 5 | 14249376 | 6 | 1 | 26% | 3 | 1 | 26% | 3 | 2 | 43% | 4 |
| eukaryotic translation initiation factor 6 isoform a | 31563378 | 27 | 1 | 9.80% | 3 | 1 | 9.80% | 3 | 2 | 17% | 4 |
| amiloride-sensitive amine oxidase [copper-containing] isoform X2 | 530386940 | 85 | 1 | 1.60% | 1 | 1 | 1.60% | 3 | 2 | 3.60% | 2 |
| NADH dehydrogenase [ubiquinone] iron-sulfur protein 4, mitochondrial precursor | 4505369 | 20 | 1 | 8.60% | 3 | 1 | 5.70% | 1 | 2 | 18% | 5 |
| coiled-coil domain-containing protein 42A isoform 1 | 226494053 | 38 | 1 | 5.10% | 3 | 1 | 5.10% | 3 | 2 | 11% | 2 |
| 40S ribosomal protein S9 | 14141193 | 23 | 1 | 3.60% | 1 | 1 | 4.60% | 3 | 2 | 7.70% | 2 |
| cytochrome b561 isoform X1 | 530411654 | 35 | 1 | 4.70% | 2 | 1 | 3.50% | 3 | 2 | 8.20% | 4 |
| aldehyde dehydrogenase family 16 member A1 isoform 1 | 223972651 | 85 | 1 | 1.60% | 3 | 1 | 1.60% | 1 | 2 | 3.10% | 4 |
| 2',3'-cyclic-nucleotide 3'-phosphodiesterase isoform X1 | 578830418 | 45 | 1 | 5.70% | 1 | 1 | 5.70% | 3 | 2 | 12% | 3 |
| vacuolar protein sorting-associated protein 33A | 18105056 | 68 | 1 | 5.00% | 1 | 1 | 5.00% | 1 | 2 | 7.40% | 4 |
| protein MROH8 isoform 1 | 194595501 | 119 | 1 | 2.80% | 1 | 1 | 2.80% | 2 | 2 | 3.90% | 2 |
| NAD-dependent protein deacylase sirtuin-5, mitochondrial isoform 2 | 13787215 | 33 | 1 | 7.40% | 1 | 1 | 5.40% | 1 | 2 | 10% | 2 |
| 40S ribosomal protein S17-like | 312284072 | 16 | 1 | 16% | 2 | 1 | 16% | 1 | 2 | 36% | 5 |
| protein YIPF6 isoform A | 304766239 | 26 | 1 | 5.90% | 1 | 1 | 5.90% | 1 | 2 | 5.90% | 2 |
| serine/threonine-protein kinase SMG1 isoform X1 | 530407763 | 414 | 1 | 0.19% | 2 | 1 | 0.19% | 5 | 1 | 0.19% | 3 |
| actin, cytoplasmic 1 | 4501885 | 42 | 1 | 84% | 6 | 1 | 78% | 1 | 1 | 79% | 3 |
| choline transporter-like protein 4 isoform 1 | 148612887 | 79 | 1 | 2.00% | 3 | 1 | 2.00% | 6 | 1 | 2.00% | 3 |
| cytoplasmic dynein 1 intermediate chain 2 isoform 1 | 422398885 | 71 | 1 | 4.20% | 6 | 1 | 4.20% | 3 | 1 | 4.20% | 6 |
| histone H2A type 2-C | 24638446 | 14 | 1 | 44% | 3 | 1 | 44% | 3 | 1 | 50% | 3 |
| IST1 homolog isoform a | 401709930 | 40 | 1 | 5.20% | 3 | 1 | 5.20% | 3 | 1 | 5.20% | 3 |
| alpha-1-acid glycoprotein 1 precursor | 167857790 | 24 | 1 | 7.00% | 3 | 1 | 7.00% | 2 | 1 | 7.00% | 1 |
| integrin beta-1 isoform 1A precursor | 19743813 | 88 | 1 | 2.60% | 1 | 1 | 2.60% | 2 | 1 | 2.60% | 2 |
| beta-hexosaminidase subunit beta preproprotein | 4504373 | 63 | 1 | 4.70% | 5 | 1 | 4.70% | 3 | 1 | 4.70% | 3 |
| 60S ribosomal protein L35 | 6005860 | 15 | 1 | 8.10% | 3 | 1 | 8.10% | 3 | 1 | 8.10% | 1 |
| carbonyl reductase family member 4 | 40254992 | 25 | 1 | 5.10% | 3 | 1 | 5.10% | 3 | 1 | 5.10% | 3 |
| glutathione S-transferase Mu 1 isoform 1 | 23065544 | 26 | 1 | 6.00% | 1 | 1 | 6.00% | 3 | 1 | 7.80% | 3 |
| phosphatidylinositol transfer protein alpha isoform | 5453908 | 32 | 1 | 8.50% | 1 | 1 | 8.50% | 3 | 1 | 8.50% | 3 |
| four and a half LIM domains protein 1 isoform X1 | 578838645 | 38 | 1 | 3.50% | 3 | 1 | 3.50% | 1 | 1 | 3.50% | 2 |
| mitochondrial import receptor subunit TOM20 homolog | 7657257 | 16 | 1 | 14% | 2 | 1 | 14% | 4 | 1 | 14% | 3 |
| sigma non-opioid intracellular receptor 1 isoform 1 | 5032117 | 25 | 1 | 15% | 1 | 1 | 15% | 1 | 1 | 8.10% | 1 |
| mitochondrial inner membrane organizing system protein 1 isoform c | 323276670 | 15 | 1 | 23% | 3 | 1 | 23% | 3 | 1 | 23% | 3 |
| tubulin beta-6 chain | 14210536 | 50 | 1 | 21% | 1 | 1 | 22% | 1 | 1 | 24% | 7 |
| 4-aminobutyrate aminotransferase, mitochondrial precursor | 38679950 | 56 | 1 | 3.00% | 4 | 1 | 3.00% | 2 | 1 | 3.00% | 3 |
| cell cycle control protein 50A isoform 1 | 8922720 | 41 | 1 | 2.20% | 1 | 1 | 3.60% | 1 | ni | ni | ni |
| apolipoprotein D precursor | 4502163 | 21 | 1 | 5.80% | 3 | 1 | 4.80% | 1 | ni | ni | ni |
| neutrophil elastase isoform X1 | 530425159 | 29 | 1 | 10% | 1 | 1 | 10% | 3 | ni | ni | ni |
| sideroflexin-1 | 23618867 | 36 | 1 | 11% | 2 | 1 | 11% | 3 | ni | ni | ni |
| NADH dehydrogenase subunit 1 (mitochondrion) | 251831107 | 36 | 1 | 6.30% | 3 | 1 | 6.30% | 3 | ni | ni | ni |
| UPF0669 protein C6orf120 precursor | 71143156 | 21 | 1 | 14% | 3 | 1 | 14% | 3 | ni | ni | ni |
| spermatogenesis-associated protein 31E1 | 155029550 | 157 | 1 | 0.76% | 1 | ni | ni | ni | 2 | 1.20% | 3 |
| V-type proton ATPase subunit D | 7706757 | 28 | 1 | 6.50% | 2 | ni | ni | ni | 2 | 13% | 4 |
| 2-oxoisovalerate dehydrogenase subunit alpha, mitochondrial isoform 2 precursor | 258645172 | 50 | 1 | 3.60% | 1 | ni | ni | ni | 2 | 7.00% | 4 |
| 60S ribosomal protein L31 isoform 2 | 148746199 | 15 | 1 | 11% | 3 | ni | ni | ni | 2 | 18% | 4 |
| peflin | 6912582 | 30 | 1 | 11% | 1 | ni | ni | ni | 2 | 17% | 3 |
| platelet-activating factor acetylhydrolase IB subunit beta isoform a | 4505585 | 26 | 1 | 8.30% | 1 | ni | ni | ni | 2 | 12% | 5 |
| multiple inositol polyphosphate phosphatase 1 isoform 1 precursor | 19923761 | 55 | 1 | 6.00% | 3 | ni | ni | ni | 2 | 8.20% | 3 |
| prenylcysteine oxidase-like precursor | 226442763 | 55 | 1 | 4.90% | 1 | ni | ni | ni | 2 | 7.10% | 2 |
| iron-sulfur cluster assembly 2 homolog, mitochondrial isoform 1 precursor | 160420328 | 16 | 1 | 7.80% | 2 | ni | ni | ni | 2 | 16% | 3 |
| uncharacterized protein C20orf85 | 30425380 | 16 | 1 | 13% | 1 | ni | ni | ni | 2 | 27% | 5 |
| armadillo repeat-containing protein 4 isoform X3 | 578818430 | 112 | 1 | 1.40% | 5 | ni | ni | ni | 1 | 1.40% | 1 |
| protein GNAS isoform XLas | 117938759 | 111 | 1 | 1.10% | 2 | ni | ni | ni | 1 | 1.10% | 2 |
| UPF0556 protein C19orf10 precursor | 33457348 | 19 | 1 | 8.70% | 1 | ni | ni | ni | 1 | 8.70% | 3 |
| vinculin isoform VCL | 4507877 | 117 | 1 | 2.00% | 3 | ni | ni | ni | 1 | 2.00% | 1 |
| translocation protein SEC63 homolog | 6005872 | 88 | 1 | 1.70% | 1 | ni | ni | ni | 1 | 1.70% | 1 |
| V-type proton ATPase subunit E 1 isoform a | 4502317 | 26 | 1 | 10% | 1 | ni | ni | ni | 1 | 10% | 3 |
| major histocompatibility complex, class II, DR beta 5 precursor | 18641375 | 30 | 1 | 6.40% | 1 | ni | ni | ni | 1 | 6.40% | 2 |
| probable phospholipid-transporting ATPase IA isoform b | 157649069 | 130 | 1 | 1.00% | 3 | ni | ni | ni | 1 | 1.00% | 3 |
| sialic acid synthase | 12056473 | 40 | 1 | 7.80% | 2 | ni | ni | ni | 1 | 7.80% | 2 |
| selenoprotein S isoform 2 | 33285002 | 21 | 1 | 5.80% | 3 | ni | ni | ni | 1 | 5.80% | 1 |
| sterol-4-alpha-carboxylate 3-dehydrogenase, decarboxylating isoform X1 | 578838867 | 42 | 1 | 5.10% | 1 | ni | ni | ni | 1 | 4.80% | 2 |
| actin-related protein 2/3 complex subunit 3 isoform 1 | 514052670 | 21 | 1 | 6.20% | 1 | ni | ni | ni | 1 | 6.20% | 1 |
| ras-related protein Rab-3D | 4759000 | 24 | 1 | 13% | 3 | ni | ni | ni | ni | ni | ni |
| V-type proton ATPase subunit H isoform 2 | 47717100 | 54 | 1 | 4.90% | 1 | ni | ni | ni | ni | ni | ni |
| argininosuccinate lyase isoform 2 | 68303547 | 50 | 1 | 2.50% | 2 | ni | ni | ni | ni | ni | ni |
| xaa-Pro dipeptidase isoform 1 | 149589008 | 55 | 1 | 2.00% | 3 | ni | ni | ni | ni | ni | ni |
| sulfate transporter | 100913030 | 82 | ni | ni | ni | 4 | 8.70% | 8 | 2 | 4.60% | 5 |
| putative peptidyl-tRNA hydrolase PTRHD1 | 61966781 | 16 | ni | ni | ni | 3 | 41% | 7 | 5 | 47% | 5 |
| hydroxysteroid dehydrogenase-like protein 2 isoform 1 | 40254986 | 45 | ni | ni | ni | 3 | 13% | 8 | 4 | 16% | 12 |
| glycogen phosphorylase, brain form isoform X1 | 578835493 | 97 | ni | ni | ni | 3 | 4.90% | 6 | 3 | 4.90% | 7 |
| NADH dehydrogenase subunit 4 (mitochondrion) | 251831116 | 52 | ni | ni | ni | 3 | 9.80% | 8 | 2 | 5.70% | 4 |
| prolactin regulatory element-binding protein | 7019503 | 45 | ni | ni | ni | 3 | 17% | 6 | 2 | 10% | 4 |
| dynein heavy chain 12, axonemal isoform X1 | 578805765 | 455 | ni | ni | ni | 3 | 1.20% | 3 | 1 | 0.53% | 1 |
| histone H2A.Z | 4504255 | 14 | ni | ni | ni | 3 | 48% | 3 | ni | ni | ni |
| nuclear pore complex protein Nup98-Nup96 isoform 4 | 56549645 | 187 | ni | ni | ni | 2 | 1.70% | 4 | 3 | 2.40% | 5 |
| orphan sodium- and chloride-dependent neurotransmitter transporter NTT5 isoform X1 | 530416341 | 91 | ni | ni | ni | 2 | 5.40% | 3 | 3 | 8.10% | 7 |
| endothelial lipase isoform X1 | 530414043 | 61 | ni | ni | ni | 2 | 6.50% | 4 | 2 | 6.50% | 3 |
| 60S acidic ribosomal protein P0 | 4506667 | 34 | ni | ni | ni | 2 | 7.90% | 3 | 2 | 10% | 2 |
| nuclear pore complex protein Nup205 | 57634534 | 228 | ni | ni | ni | 2 | 1.40% | 4 | 2 | 2.10% | 2 |
| huntingtin-interacting protein 1 isoform X2 | 530385819 | 113 | ni | ni | ni | 2 | 6.20% | 4 | 2 | 5.60% | 2 |
| ras GTPase-activating-like protein IQGAP1 | 4506787 | 189 | ni | ni | ni | 2 | 2.20% | 4 | 2 | 2.20% | 3 |
| aldehyde oxidase | 71773480 | 148 | ni | ni | ni | 2 | 2.50% | 6 | 1 | 1.60% | 1 |
| testis-expressed sequence 29 protein | 22748709 | 17 | ni | ni | ni | 2 | 19% | 3 | 1 | 7.30% | 2 |
| prolactin precursor | 4506105 | 26 | ni | ni | ni | 2 | 11% | 2 | 1 | 6.20% | 1 |
| protein QIL1 precursor | 45387955 | 13 | ni | ni | ni | 2 | 44% | 4 | 1 | 17% | 1 |
| lysosomal acid phosphatase isoform 1 precursor | 4557010 | 48 | ni | ni | ni | 2 | 5.20% | 4 | 1 | 2.60% | 3 |
| calcium-binding mitochondrial carrier protein SCaMC-1 isoform 2 | 47458041 | 51 | ni | ni | ni | 2 | 10% | 2 | 1 | 3.50% | 1 |
| ras-related protein Rab-5B isoform X2 | 530400630 | 24 | ni | ni | ni | 2 | 20% | 7 | 1 | 14% | 1 |
| cysteine-rich with EGF-like domain protein 2 isoform a precursor | 205360956 | 44 | ni | ni | ni | 2 | 6.20% | 4 | 1 | 3.20% | 3 |
| 6-phosphofructokinase, liver type isoform b | 48762920 | 85 | ni | ni | ni | 2 | 5.40% | 3 | 1 | 4.00% | 1 |
| 60S ribosomal protein L30 | 4506631 | 13 | ni | ni | ni | 2 | 24% | 4 | 1 | 10% | 2 |
| late cornified envelope-like proline-rich protein 1 | 58082087 | 11 | ni | ni | ni | 2 | 42% | 5 | 1 | 24% | 1 |
| nicotinamide phosphoribosyltransferase isoform X1 | 530385393 | 56 | ni | ni | ni | 2 | 10% | 4 | 1 | 5.90% | 2 |
| monoacylglycerol lipase ABHD12 isoform X3 | 530425689 | 30 | ni | ni | ni | 2 | 16% | 4 | 1 | 7.10% | 3 |
| protein SCO1 homolog, mitochondrial isoform X1 | 530410547 | 22 | ni | ni | ni | 2 | 16% | 3 | 1 | 11% | 3 |
| beta-2-glycoprotein 1 precursor | 153266841 | 38 | ni | ni | ni | 2 | 11% | 4 | 1 | 4.10% | 3 |
| tubulin-specific chaperone D | 41350333 | 133 | ni | ni | ni | 2 | 4.50% | 2 | 1 | 2.20% | 2 |
| acyl-coenzyme A thioesterase THEM4 | 76159293 | 27 | ni | ni | ni | 2 | 9.20% | 3 | 1 | 5.00% | 3 |
| outer dense fiber protein 3B | 116292180 | 27 | ni | ni | ni | 2 | 13% | 2 | 1 | 9.90% | 1 |
| disintegrin and metalloproteinase domain-containing protein 2 isoform 1 preproprotein | 55743080 | 82 | ni | ni | ni | 2 | 3.80% | 3 | ni | ni | ni |
| nuclear transport factor 2 | 5031985 | 14 | ni | ni | ni | 2 | 26% | 2 | ni | ni | ni |
| ras-related protein Rab-11A isoform 1 | 4758984 | 24 | ni | ni | ni | 2 | 62% | 4 | ni | ni | ni |
| leucine-rich repeat-containing protein 18 | 167466264 | 30 | ni | ni | ni | 2 | 12% | 3 | ni | ni | ni |
| glucosamine-6-phosphate isomerase 1 isoform X1 | 530380347 | 36 | ni | ni | ni | 1 | 5.30% | 1 | 4 | 20% | 7 |
| RPL17-C18orf32 protein isoform 1 | 313569822 | 26 | ni | ni | ni | 1 | 4.40% | 1 | 2 | 11% | 2 |
| ras GTPase-activating-like protein IQGAP2 isoform 2 | 550544224 | 174 | ni | ni | ni | 1 | 1.40% | 3 | 2 | 2.50% | 7 |
| AP-2 complex subunit beta isoform b | 4557469 | 105 | ni | ni | ni | 1 | 5.20% | 3 | 2 | 10% | 2 |
| beta-hexosaminidase subunit alpha preproprotein | 189181666 | 61 | ni | ni | ni | 1 | 3.60% | 1 | 2 | 5.70% | 3 |
| dnaJ homolog subfamily C member 13 isoform X1 | 530374184 | 255 | ni | ni | ni | 1 | 0.53% | 1 | 1 | 0.62% | 1 |
| proteasome subunit beta type-8 isoform E2 proprotein | 73747875 | 30 | ni | ni | ni | 1 | 5.10% | 1 | 1 | 4.00% | 3 |
| prefoldin subunit 3 | 4507873 | 23 | ni | ni | ni | 1 | 13% | 2 | 1 | 5.10% | 1 |
| glutamine-dependent NAD(+) synthetase | 41393551 | 79 | ni | ni | ni | 1 | 2.40% | 2 | 1 | 2.40% | 3 |
| alpha-mannosidase 2x | 51477716 | 131 | ni | ni | ni | 1 | 1.00% | 3 | 1 | 1.00% | 1 |
| coatomer subunit epsilon isoform a | 31542319 | 34 | ni | ni | ni | 1 | 8.40% | 2 | 1 | 13% | 1 |
| eukaryotic translation initiation factor 3 subunit E | 4503521 | 52 | ni | ni | ni | 1 | 2.20% | 3 | 1 | 2.20% | 3 |
| synaptotagmin-7 isoform X1 | 530397569 | 76 | ni | ni | ni | 1 | 2.60% | 1 | 1 | 2.60% | 1 |
| ectonucleoside triphosphate diphosphohydrolase 2 isoform 2 | 4557421 | 51 | ni | ni | ni | 1 | 5.10% | 2 | 1 | 5.10% | 4 |
| kallikrein-2 isoform 1 preproprotein | 5031829 | 29 | ni | ni | ni | 1 | 16% | 2 | 1 | 16% | 1 |
| protein canopy homolog 3 precursor | 33942072 | 31 | ni | ni | ni | 1 | 6.50% | 3 | 1 | 6.50% | 3 |
| mannose-1-phosphate guanyltransferase alpha | 31881779 | 46 | ni | ni | ni | 1 | 3.60% | 3 | 1 | 6.70% | 3 |
| amyloid-like protein 2 isoform 2 precursor | 214010181 | 85 | ni | ni | ni | 1 | 2.70% | 1 | 1 | 2.70% | 1 |
| propionyl-CoA carboxylase beta chain, mitochondrial isoform 1 precursor | 119943100 | 58 | ni | ni | ni | 1 | 5.80% | 3 | 1 | 5.80% | 3 |
| basement membrane-specific heparan sulfate proteoglycan core protein precursor | 126012571 | 469 | ni | ni | ni | 1 | 0.25% | 1 | ni | ni | ni |
| inositol monophosphatase 3 | 157388900 | 39 | ni | ni | ni | 1 | 8.10% | 2 | ni | ni | ni |
| peptidase M20 domain-containing protein 2 | 58082085 | 48 | ni | ni | ni | 1 | 6.70% | 3 | ni | ni | ni |
| guanylate cyclase soluble subunit beta-1 | 4504215 | 71 | ni | ni | ni | 1 | 1.90% | 3 | ni | ni | ni |
| carboxypeptidase M precursor | 38327526 | 51 | ni | ni | ni | 1 | 1.80% | 1 | ni | ni | ni |
| NADH dehydrogenase [ubiquinone] 1 beta subcomplex subunit 5, mitochondrial isoform 2 | 316660086 | 16 | ni | ni | ni | 1 | 11% | 1 | ni | ni | ni |
| phosphoribosyl pyrophosphate synthase-associated protein 2 isoform 4 | 345478663 | 31 | ni | ni | ni | 1 | 6.00% | 1 | ni | ni | ni |
| protein RER1 | 116812591 | 23 | ni | ni | ni | 1 | 9.20% | 3 | ni | ni | ni |
| threonine--tRNA ligase, cytoplasmic isoform 1 | 38202255 | 83 | ni | ni | ni | 1 | 4.10% | 1 | ni | ni | ni |
| carboxylesterase 5A isoform 2 precursor | 21450749 | 58 | ni | ni | ni | 1 | 3.40% | 3 | ni | ni | ni |
| syntaxin-3 isoform X7 | 578821695 | 34 | ni | ni | ni | ni | ni | ni | 2 | 9.20% | 5 |
| isoleucine--tRNA ligase, cytoplasmic | 94721241 | 145 | ni | ni | ni | ni | ni | ni | 2 | 1.60% | 4 |
| STE20/SPS1-related proline-alanine-rich protein kinase | 115430252 | 59 | ni | ni | ni | ni | ni | ni | 2 | 10% | 2 |
| endoplasmic reticulum metallopeptidase 1 isoform X1 | 530390160 | 87 | ni | ni | ni | ni | ni | ni | 2 | 2.30% | 4 |
| sodium/glucose cotransporter 1 isoform 2 | 372622382 | 60 | ni | ni | ni | ni | ni | ni | 2 | 5.00% | 4 |
| peptide-N(4)-(N-acetyl-beta-glucosaminyl)asparagine amidase isoform 2 | 223941798 | 72 | ni | ni | ni | ni | ni | ni | 2 | 3.50% | 2 |
| thioredoxin domain-containing protein 12 precursor | 7705696 | 19 | ni | ni | ni | ni | ni | ni | 2 | 14% | 2 |
| major histocompatibility complex, class II, DR beta 1 precursor | 155030211 | 30 | ni | ni | ni | ni | ni | ni | 1 | 9.80% | 2 |
| protein NDRG3 isoform X2 | 578836095 | 42 | ni | ni | ni | ni | ni | ni | 1 | 5.80% | 2 |
| cystatin-B | 4503117 | 11 | ni | ni | ni | ni | ni | ni | 1 | 12% | 2 |
| cation-independent mannose-6-phosphate receptor precursor | 119964726 | 274 | ni | ni | ni | ni | ni | ni | 1 | 0.52% | 1 |
| ceruloplasmin precursor | 4557485 | 122 | ni | ni | ni | ni | ni | ni | 1 | 1.20% | 1 |
| integrin beta-2 isoform X1 | 578836536 | 77 | ni | ni | ni | ni | ni | ni | 1 | 2.60% | 1 |
| WD repeat-containing protein 60 | 155030242 | 123 | ni | ni | ni | ni | ni | ni | 1 | 1.60% | 1 |
| homogentisate 1,2-dioxygenase isoform X2 | 530374530 | 38 | ni | ni | ni | ni | ni | ni | 1 | 7.10% | 1 |
| selenium-binding protein 1 isoform 1 | 16306550 | 52 | ni | ni | ni | ni | ni | ni | 1 | 4.70% | 1 |
| neutrophil defensin 1 precursor | 124248516 | 10 | ni | ni | ni | ni | ni | ni | 1 | 9.60% | 2 |
| neurofilament heavy polypeptide | 32483416 | 112 | ni | ni | ni | ni | ni | ni | 1 | 1.60% | 1 |
| hydroxymethylglutaryl-CoA lyase, mitochondrial isoform 1 precursor | 62198232 | 34 | ni | ni | ni | ni | ni | ni | 1 | 4.30% | 4 |
| NADH dehydrogenase (ubiquinone) complex I, assembly factor 6 precursor | 124517691 | 38 | ni | ni | ni | ni | ni | ni | 1 | 4.80% | 1 |
| isoamyl acetate-hydrolyzing esterase 1 homolog precursor | 88853865 | 28 | ni | ni | ni | ni | ni | ni | 1 | 4.80% | 1 |
| oxysterol-binding protein-related protein 9 isoform c | 22547171 | 70 | ni | ni | ni | ni | ni | ni | 1 | 0.96% | 1 |
| alpha-galactosidase A precursor | 4504009 | 49 | ni | ni | ni | ni | ni | ni | 1 | 2.80% | 2 |
